# Integration of multi-omics data uncovers novel germline susceptibility candidates in early-onset colorectal cancer

**DOI:** 10.1038/s41431-025-02004-7

**Published:** 2026-01-15

**Authors:** Anael López-Novo, Jorge Amigo, Andrés Dacal, David Remedios-Espino, Joaquín Cubiella, María Victoria Álvarez-Sánchez, María Jesús Ladra-González, Fernando Fernández-López, Ana Álvarez-Castro, Silvia Carlés, José Manuel Cameselle-Teijeiro, Miriam Cuatrecasas, Francesc Balaguer, Sergi Castellví-Bel, Ceres Fernández-Rozadilla, Ángel Carracedo, Clara Ruiz-Ponte

**Affiliations:** 1 Fundación Pública Galega de Medicina Xenómica (SERGAS), Instituto de Investigación Sanitaria de Santiago, GMX-University of Santiago de Compostela, Santiago de Compostela, Spain; 2 Centro de Investigación Biomédica en Red, Enfermedades Raras (CIBERER), Instituto de Salud Carlos III (ISCIII), CiMUS, Genomics and Bioinformatics Group, University of Santiago de Compostela, Santiago de Compostela, Spain; 3 Department of Gastroenterology, Hospital Lucus Augusti, Lugo, Spain; 4 Department of Gastroenterology, CHUO, CIBEREHD, Ourense, Spain; 5 Department of Gastroenterology, CHOP, Pontevedra, Spain; 6 Digestive Surgery Department, Coloproctology Unit, CHUS, Santiago de Compostela, Spain; 7 Department of Gastroenterology, CHUS, Santiago de Compostela, Spain; 8 Cancer Predisposition and Biomarkers Lab, Instituto de Investigación Sanitaria de Santiago de Compostela, Santiago de Compostela, Spain; 9 Department of Pathology, CHUS, University of Santiago de Compostela, Santiago de Compostela, Spain; 10 Department of Pathology, Hospital Clinic de Barcelona, Institut d’Investigacions Biomèdiques August Pi i Sunyer (IDIBAPS), CIBEREHD and Tumour Bank-Biobank, Barcelona, Spain; 11 Department of Gastroenterology, Institut d’Investigacions Biomèdiques August Pi i Sunyer (IDIBAPS), CIBEREHD, Hospital Clínic, Barcelona, Spain

**Keywords:** Colorectal cancer, Cancer genetics

## Abstract

Colorectal cancer (CRC) is increasingly diagnosed in individuals under 50 years of age, yet the underlying genetic predisposition remains largely unexplained, particularly in mismatch repair (MMR)-proficient cases. This study aimed to identify novel hereditary CRC susceptibility genes by integrating germline and tumour whole-exome sequencing (WES) with transcriptomic profiling across a cohort of early-onset CRC (EOCRC) patients. Tumours were categorised using Consensus Molecular Subtypes (CMS) classification and analysed for mutational signature and burden. We used a novel ‘All vs One’ multi-omic integration approach to identify loss-of-function rare germline variants with concordant gene expression alterations in tumour tissue. Five candidate genes (*ADCY4*, *NOXO1, CDHR2*, *ARHGAP10*, *EEF2K*) were prioritised based on this approach and potential biological relevance in CRC. These findings highlight the molecular heterogeneity of EOCRC and demonstrate the utility of multi-omic approaches in refining germline variant interpretation. Integrating tumour transcriptomics enhances gene discovery efforts and supports a more comprehensive understanding of CRC heritability in younger individuals.

**Figure:**
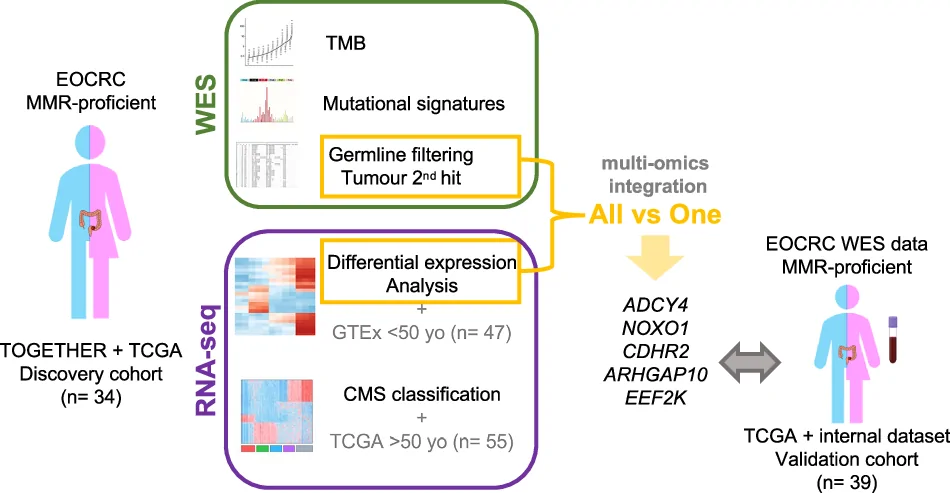

## Introduction

Colorectal cancer (CRC) is the third most commonly diagnosed malignancy and the second leading cause of cancer-related mortality worldwide in both men and women [1]. In recent years, a decline in CRC incidence has been observed among individuals aged 50 years and older, largely due to the implementation of population-based screening programs [2]. In contrast, a rising number of cases has been reported in individuals under the age of 50, a group not routinely included in current screening strategies [2].

CRC develops through a well-characterised sequence of clinical and histopathological stages, underpinned by a series of genetic alterations. These include the somatic activation of oncogenes and the inactivation of tumour suppressor genes. Initially, three major tumorigenic pathways were described: chromosomal instability, microsatellite instability, and the CpG island methylator phenotype [3]. However, these pathways are not mutually exclusive, and ongoing research continues to uncover alternative mechanisms and novel clinical entities [4].

A significant advancement in understanding CRC pathogenesis came in 2013 with the identification and cataloguing of cancer mutational signatures associated either with mismatch repair deficiency alone or with the concurrent presence of polymerase ε or δ1 mutations and mismatch repair defects [5]. These signatures provided insight into the specific pathogenic mechanisms underlying distinct mutational patterns and, to date, have enabled the identification of approximately twenty signatures associated with various carcinogenic processes in CRC [5–13]. In 2015, Guinney and colleagues proposed a new classification of CRC based on molecular profiling rather than histopathological features, known as the Consensus Molecular Subtypes (CMS) [14, 15]. While CMS currently offers the most biologically interpretable classification system, ongoing refinement is expected as additional molecular and histopathological data become available.

The heritability of CRC is estimated at up to 35% [16]. This genetic predisposition is explained by the presence of germline variants ranging from very rare, high-penetrance mutations, responsible for Mendelian CRC predisposition syndromes, to common, low-penetrance variants identified primarily through genome-wide association studies, as well as variants yet to be discovered [4, 17, 18]. Despite substantial progress and the growing use of next-generation sequencing technologies that have enabled the identification of novel genes [19–21], a significant proportion of CRC heritability remains unexplained. According to the “rare variant - common disease” hypothesis, part of this missing heritability may be attributable to rare variants that predispose to non-Mendelian forms of CRC but share phenotypic characteristics with Mendelian syndromes, such as familial aggregation and early-onset disease [4].

The present study aims to identify novel hereditary CRC predisposition genes through the integration of multi-omic technologies, thereby contributing to the reduction of unexplained heritability in this disease.

## Materials and methods

### Patients and samples

#### Discovery cohort

Nineteen early-onset CRC (EOCRC) patients (≤50 years) without polyposis from unrelated families were selected to form the TOGETHER cohort. All displayed mismatch repair (MMR)-proficient tumours, assessed as the conserved expression of the four MMR proteins (MLH1, MSH2, MSH6 and PMS2) by immunohistochemistry. The clinical features of the patients are described in Table 1.

**Table 1:** Clinical characteristics of patients from the TOGETHER and TCGA (discovery subset) cohorts.

Patient ID	CRC diagnosis age (range)	Sex	Tumour site	Tumor stage	Family cancer history
102	45–50	Female	Sigmoid colon	IV	FDR – melanoma
103	45–50	Male	Rectum	IV	None
104	40–44	Male	Rectum	IV	None
105	45–50	Male	Sigmoid colon	III	SDR – multiple cancers (ovarian, breast)
107	45–50	Female	Ascending colon	III	FDR – multiple cancers (prostate, colorectal)
108	45–50	Male	Sigmoid colon	IV	SDR – colorectal cancer
109	45–50	Male	Rectum	III	Multiple relatives (FDR & SDR) – multiple cancers
204	40–44	Male	Transverse colon	II	SDR – cancer (type not specified)
209	45–50	Male	Sigmoid colon	III	FDR & SDR – multiple cancers
302	45–50	Male	Sigmoid colon	III	None
303	45–50	Female	Rectum	II	None
304	<40	Male	Rectum	IV	TDR – colorectal cancer (multiple cases)
305	45-50	Female	Sigmoid colon	III	Multiple relatives (FDR & SDR) – multiple cancers
307	45–50	Female	Rectosigmoid junction	IV	None
308	45–50	Female	Rectosigmoid junction	II	FDR – gastric cancer
310	45–50	Male	Sigmoid colon	I	None
401	40–44	Male	Cecum	III	FDR & SDR – multiple cancers
402	45–50	Male	Rectum	III	SDR – cancer (type not specified)
501	40–44	Male	Sigmoid colon	II	Multiple relatives (FDR & SDR) – multiple cancers
TCGA-A6-5662	45–50	Male	Splenic flexure	IV	None
TCGA-A6-5667	40–44	Female	Sigmoid colon	II	None
TCGA-AZ-4323	<40	Male	Cecum	IV	Unknown
TCGA-AZ-4684	45–50	Male	Not specified	IV	Unknown
TCGA-AZ-5403	40–44	Male	Sigmoid colon	II	None
TCGA-CK-4947	45–50	Female	Sigmoid colon	III	Unknown
TCGA-CK-4948	45–50	Female	Sigmoid colon	III	Unknown
TCGA-CK-6748	45–50	Female	Sigmoid colon	IV	Unknown
TCGA-CM-5860	40–44	Male	Ascending colon	II	None
TCGA-CM-6161	<40	Female	Sigmoid colon	I	None
TCGA-CM-6162	45–50	Female	Ascending colon	III	Yes – type not specified
TCGA-CM-6164	45–50	Female	Sigmoid colon	II	Yes – type not specified
TCGA-CM-6166	45-50	Female	Ascending colon	I	None
TCGA-CM-6674	<40	Male	Hepatic flexure	II	Yes – type not specified
TCGA-D5-6539	45–50	Female	Transverse colon	Not specified	Unknown
TCGA-D5-6927	<40	Male	Transverse colon	II	Yes – type not specified
TCGA-D5-6929	45–50	Female	Sigmoid colon	IV	Yes – type not specified
TCGA-F4-6856	45–50	Male	Cecum	I	None
TCGA-AH-6643	45-50	Male	Rectosigmoid junction	III	None
TCGA-AH-6897	45–50	Male	Rectosigmoid junction	I	None
TCGA-CI-6620	40–44	Female	Rectum	IV	Unknown
TCGA-DC-4745	45–50	Female	Rectosigmoid junction	III	Yes – type not specified
TCGA-DC-6683	40–44	Male	Rectosigmoid junction	III	None
TCGA-EI-6508	45–50	Female	Rectum	III	Yes – type not specified
TCGA-EI-6917	<40	Male	Rectum	III	Unknown
TCGA-EI-7004	<40	Female	Rectosigmoid junction	Not specified	None

*FDR* first-degree relative, *SDR* second-degree relative, *TDR* third-degree relative.

Matched normal colonic mucosa and tumour tissue samples were collected from each patient at a distance of 10–15 cm within the same colonic segment. Tumour specimens were all treatment-naïve. Samples were preserved either in RNAlater RNA Stabilisation Reagent (Qiagen, Hilden, Germany) or flash-frozen. DNA and RNA were co-extracted using the AllPrep DNA/RNA/miRNA Universal Kit (Qiagen), following the manufacturer’s protocol.

*TCGA cohort (discovery subset)*: Paired normal-tumour whole-exome sequencing (WES) and tumour RNA sequencing (RNA-seq) data were obtained for 26 non-Finnish European EOCRC patients without polyposis, irrespective of MMR status due to inconsistencies in the available information, from the COAD and READ cohorts (Table 1).

To increase the statistical power of RNA analyses and to meet the minimum sample size required for CMS classification using the CMSCaller v0.99.2 R package (see Tumour Molecular Profiling section below), RNA-seq data were also retrieved from an additional 55 non-Finnish European CRC patients aged 51–65 years from TCGA COAD and READ cohorts (Supplementary Table 1). These patients were not included in the discovery subset.

#### GTEx cohort

RNA-seq data from normal colonic mucosa were obtained for 47 individuals aged ≤50 years with no personal history of CRC (Supplementary Table 2). These samples were included to increase the biological variability of normal colonic mucosa and were used in the ‘All vs One’ approach (see *Differential Expression and Variant Impact Analysis: ‘All vs One’ approach* section below) together with the normal colonic mucosa from patients in the TOGETHER cohort.

#### Replication cohort

To validate the candidate genes identified in the discovery cohort, a replication cohort comprising 39 unrelated EOCRC non-polyposis patients with MMR-proficient tumours and no available RNA-seq data was assembled. This group included nineteen patients from the TCGA (COAD and READ), all non-Finnish Europeans, and twenty patients from an internal clinical series, which has been previously described [22] (Supplementary Table 3). In all cases germline WES data derived from peripheral blood.

### Next-generation sequencing

#### WES

WES was performed on genomic DNA from paired normal and tumour mucosa in the TOGETHER cohort. Library preparation was carried out using the SureSelect human All Exon v5 (Agilent Technologies, Santa Clara, CA, USA), and sequencing was conducted at the Spanish National Centre for Genomic Analysis (CNAG, Barcelona, Spain) on the Illumina HiSeq3000 platform (Illumina Inc., California, USA). The average exome sequencing depth was 150X, and paired-end sequencing was performed with 2×101 base pair reads. Sequencing specifications for TCGA samples have been previously described elsewhere [23].

Sequencing reads from both TOGETHER and TCGA patients were aligned to the GRCh37/hg19 human reference genome using Burrows-Wheeler Aligner (BWA-MEM version 0.7.17). Variant calling of short mutations (single nucleotide variants and insertion and deletions) was performed using GATK 4.3.0.0. HaplotypeCaller was used for germline variant detection in normal mucosa, and Mutect2 was used for somatic variant calling in tumour samples.

Details regarding library preparation, sequencing platform, and processing of the internal replication cohort samples can be found in the original publication by Fernández-Rozadilla *et al*., 2021 [22].

Variant annotation was conducted using SnpEff 5.0e and ANNOVAR (version 2020Jun7).

#### RNA-seq

RNA-seq was performed on paired normal and tumour samples from the TOGETHER cohort using the TruSeq Stranded mRNA Library Prep kit (Illumina Inc.), with sequencing carried out on the Illumina HiSeq 3000 platform at the Spanish National Centre for Genomic Analysis. For each biological sample, at least 50 million paired-end 2×76 base pair reads were obtained. Sequencing specifications for TCGA and GTEx samples have been previously described elsewhere [23, 24].

Raw RNA-seq data from TOGETHER, TCGA (discovery subset), and GTEx cohorts were aligned to the GRCh37 reference genome using STAR v2.7.0e in the 2-pass mode. Gene annotation was based on GENCODE v19. Gene expression quantification was performed using RNA-SeQC v2.4.2. Expression values were normalised using trimmed mean of M values method, followed by log2 transformation using the edgeR v3.38.4 package in R. Batch effects not related to biological variability were corrected prior to downstream analysis using ComBat. Prior to CMS classification, the normalised and corrected data were represented in a principal component analysis plot using the R package PCAtools v2.8.0, to evaluate global expression patterns across normal colonic mucosa and CRC samples from individuals aged ≤65 years. Two samples (samples 204 and 307 from TOGETHER cohort) initially labelled as tumours clustered with normal tissue, and were excluded from further analysis (Supplementary Fig. 1). In addition, a chi-square test of independence was performed to assess whether the distribution of tumours across CMS differed between early- and late-onset CRC.

### Tumour molecular profiling from WES and RNA-seq data

Tumour mutational signatures were extracted using the SigProfilerAssignment tool (Python v3.8) and COSMIC v3.3 as a reference. Samples from the TCGA cohort with mutational patterns consistent with prior chemotherapy (TCGA-AZ-4684, TCGA-CM-6161, TCGA-D5-6539, TCGA-AH-6897, and TCGA-CI-6620) were excluded from further analysis, in accordance with cohort inclusion criteria. Tumours with profiles suggestive of hereditary cancer predisposition syndromes, such as those reflecting defects in the MMR system and/or in the exonuclease domain of DNA polymerases ε in the context of high TMB, were retained to support accurate CMS classification (tumours TCGA-CM-6674, TCGA-D5-6927, TCGA-F4-6856, and TCGA-EI-6917).

Tumour mutational burden (TMB) was calculated using the pyTMB v1.3.0 package (Python v3.8), following the developers’ guidelines. TMB was reported as the number of mutations per megabase, adjusted for the exome capture size used in each sample.

CMS classification was conducted on 34 MMR-proficient tumours from EOCRC patients from the discovery cohort. To reduce classification bias, RNA-seq data from an additional 60 CRC tumours from individuals aged ≤65 years (regardless of MMR status) were also included. CMS assignment was performed using the CMSCaller v0.99.2 R package, applying 1000 permutations and a false discovery rate (FDR) threshold of <0.05. The distribution of tumours was graphically represented using the R package PCAtools v2.8.0 based on normalised RNA-seq expression data.

### Multi-omic integration

#### Germline variant filtering

Germline WES data from the discovery cohort were filtered based on the following criteria: minimum coverage of 20X, variant allele frequency ≥20%, and localisation within overlapping target regions from both exome capture kits. Rare variants were prioritised based on population allele frequency threshold in non-Finnish Europeans (gnomAD v2.1.1 genome ≤0.1% for heterozygous, ≤1% for homozygous/potential compound heterozygous variants), and were required to be located in exonic or proximal intronic regions (±20 base pairs). No pathogenic or likely pathogenic variants were identified at the germline level in genes previously associated with hereditary CRC susceptibility, and the presence of mosaic variants (variant allele frequency ≥5%) in these genes was also excluded (Supplementary Table 4). All prioritised variants were individually curated using the Integrative Genomics Viewer.

#### Differential expression and variant impact analysis: ‘All vs One’ approach

A per-sample differential expression analysis was performed for each of the 34 tumours from the discovery cohort. Each tumour’s RNA expression profile was compared against normal mucosa samples from the TOGETHER and GTEx cohorts. The analysis used the edgeR v3.38.4 package, employing a quasi-likelihood framework on trimmed mean of M values-normalised and log2-transformed counts. Genes with |log2 fold change| ≥1.5 and FDR < 0.05 were retained.

For each tumour, the resulting gene list was cross-referenced with the corresponding filtered germline variant list to identify potential functional impact. Additionally, intra-CMS comparisons were made, and the presence of potential second hits in genes harbouring germline variants was explored. Only somatic variants with a variant allele frequency ≥5% were considered.

### Variant validation in the replication cohort

Germline variant filtering was performed as described in the ‘Germline Variant Filtering’ section. Due to the lack of RNA-seq data in this cohort, missense variants were selected based on their predicted functional impact, using the following in silico predictors: SIFT Deleterious (D), PolyPhen-2 Probably Damaging (D) or Possibly Damaging (P), MutationTaster Disease Causing Automatic (A) or Disease Causing (D), PROVEAN < –2.5, and CADD_pred ≥20, which together provide a high level of concordance [25]. Missense variants for which at least four predictors indicated potential functional impact were prioritised. Additionally, SpliceAI predictions were reviewed for selected variants to assess potential effects on splicing. All variants were manually inspected using the Integrative Genomics Viewer.

## Results

### Tumour molecular profiling

Both CMS and mutational signature analyses reveal molecular heterogeneity in the EOCRC discovery cohort.

**Fig. 1:**
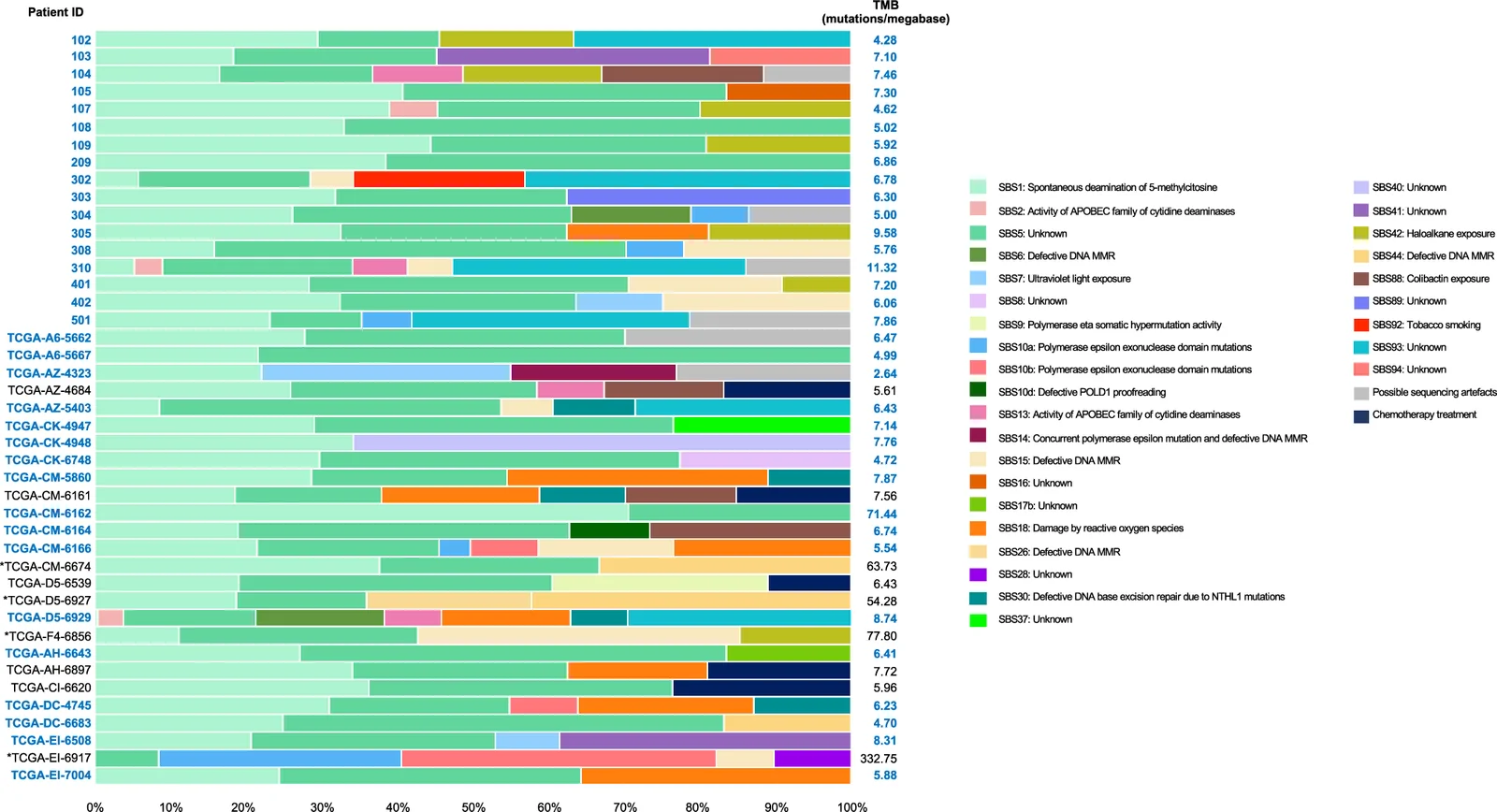
Mutational signature patterns (single nucleotide variants, COSMIC v3.3) and tumour mutational burden in EOCRC from the discovery cohort. The figure focuses on 34 EOCRC cases, highlighted in bold blue font. Mutational signature profiling revealed substantial heterogeneity across EOCRC, with frequent contributions from environmental exposure-related processes and signatures of unknown aetiology, notably the recurrent SBS93. TMB was generally low, (<10 mutations/megabase) in most tumours. Additionally, four cases displayed distinct hypermutator phenotypes (indicated with an asterisk): three with MMR deficiency-associated signatures and elevated TMB (TCGA-CM-6674, TCGA-D5-6927, and TCGA-F4-6856), and one harbouring polymerase ε exonuclease domain mutations (TCGA-EI-6917). Five tumours (TCGA-AZ-4684, TCGA-CM-6161, TCGA-D5-6539, TCGA-AH-6897, and TCGA-CI-6620) exhibited chemotherapy-associated mutational patterns and were excluded from subsequent analyses.

Mutational signature profiling showed substantial heterogeneity across colorectal tumours with frequent contributions from environmental exposure-related processes including colibactin, oxidative DNA damage, and signatures of unknown aetiology, including recurrent SBS93 (Fig. 1; Supplementary Table 5). TMB was generally low, with most tumours presenting fewer than 10 mutations/megabase (Fig. 1). Only six (17.65%) exceeded this threshold. MMR-associated signatures were detected in 17 tumours, although only three EOCRC patients from TCGA (TCGA-CM-6674, TCGA-D5-6927 and TCGA-F4-6856) showed >30% contribution and TMB above 10 mutations/megabase, consistent with MMR-deficient tumours. These three patients were excluded from the ’All vs One’ approach.

Additional signatures indicative of DNA repair deficiencies, including those related to polymerase function, APOBEC activity, and base excision repair, were also identified. These cases include patient 310 (10.94% APOBEC activity signature, TMB 11.32 mutations/megabase), patient TCGA-CM-6162 with high TMB (71.44 mutations/megabase) but no DNA repair-associated signatures, and patient TCGA-EI-6917 who was excluded from further analysis due to molecular features (72.73% signature contribution associated with mutations in the exonuclease domain of polymerase ε, TMB 332.75 mutations/megabase and the somatic hotspot p.(Val411Leu) in *POLE)*.

**Fig. 2:**
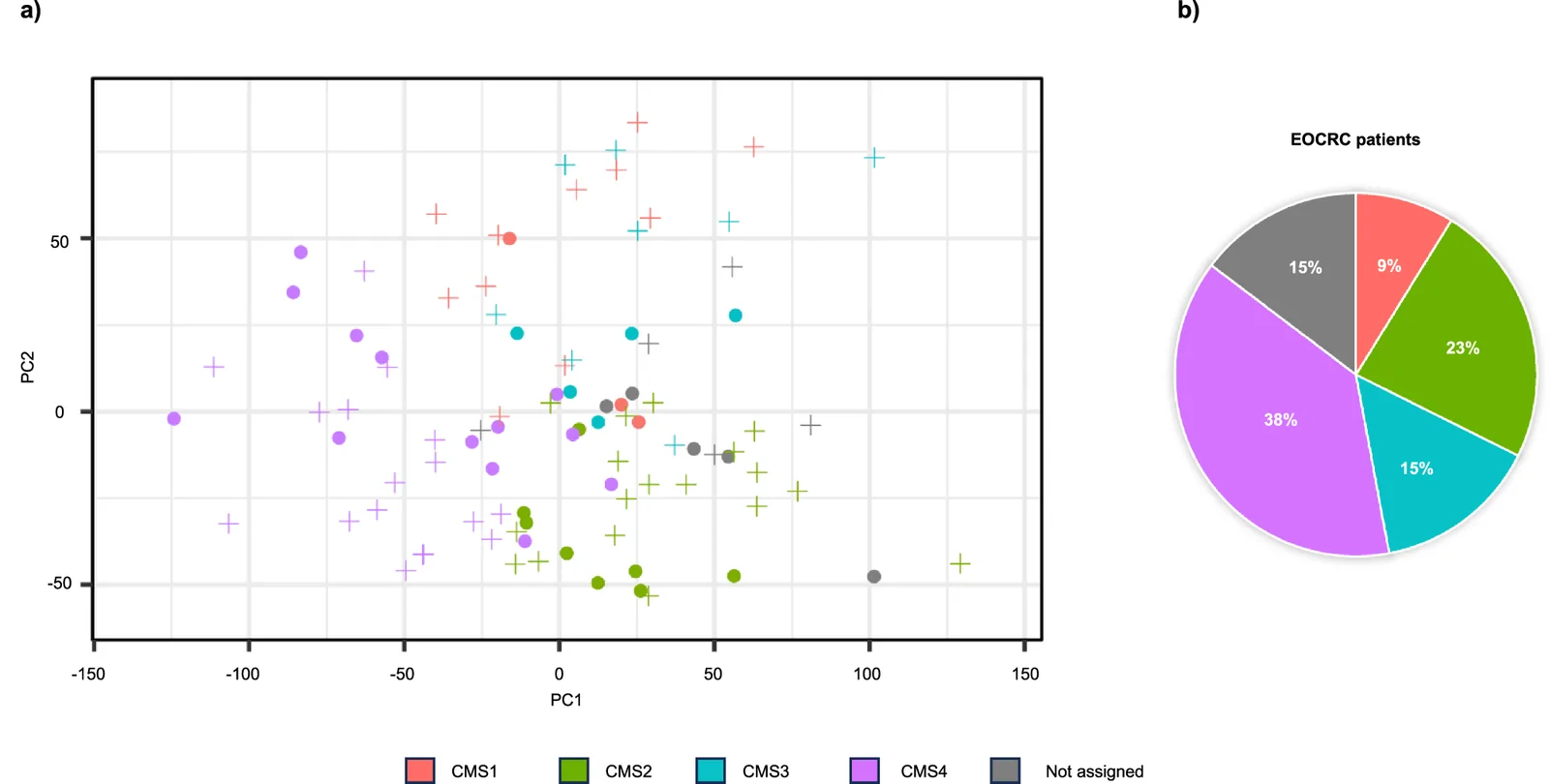
Distribution of patients according to CMS classification. **a** Principal component analysis of CRC samples based on gene expression profiles. EOCRC cases analysed in the main study (*n* = 34) are shown as coloured circles, while additional samples (CRC patients aged 51–65 years) included to improve statistical power are shown as crosses. CMS colours are shown on the principal component analysis of bulk RNA expression to illustrate subtype assignment. No significant association was detected between patient age of onset (early vs. late) and CMS distribution (chi-square test of independence, chi-square= 1.32, df= 3, *p*-value = 0.73). **b** Pie charts showing CMS distribution of EOCRC patients in the discovery cohort (*n* = 34), with colours matching the CMS in **a**.

CMS classification of CRC tumours revealed a heterogeneous distribution across the four defined molecular subtypes. Among all analysed tumours, CMS2 and CMS4 were the most prevalent (accounting for 27.96% and 32.26% of cases, respectively), while a subset of cases remained unclassified (10 tumours) (Supplementary Table 6). Principal component analysis showed that tumour samples did not form sharply distinct clusters but instead exhibited gradual separation, with partial overlap observed between CMS1 and CMS3, as well as between CMS2 and CMS4 (Fig. 2a).

Focusing on EOCRC (*n* = 34), a similarly heterogeneous distribution was observed. CMS4 was the most frequent subtype (38%), followed by CMS2 and CMS3. A minority of tumours fell into CMS1 or could not be classified into any of the defined subgroups (Fig. 2b).

### Multi-omics integration: variant filtering and ‘All vs One’ approach

Candidate genes potentially involved in colorectal tumorigenesis were identified by integrating germline and tumour exome data with gene expression data from the ‘All vs One’ strategy (see Materials and Methods; Supplementary Tables 7-40). Gene expression was also compared across individuals within the same CMS to identify genes commonly deregulated within each subtype.

In CMS3, patient TCGA-CI-6620 harboured a germline heterozygous stopgain variant in *ADCY4* p.(Arg577*) (Table 2), a gene involved in metabolic and cancer signalling pathways (KEGG hsa01100 and hsa05200), also showing significant downregulation in the tumour (log2 fold change = –2.51, FDR = 3.83×10⁻⁵).

**Table 2:** List of germline variants in candidate genes prioritised based on differential expression in tumours from EOCRC patients, after applying variant prioritisation criteria.

Gene	Germline variant	Zygosity	gnomAD v2.1.1 (%)	Patient ID	CMS	Gene function
*ADCY4*	NM_001198568.2:c.1729 C > T, p.(Arg577*)	Heterozygous	0.017	TCGA-CI-6620	3	Metabolic pathways [31]
*ARHGAP10*	NM_024605.4:c.1471 C > T, p.(Arg491Cys)	Heterozygous	0	TCGA-AZ-5403	4	PI3K/Akt and Wnt pathways modulation [37, 38]
*CDHR2*	NM_017675.6:c.3548 G > A, p.(Arg1183Gln)	Heterozygous	0.003	TCGA-AZ-4323	4	Intestinal morphology [39, 40]
*NOXO1*	NM_172167.3:c.706 C > T, p.(Gln236*)	Heterozygous	0.048	TCGA-EI-7004	4	Metabolic pathways [34, 35]
*CDHR2*	NM_017675.6:c.2824 G > A, p.(Val942Met)	Heterozygous	0	402	Not assigned	Intestinal morphology [39, 40]
*EEF2K*	NM_013302.5:c.297 G > A, p.(Trp99*)	Heterozygous	8.81×10-4	501	Not assigned	Elongation factor [41–43]

*CMS* consensus molecular subtype.

In CMS4, three patients carried germline variants with transcriptomic and functional features potentially compatible with colorectal tumorigenesis (Table 2). In TCGA-AZ-4323, a missense variant in *CDHR2* p.(Arg1183Gln) was detected (log2 fold change = –2.84, FDR = 4.18×10⁻²). In TCGA-AZ-5403, a missense variant in *ARHGAP10* p.(Arg491Cys), located in the Rho GTPase activation domain (log2 fold change = –2.46, FDR = 4.98×10⁻²) was identified. In TCGA-EI-7004, a stopgain variant was found in *NOXO1* p.(Gln236*) (log2 fold change = –1.92, FDR = 4.48×10⁻²).

Among tumours not assigned to a CMS subgroup, two individuals presented rare germline variants in genes with altered expression in tumour tissue (Table 2). Patient 402 carried a heterozygous missense variant in *CDHR2* p.(Val942Met) (log2 fold change = –2.92, FDR = 4.12×10⁻²). Patient 501 had a monoallelic stopgain variant in *EEF2K*, p.(Trp99*) (log2 fold change = –1.53, FDR = 3.20×10⁻²) and evidence of nonsense-mediated decay in both germline and tumour RNA, visualised using Integrative Genomics Viewer.

No candidate genes were identified for the CMS1 and CMS2 subgroups, and no secondary somatic mutations were detected in the coding regions of the candidate genes across any subgroup, considering only SNVs and indels.

### Replication cohort

To validate the findings from the discovery cohort, germline WES data from the replication cohort were analysed to identify rare, potentially deleterious variants in the five candidate genes. The samples were first screened for pathogenic germline variants in the known cancer predisposition genes (Supplementary Table 4). A heterozygous stopgain variant in *NTHL1* (NM_002528.7:c.835 C > T, p.(Gln279*)) was identified in patient TCGA-DC-6155. No additional (likely) pathogenic variants were detected.

For the candidate genes, heterozygous germline missense variants in *EEF2K* were identified in two unrelated patients (Table 3). In both cases, in silico predictions supported a potential impact on protein function. Variant p.(Ala164Thr) is located within the MHCK/EF2 kinase, Protein kinase-like, and Elongation factor 2 kinase domains. Variant p.(Pro686Leu) maps to the Sel1-like and Elongation factor 2 kinase domains.

**Table 3:** Rare germline variants identified in candidate genes in the replication cohort.

Gene	Germline variant	Zygosity	gnomAD v2.1.1 (%)	Patient ID	SIFT	PolyPhen-2	MutationTaster	PROVEAN	CADD
*EEF2K*	NM_013302.5:c.490 G > A, p.(Ala164Thr)	Heterozygous	0	TCGA-DM-A1D8	D	D	D	-2.69	24.4
*EEF2K*	NM_013302.5:c.2057 C > T, p.(Pro686Leu)	Heterozygous	7.19×10-5	TCGA-D5-6541	T	P	D	-4.32	23.4

Prioritised variants were identified in two unrelated patients based on variant filtering criteria. In silico predictions for the EEF2K variants are shown. *SIFT: T* tolerated, *D* deleterious. *PolyPhen-2: D* probably damaging, *P* possibly damaging. *MutationTaster: D* disease causing.

## Discussion

CRC is a common disease arising from complex interactions between genetic and environmental factors. In recent years, its incidence among individuals under 50 years of age has increased by 1.4% annually [26]. By 2030, it is estimated that 11% of colon cancers and 23% of rectal cancers will be diagnosed in this age group. Current evidence suggests that 16–35% of these tumours may be attributable to germline pathogenic variants in genes associated with known Mendelian cancer predisposition syndromes or moderate-risk CRC susceptibility genes [27, 28]. EOCRC is increasingly recognised as a distinct and heterogeneous entity compared to late-onset CRC, exhibiting differences in mutational burden, driver mutations, gene expression, and other molecular profiles [29–31].

In our cohort, tumours were classified according to the CMS [14], with 9% CMS1 (MSI-immune), 23% CMS2 (canonical), 15% CMS3 (metabolic), 38% CMS4 (mesenchymal), and 15% unclassified. Comparison of CMS distribution with late-onset cohort (51–65 years) showed no significant segregation by age (*p*-value = 0.73).

Mutational signature analysis revealed patterns linked to age and mitotic activity (SBS1, SBS5, SBS40), exogenous exposures and oxidative damage (e.g., SBS18, SBS42, SBS88, SBS92), and low-percentage signatures associated with DNA repair defects (e.g., SBS6, SBS10a/b/d, SBS14, SBS30) [32]. Several signatures of unknown aetiology were also observed (16–43%) further highlighting the complexity and heterogeneity of EOCRC. Despite the absence of elevated TMB and identifiable (likely) pathogenic variants in these pathways, the presence of these signatures may indicate indirect impairment of DNA repair systems or uncharacterised mutational processes. While informative, mutational signatures must be interpreted cautiously due to their probabilistic nature, potential overlaps, and limitations of WES. Future studies incorporating whole-genome sequencing and organ-specific reference datasets will be crucial for refining molecular classification and elucidating unexplained mutational patterns.

In light of the molecular heterogeneity and the limited detection of germline variants, we applied an integrative, patient-centred ‘All vs One’ approach combining germline and somatic WES with transcriptomic data to identify candidate hereditary CRC susceptibility genes. This strategy prioritised variants with functional impact at the individual patient level, reflecting the biological diversity of EOCRC and addressing the challenges reported in previous studies, which failed to identify specific germline variants in MMR-proficient EOCRC [27, 33].

Several studies, often incorporating TCGA samples, have addressed related aspects of EOCRC biology, including integrative germline-somatic analyses to assess potential second-hit [34], CMS classification [30, 31], and multi-omic tumour profiling approaches [35]. Other works have developed computational strategies to enable single-sample differential expression analysis in CRC and other cancer types [36, 37]. These contributions have advanced our understanding of somatic alterations, CMS stratification, and tumour heterogeneity. However, to our knowledge, no previous study has combined EOCRC enrichment with mutational signature profiling, CMS classification, per sample tumour-to-pooled normal RNA-seq differential expression (our ‘All vs One’ approach), systematic cross-referencing with germline variants, and second-hit discovery within a single integration workflow.

Using our novel ‘All vs One’ strategy, we identify five candidate genes potentially implicated in tumorigenesis through various biological pathways: *ADCY4*, *NOXO1, CDHR2*, *ARHGAP10* and *EEF2K*.

*ADCY4*, which encodes adenylate cyclase 4, is involved in cAMP production and regulates key cellular processes including proliferation, apoptosis, and cellular metabolism [38]. A germline stopgain variant was identified in one patient, whose tumour exhibited significantly reduced gene expression. Loss-of-function variants in this gene have been previously reported as a pathogenic mechanism, and *ADCY4* has been associated with tumour suppression in breast cancer [39, 40]. This tumour was classified within the CMS3 subtype, which is characterised by metabolic dysregulation, consistent with the gene’s function. Altogether, these findings support a potential role for *ADCY4* in colorectal tumorigenesis. Similarly, *NOXO1* is another gene involved in cellular metabolism, though acting within a distinct biological context. *NOXO1*, a regulator of NADPH oxidase activity, contributes to intestinal epithelial homoeostasis and redox balance [41]. Conflicting roles have been reported for this gene in colorectal tumorigenesis. While some studies indicate that *NOXO1* may inhibit CRC progression, other evidence links its inactivation to increase proliferation, reduced apoptosis, and chronic inflammation [41, 42]. In our study, we identified a germline loss-of-function variant in *NOXO1*, accompanied by reduced tumoral gene expression. The tumour also exhibited mutational signature SBS18, associated with oxidative DNA damage. Furthermore, redox imbalance has been implicated in the epithelial-mesenchymal transition [43], the hallmark of the CMS4 in which this tumour was classified, further supporting the role of *NOXO1* variant in the development of CRC in this patient.

Two additional candidate genes identified within the CMS4 subtype, *ARHGAP10* and *CDHR2*, are involved in the regulation of epithelial-mesenchymal transition, supporting their potential contribution to colorectal tumorigenesis in these patients. One patient carried a germline missense variant in *ARHGAP10*, a regulator of Rho GTPase signalling. This gene showed reduced expression in the tumour and has been proposed as a tumour suppressor, as it has been observed to inhibit cell proliferation and metastasis [44, 45]. In ovarian cancer, *ARHGAP10* has also been associated with the regulation of DNA replication, cell cycle and base excision repair. Interestingly, in the present case, the tumour exhibited mutational signatures indicative of defects in MMR (6.86%) and base excision repair (10.86%) pathways.

Another patient in the CMS4 harboured a missense germline variant in *CDHR2*, accompanied by significantly reduced tumour expression. *CDHR2* encodes a protocadherin involved in intestinal cell adhesion as well as epithelial structure and function, and is also implicated in the regulation of Wnt signalling [46, 47]. A second patient, not assigned to any CMS, carried another missense variant in *CDHR2* and showed similar expression decrease, supporting a potential role for *CDHR2* disruption in CRC development.

Finally, one patient not assigned to any CMS subtype harboured a truncating germline variant in *EEF2K*, an atypical kinase regulating protein synthesis and reported to suppress CRC development [48–50]. Transcript degradation was consistent with nonsense-mediated decay. Additionally, two patients in the replication cohort carried missense germline variants in this gene, both predicted by in silico tools to affect protein function.

Among the 34 EOCRC patients analysed, we propose a candidate predisposition gene in six cases. In the remaining 28, no candidate germline variants were identified in coding regions. For these individuals, sporadic disease driven by environmental factors cannot be excluded, particularly given the presence of exposure-related mutational signatures in some cases. However, strong familial aggregation of CRC was observed in some patients, suggesting an underlying hereditary component may still be involved. The contribution of copy number variants, structural rearrangements, low-penetrance alleles, or regulatory variants not assessed in our study remains a possibility.

This study underscores the molecular heterogeneity of early-onset CRC, even within a clinically homogeneous cohort. The use of integrated multi-omic profiling allowed for prioritisation of candidate susceptibility genes with functional impact, highlighting the value of transcriptomic data in refining the interpretation of germline findings. Therefore, public repositories incorporating paired tumour and normal tissue omics data will be essential for future gene discovery efforts in hereditary CRC genomics.

## Supplementary information

Supplementary Material

## Data Availability

The datasets generated during and/or analysed during the current study are available in the European Genome-phenome Archive (EGA) under Study ID EGAS50000001296. In addition, the study includes some data obtained from dbGaP, which were used under license and are subject to restrictions; therefore, they are not publicly available.
