# Supplementary material for: Integration of multi-omics data uncovers novel germline susceptibility candidates in early-onset colorectal cancer
Source: Eur J Hum Genet. 2026 Jan 15;34(9):1327–35. doi: 10.1038/s41431-025-02004-7 (PMC13550470; doi:10.1038/s41431-025-02004-7)
Supplement: Supplementary file 1 [file 41431_2025_2004_MOESM1_ESM.pdf]

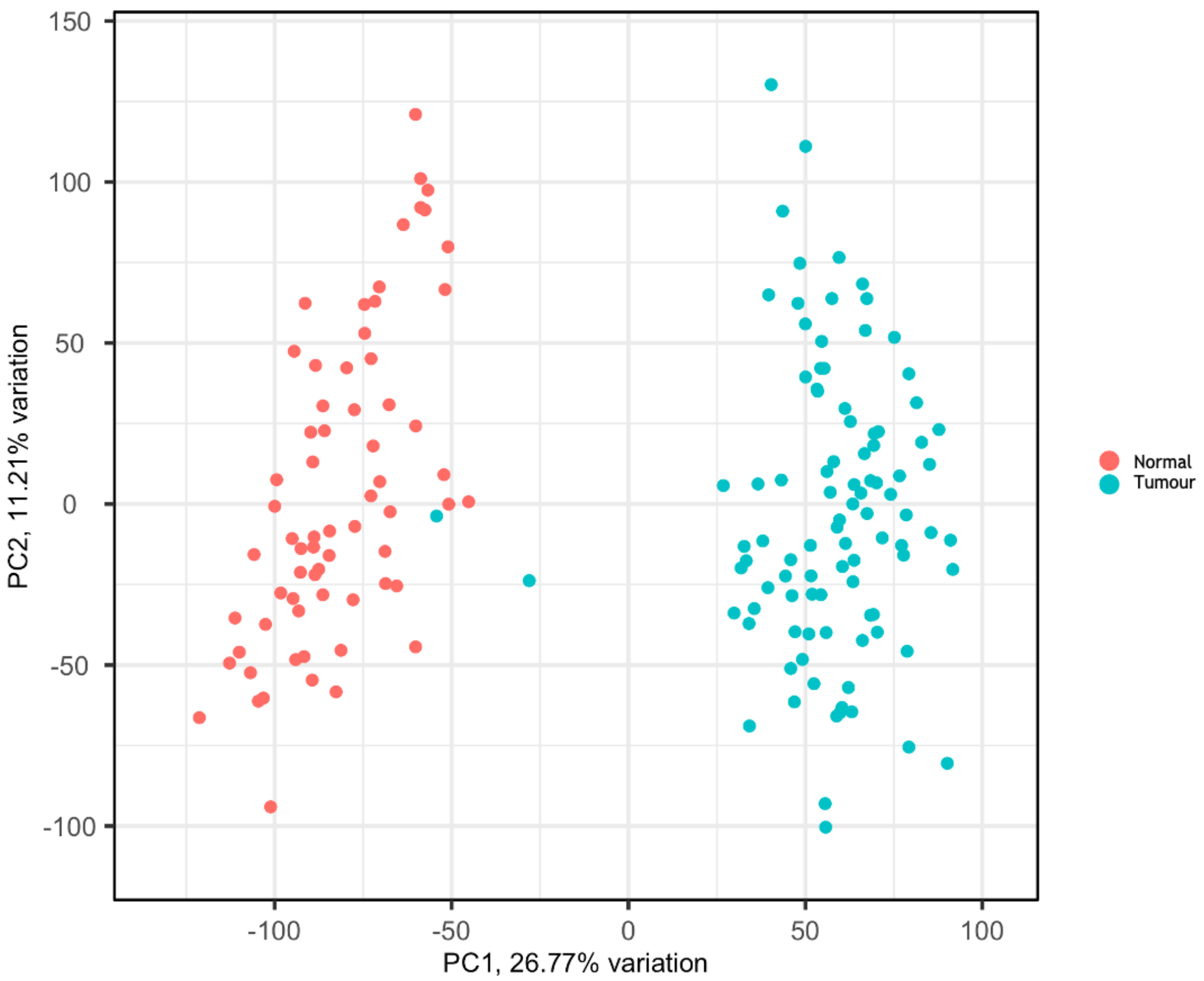

Supplementary Figure 1. Principal component analysis of normal colonic mucosa and CRC samples based on gene expression profiles. Principal component analysis plot showing the distribution and clustering of normal colonic mucosa and CRC samples according to their gene expression patterns. Notably, tumour samples 204 and 307 clustered together with the normal mucosa samples, suggesting a similar expression profile.

Supplementary Table 1. Clinical data of TCGA patients included in CMS classification analysis. RNA-seq data from 55 CRC patients aged between 51 and 65 years, obtained from COAD and READ cohorts, were used for CMS classification.

Patient ID	CRC diagnosis age	Sex	Tumour site	Tumour stage
TCGA-A6-6137	55	Male	Hepatic flexure	III
TCGA-CM-6678	63	Female	Sigmoid colon	IV
TCGA-A6-6651	55	Female	Transverse colon	III
TCGA-CK-5913	58	Female	Cecum	I
TCGA-D5-5541	63	Male	Sigmoid colon	III
TCGA-CM-6679	58	Male	Sigmoid colon	II
TCGA-D5-6534	62	Female	Ascending colon	II
TCGA-D5-6532	61	Male	Sigmoid colon	II
TCGA-D5-6537	64	Male	Transverse colon	III
TCGA-A6-6142	56	Female	Sigmoid colon	IV
TCGA-AD-6964	58	Male	Cecum	Not specified
TCGA-A6-6654	65	Female	Descending colon	III
TCGA-F4-6459	61	Female	Sigmoid colon	III
TCGA-A6-6648	56	Male	Not specified	IV
TCGA-G4-6297	55	Female	Cecum	IV
TCGA-G4-6588	58	Female	Cecum	II
TCGA-A6-6138	61	Male	Cecum	I
TCGA-AD-6965	62	Male	Cecum	III
TCGA-CM-5864	60	Male	Cecum	I
TCGA-F4-6809	52	Female	Sigmoid colon	IV
TCGA-A6-6140	62	Male	Descending colon	II
TCGA-F4-6806	59	Female	Sigmoid colon	I
TCGA-G4-6317-1	51	Female	Sigmoid colon	III
TCGA-CM-5868	59	Female	Sigmoid colon	IV
TCGA-CM-5861	63	Female	Cecum	II
TCGA-AZ-5407	51	Female	Cecum	I
TCGA-F4-6805	58	Female	Descending colon	II
TCGA-AZ-4682	61	Male	Sigmoid colon	IV
TCGA-D5-6898	51	Female	Sigmoid colon	I
TCGA-AZ-4313	51	Female	Descending colon	I
TCGA-F4-6807	51	Female	Hepatic flexure	III
TCGA-CM-4751	62	Male	Cecum	III
TCGA-AY-6197	60	Male	Cecum	II
TCGA-F4-6463	51	Male	Transverse colon	II
TCGA-AZ-4315	61	Male	Cecum	II
TCGA-D5-5538	60	Male	Cecum	III
TCGA-G4-6303	54	Female	Sigmoid colon	IV
TCGA-D5-6923	57	Male	Sigmoid colon	I
TCGA-G4-6322	65	Male	Descending colon	III

TCGA-D5-5539	60	Male	Ascending colon	III
TCGA-EI-6513	59	Male	Rectosigmoid junction	III
TCGA-DC-4749	57	Male	Rectosigmoid junction	I
TCGA-F5-6814	57	Male	Rectum	II
TCGA-DC-6156	62	Male	Rectosigmoid junction	IV
TCGA-DC-6154	57	Female	Rectosigmoid junction	IV
TCGA-F5-6465	64	Female	Rectosigmoid junction	II
TCGA-EI-6507	60	Male	Rectum	II
TCGA-DC-6682	57	Male	Rectosigmoid junction	II
TCGA-EF-5830	54	Male	Rectum	II
TCGA-EI-6512	64	Female	Rectosigmoid junction	III
TCGA-EI-7002	58	Male	Rectum	IV
TCGA-EI-6882	59	Male	Rectosigmoid junction	II
TCGA-EI-6885	57	Female	Rectosigmoid junction	IV
TCGA-F5-6861	60	Female	Rectum	II
TCGA-EI-6883	63	Male	Rectosigmoid junction	II

Supplementary Table 2. Characteristics of GTEx individuals used as reference for normal colon tissue. Gene expression data from GTEx subjects were used as a reference for normal colonic tissue, alongside normal colonic mucosa from TOGETHER cohort individuals. This table includes age, sex, and tissue sample location from 47 healthy GTEx donors.

Subject ID	Age	Sex	Sample site
SM-3NM9L	33	Female	Transverse colon
SM-3P5ZQ	36	Male	Transverse colon
SM-3P613	50	Male	Transverse colon
SM-48FDC	43	Female	Transverse colon
SM-48TD4	22	Female	Transverse colon
SM-48TDF	48	Female	Transverse colon
SM-48TZY	44	Female	Transverse colon
SM-48U1F	44	Male	Transverse colon
SM-48U1J	27	Female	Transverse colon
SM-4AD4Z	41	Female	Transverse colon
SM-4AD5E	22	Male	Transverse colon
SM-4AT5B	22	Female	Transverse colon
SM-4AT67	50	Male	Transverse colon
SM-4B64Z	44	Male	Transverse colon
SM-4BRV2	31	Female	Transverse colon
SM-4BRWU	45	Female	Transverse colon
SM-4DM5V	27	Female	Transverse colon
SM-4DXT9	50	Male	Transverse colon
SM-4DXU1	33	Female	Transverse colon
SM-4DXV3	47	Male	Transverse colon
SM-4LVMG	25	Male	Transverse colon
SM-4LVMS	49	Male	Transverse colon
SM-4LVMT	43	Male	Transverse colon
SM-4M1XJ	50	Male	Transverse colon
SM-4OND6	46	Female	Transverse colon
SM-4OOSQ	24	Male	Transverse colon
SM-4R1KR	32	Male	Transverse colon
SM-4VDTA	42	Female	Transverse colon
SM-4WWCM	29	Female	Transverse colon
SM-4WWDN	46	Male	Transverse colon
SM-57WDX	48	Female	Transverse colon
SM-5985O	47	Male	Transverse colon
SM-5986U	27	Male	Transverse colon
SM-5A5KN	21	Female	Transverse colon
SM-5BC5J	21	Male	Transverse colon
SM-5EGGC	23	Male	Transverse colon
SM-5EQKC	47	Female	Transverse colon
SM-5IFEN	26	Female	Transverse colon
SM-5IFFO	50	Male	Transverse colon
SM-5IFIQ	49	Male	Transverse colon
SM-5IJEN	44	Male	Transverse colon
SM-5J2M5	33	Female	Transverse colon
SM-5LU52	22	Male	Transverse colon
SM-5LU9C	44	Male	Transverse colon
SM-5NQBY	37	Male	Transverse colon

SM-5Q5C5	44	Male	Transverse colon
SM-5RQIX	50	Male	Transverse colon

Supplementary Table 3. Clinical characteristics of patients from validation cohort.

FDR: first-degree relative; SDR: second-degree relative

Patient ID	CRC diagnosis age (range)	Sex	Family cancer history
TCGA-A6-2678	40-44	Female	Yes – type not specified
TCGA-AY-6196	45-50	Male	Yes – type not specified
TCGA-CK-4952	45-50	Female	Unknown
TCGA-CM-4747	45-50	Male	None
TCGA-CM-4750	<40	Female	None
TCGA-D5-6541	45-50	Male	Unknown
TCGA-DM-A1D8	45-50	Female	None
TCGA-DM-A28H	45-50	Male	None
TCGA-F4-6461	40-44	Female	Unknown
TCGA-G4-6293	45-50	Female	Yes – type not specified
TCGA-G4-6307	<40	Female	None
TCGA-AF-2689	40-44	Female	Unknown
TCGA-AF-2691	45-50	Female	None
TCGA-AF-3911	45-50	Male	None
TCGA-AF-6672	40-44	Male	None
TCGA-AH-6903	45-50	Male	None
TCGA-CI-6619	40-44	Male	Unknown
TCGA-CI-6623	40-44	Male	Unknown
TCGA-DC-6155	<40	Female	Yes – type not specified
PC-001	40-44	Female	None
PC-002	40-44	Male	None
PC-003	45-50	Male	None
PC-004	<40	Female	None
PC-005	<40	Male	None
PC-006	45-50	Male	FDR – multiple cancers (colorectal, leukemia)
PC-008	45-50	Male	None
PC-009	45-50	Female	None
PC-010	40-44	Female	None
PC-011	<40	Male	None
PC-012	45-50	Male	None
PC-013	45-50	Female	None
PC-014	45-50	Male	FDR & SDR – multiple cancers
PC-015	<40	Female	None
PC-016	45-50	Male	SDR - colorectal
PC-017	40-44	Male	None
PC-018	45-50	Male	None
PC-019	40-44	Female	None
PC-020	45-50	Male	None
PC-021	<40	Female	None

Supplementary Table 4. Genes associated with hereditary colorectal cancer susceptibility. AD: autosomal dominant inheritance. AR: autosomal recessive inheritance.

Gene	Syndrome	Inheritance
MLH1	Lynch syndrome	AD
	Constitutional Mismatch Repair Deficiency syndrome	AR
MSH2	Lynch syndrome	AD
	Constitutional Mismatch Repair Deficiency syndrome	AR
MSH6	Lynch syndrome	AD
	Constitutional Mismatch Repair Deficiency syndrome	AR
PMS2	Lynch syndrome	AD
	Constitutional Mismatch Repair Deficiency syndrome	AR
EPCAM	Lynch syndrome	AD
	Constitutional Mismatch Repair Deficiency syndrome	AR
APC	Adenomatous polyposis coli	AD
AXIN2	Oligodontia-colorectal cancer syndrome	AD
MUTYH	MUTYH-associated polyposis	AR
NTHL1	NTHL1-associated polyposis	AR
POLE	Polymerase proofreading-associated polyposis	AD
POLD1	Polymerase proofreading-associated polyposis	AD
GREM1	Hereditary mixed polyposis syndrome	AD
MSH3	MSH3-associated polyposis	AR
STK11	Peutz-Jeghers syndrome	AD
SMAD4	Juvenile polyposis	AD
BMPR1A	Juvenile polyposis	AD
RNF43	Sessile serrated polyposis cancer syndrome	AD
PTEN	PTEN hamartoma tumour syndrome	AD
TP53	Li-Fraumeni syndrome	AD
MBD4	MBD4-associated neoplasia syndrome	AR
RPS20	RPS20-associated nonpolyposis colorectal cancer	AD

Supplementary Table 5. Percentage contribution of mutational signatures in EOCRC patients from the TOGETHER and TCGA cohorts. Mutational signature profiles were obtained using SigProfilerAssignment, based on COSMIC v3.3 reference signatures. The table shows the relative contribution (in %) of each mutational signature in individual tumours from EOCRC patients included in the TOGETHER and TCGA (discovery subset) cohorts.

		**Patient ID**								
		**102**	**103**	**104**	**105**	**107**	**108**	**109**	**209**	**302**
**SBS signatures**	SBS1	29.44%	18.28%	16.48%	40.66%	38.95%	32.91%	44.39%	38.46%	5.76%
	SBS2					6.32%				
	SBS5	16.11%	26.88%	20.22%	42.86%	34.74%	67.09%	36.45%	61.54%	22.66%
	SBS6									
	SBS7a									
	SBS7b									
	SBS7d									
	SBS8									
	SBS9									
	SBS10a									
	SBS10b									
	SBS10d									
	SBS13			11.99%						
	SBS14									
	SBS15									5.76%
	SBS16				16.48%					
	SBS17b									
	SBS18									
	SBS26									
	SBS28									

	Patient ID								
SBS signatures	102	103	104	105	107	108	109	209	302
SBS30									
SBS37									
SBS40									
SBS41		36.20%							
SBS42	17.78%		18.35%		20.00%		19.16%		
SBS44									
SBS88			21.35%						
SBS89									
SBS92									22.66%
SBS93	36.67%								43.17%
SBS94		18.64%							
Possible sequencing artefacts			11.61%						
Chemotherapy treatment									

	Patient ID								
**SBS signatures**	**303**	**304**	**305**	**308**	**310**	**401**	**402**	**501**	**TCGA-A6-5662**
SBS1	31.78%	26.09%	32.47%	15.77%	5.22%	28.29%	32.37%	23.16%	27.74%
SBS2					3.73%				
SBS5	30.62%	36.96%	29.89%	54.50%	25.12%	42.23%	31.21%	12.13%	42.34%
SBS6		15.76%							
SBS7a							11.56%		
SBS7b									
SBS7d									
SBS8									
SBS9									
SBS10a		7.61%		7.66%				6.62%	
SBS10b									
SBS10d									
SBS13					7.21%				
SBS14									
SBS15				22.07%	5.97%	20.32%	24.86%		
SBS16									
SBS17b									
SBS18			18.82%						
SBS26									
SBS28									
SBS30									
SBS37									
SBS40									

		Patient ID								
		**303**	**304**	**305**	**308**	**310**	**401**	**402**	**501**	**TCGA-A6-5662**
**SBS signatures**	SBS41									
	SBS42			18.82%			9.16%			
	SBS44									
	SBS88									
	SBS89	37.60%								
	SBS92									
	SBS93					38.81%			36.76%	
	SBS94									
	Possible sequencing artefacts		13.59%			13.93%			21.32%	29.93%
	Chemotherapy treatment									

SBS signatures	Patient ID								
	TCGA-A6-5667	TCGA-AZ-4323	TCGA-AZ-4684	TCGA-AZ-5403	TCGA-CK-4947	TCGA-CK-4948	TCGA-CK-6748	TCGA-CM-5860	TCGA-CM-6161
SBS1	21.53%	21.98%	25.79%	8.57%	29.00%	34.18%	29.69%	28.63%	18.48%
SBS2									
SBS5	78.47%		32.63%	45.14%	47.50%		47.66%	25.88%	19.43%
SBS6									
SBS7a		9.89%							
SBS7b									
SBS7d		23.08%							
SBS8							22.66%		
SBS9									
SBS10a									
SBS10b									
SBS10d									
SBS13			8.95%						
SBS14		21.98%							
SBS15				6.86%					
SBS16									
SBS17b									
SBS18								34.51%	20.85%
SBS26									
SBS28									
SBS30				10.86%				10.98%	11.37%
SBS37					23.50%				
SBS40						65.82%			

		Patient ID								
		**TCGA-A6-5667**	**TCGA-AZ-4323**	**TCGA-AZ-4684**	**TCGA-AZ-5403**	**TCGA-CK-4947**	**TCGA-CK-4948**	**TCGA-CK-6748**	**TCGA-CM-5860**	**TCGA-CM-6161**
**SBS signatures**	SBS41									
	SBS42									
	SBS44									
	SBS88			15.79%						14.69%
	SBS89									
	SBS92									
	SBS93				28.57%					
	SBS94									
	Possible sequencing artefacts		23.08%							
	Chemotherapy treatment			16.84%						15.17%

	Patient ID								
**SBS signatures**	**TCGA-CM-6162**	**TCGA-CM-6164**	**TCGA-CM-6166**	**TCGA-CM-6674**	**TCGA-D5-6539**	**TCGA-D5-6927**	**TCGA-D5-6929**	**TCGA-F4-6856**	**TCGA-AH-6643**
SBS1	70.55%	18.89%	21.38%	37.62%	19.01%	18.68%	0.38%	11.12%	27.11%
SBS2							3.41%		
SBS5	29.45%	43.89%	24.14%	29.07%	41.44%	17.20%	17.42%	31.58%	56.44%
SBS6							17.05%		
SBS7a									
SBS7b									
SBS7d									
SBS8									
SBS9					28.52%				
SBS10a			4.14%						
SBS10b			8.97%						
SBS10d		10.56%							
SBS13							7.58%		
SBS14									
SBS15			17.93%					42.59%	
SBS16									
SBS17b									16.44%
SBS18			23.45%				17.05%		
SBS26						21.87%			
SBS28									
SBS30							7.58%		
SBS37									
SBS40									

		Patient ID								
		TCGA-CM-6162	TCGA-CM-6164	TCGA-CM-6166	TCGA-CM-6674	TCGA-D5-6539	TCGA-D5-6927	TCGA-D5-6929	TCGA-F4-6856	TCGA-AH-6643
SBS signatures	SBS41									
	SBS42								14.72%	
	SBS44				33.31%		42.25%			
	SBS88		26.67%							
	SBS89									
	SBS92									
	SBS93							29.55%		
	SBS94									
	Possible sequencing artefacts									
	Chemotherapy treatment					11.03%				

		Patient ID						
		TCGA-AH-6897	TCGA-CI-6620	TCGA-DC-4745	TCGA-DC-6683	TCGA-EI-6508	TCGA-EI-6917	TCGA-EI-7004
SBS signatures	SBS1	34.05%	36.22%	30.97%	24.83%	20.63%		24.32%
	SBS2							
	SBS5	28.45%	40.16%	23.87%	58.39%	32.29%	8.46%	40.00%
	SBS6							
	SBS7a							
	SBS7b					8.52%		
	SBS7d							
	SBS8							
	SBS9							
	SBS10a						32.05%	
	SBS10b			9.03%			41.67%	
	SBS10d							
	SBS13							
	SBS14							
	SBS15						7.58%	
	SBS16							
	SBS17b							
	SBS18	18.53%		23.23%				35.68%
	SBS26							
	SBS28						10.25%	
	SBS30			12.90%				
	SBS37							
	SBS40							

		Patient ID						
		TCGA-AH-6897	TCGA-CI-6620	TCGA-DC-4745	TCGA-DC-6683	TCGA-EI-6508	TCGA-EI-6917	TCGA-EI-7004
SBS signatures	SBS41					38.57%		
	SBS42							
	SBS44				16.78%			
	SBS88							
	SBS89							
	SBS92							
	SBS93							
	SBS94							
	Possible sequencing artefacts							
	Chemotherapy treatment	18.97%	23.62%					

Supplementary Table 6. CMS classification of CRC from the TOGETHER and TCGA cohorts using CMSCaller v0.99.2. Tumours from the TOGETHER and TCGA cohorts were classified into CMS using RNA-seq data and the R package CMSCalle v0.99.2. The table includes the final CMS prediction for each tumour, prediction distances to each of the four CMS, as well as the corresponding p-values and false discovery rates (FDR).

Patient ID	CMS prediction	CMS1 distance	CMS2 distance	CMS3 distance	CMS4 distance	p-value	FDR
102	CMS4	0.723	0.667	0.704	0.62	0.001	0.001
103	Not assigned	0.721	0.687	0.761	0.788	0.326	0.333
104	CMS3	0.776	0.679	0.631	0.823	0.001	0.001
105	CMS1	0.651	0.729	0.714	0.801	0.001	0.001
107	CMS3	0.723	0.681	0.604	0.832	0.001	0.001
108	CMS3	0.738	0.726	0.642	0.856	0.001	0.001
109	Not assigned	0.735	0.677	0.735	0.8	0.105	0.112
209	CMS4	0.726	0.706	0.699	0.398	0.001	0.001
302	CMS4	0.668	0.712	0.678	0.548	0.001	0.001
303	CMS2	0.677	0.64	0.658	0.708	0.001	0.001
304	CMS4	0.561	0.769	0.677	0.49	0.001	0.001
305	CMS4	0.739	0.665	0.713	0.535	0.001	0.001
308	CMS4	0.693	0.607	0.632	0.583	0.001	0.001
310	CMS2	0.765	0.662	0.814	0.69	0.005	0.006
401	CMS1	0.585	0.821	0.656	0.733	0.001	0.001
402	Not assigned	0.743	0.698	0.725	0.775	0.253	0.261
501	Not assigned	0.801	0.674	0.791	0.865	0.109	0.115
TCGA-A6-5662	CMS2	0.795	0.657	0.77	0.701	0.004	0.005
TCGA-A6-5667	CMS2	0.786	0.594	0.775	0.652	0.001	0.001
TCGA-AZ-4323	CMS4	0.623	0.739	0.72	0.469	0.001	0.001
TCGA-AZ-5403	CMS4	0.788	0.651	0.703	0.612	0.001	0.001
TCGA-CK-4947	CMS3	0.702	0.685	0.659	0.755	0.004	0.005
TCGA-CK-4948	CMS4	0.652	0.662	0.735	0.586	0.001	0.001
TCGA-CK-6748	CMS4	0.743	0.65	0.726	0.443	0.001	0.001
TCGA-CM-5860	CMS4	0.666	0.72	0.759	0.574	0.001	0.001
TCGA-CM-6162	CMS4	0.597	0.771	0.613	0.421	0.001	0.001
TCGA-CM-6164	CMS2	0.781	0.574	0.781	0.711	0.001	0.001
TCGA-CM-6166	CMS2	0.837	0.622	0.781	0.771	0.001	0.001
TCGA-CM-6674	CMS1	0.551	0.851	0.628	0.72	0.001	0.001
TCGA-D5-6927	CMS1	0.551	0.794	0.682	0.699	0.001	0.001

TCGA-D5-6929	CMS2	0.793	0.592	0.776	0.633	0.001	0.001
TCGA-F4-6856	CMS3	0.624	0.823	0.556	0.834	0.001	0.001
TCGA-A6-6137	CMS2	0.77	0.593	0.633	0.846	0.001	0.001
TCGA-CM-6678	CMS3	0.766	0.64	0.612	0.743	0.001	0.001
TCGA-A6-6651	CMS4	0.692	0.687	0.781	0.366	0.001	0.001
TCGA-CK-5913	CMS1	0.519	0.825	0.665	0.751	0.001	0.001
TCGA-D5-5541	CMS2	0.78	0.626	0.811	0.703	0.001	0.001
TCGA-CM-6679	CMS4	0.81	0.64	0.8	0.501	0.001	0.001
TCGA-D5-6534	CMS4	0.6	0.761	0.726	0.41	0.001	0.001
TCGA-D5-6532	CMS2	0.816	0.651	0.743	0.883	0.003	0.004
TCGA-D5-6537	Not assigned	0.705	0.664	0.732	0.868	0.067	0.073
TCGA-A6-6142	CMS4	0.686	0.649	0.744	0.418	0.001	0.001
TCGA-AD-6964	CMS4	0.626	0.827	0.69	0.606	0.001	0.001
TCGA-A6-6654	CMS4	0.631	0.731	0.745	0.371	0.001	0.001
TCGA-F4-6459	CMS4	0.738	0.642	0.697	0.428	0.001	0.001
TCGA-A6-6648	CMS2	0.794	0.66	0.675	0.854	0.018	0.02
TCGA-G4-6297	CMS4	0.645	0.688	0.737	0.421	0.001	0.001
TCGA-G4-6588	CMS1	0.529	0.852	0.68	0.807	0.001	0.001
TCGA-A6-6138	Not assigned	0.696	0.702	0.794	0.692	0.153	0.16
TCGA-AD-6965	CMS2	0.751	0.633	0.703	0.799	0.002	0.003
TCGA-CM-5864	CMS2	0.802	0.651	0.721	0.882	0.002	0.003
TCGA-F4-6809	CMS4	0.697	0.69	0.705	0.475	0.001	0.001
TCGA-A6-6140	CMS2	0.804	0.594	0.724	0.866	0.001	0.001
TCGA-F4-6806	CMS3	0.726	0.682	0.588	0.794	0.001	0.001
TCGA-G4-6317-1	CMS2	0.812	0.652	0.766	0.88	0.001	0.001
TCGA-CM-5868	CMS2	0.781	0.661	0.729	0.707	0.011	0.013
TCGA-CM-5861	CMS1	0.547	0.856	0.67	0.805	0.001	0.001
TCGA-AZ-5407	CMS3	0.712	0.735	0.618	0.917	0.001	0.001
TCGA-F4-6805	CMS4	0.604	0.733	0.661	0.41	0.001	0.001

TCGA-AZ-4682	CMS2	0.797	0.63	0.69	0.88	0.001	0.001
TCGA-D5-6898	CMS4	0.763	0.65	0.765	0.59	0.001	0.001
TCGA-AZ-4313	CMS3	0.664	0.829	0.631	0.879	0.001	0.001
TCGA-F4-6807	CMS4	0.662	0.718	0.724	0.402	0.001	0.001
TCGA-CM-4751	CMS1	0.574	0.755	0.638	0.701	0.001	0.001
TCGA-AY-6197	CMS1	0.634	0.763	0.684	0.861	0.001	0.001
TCGA-F4-6463	CMS4	0.732	0.637	0.669	0.483	0.001	0.001
TCGA-AZ-4315	Not assigned	0.676	0.785	0.72	0.842	0.067	0.073
TCGA-D5-5538	CMS1	0.668	0.701	0.701	0.669	0.006	0.007
TCGA-G4-6303	CMS2	0.782	0.605	0.754	0.658	0.001	0.001
TCGA-D5-6923	CMS4	0.737	0.632	0.781	0.448	0.001	0.001
TCGA-G4-6322	CMS3	0.718	0.749	0.54	0.828	0.001	0.001
TCGA-D5-5539	CMS3	0.703	0.761	0.508	0.669	0.001	0.001
TCGA-AH-6643	CMS1	0.667	0.759	0.764	0.7	0.036	0.04
TCGA-DC-4745	Not assigned	0.835	0.699	0.753	0.849	0.713	0.721
TCGA-DC-6683	CMS2	0.794	0.602	0.729	0.756	0.001	0.001
TCGA-EI-6508	CMS3	0.804	0.671	0.66	0.826	0.006	0.007
TCGA-EI-6917	CMS1	0.586	0.803	0.773	0.622	0.001	0.001
TCGA-EI-7004	CMS4	0.658	0.786	0.649	0.397	0.001	0.001
TCGA-EI-6513	CMS2	0.729	0.629	0.796	0.782	0.001	0.001
TCGA-DC-4749	CMS2	0.795	0.646	0.719	0.857	0.001	0.001
TCGA-F5-6814	CMS1	0.649	0.765	0.766	0.798	0.001	0.001
TCGA-DC-6156	CMS4	0.629	0.684	0.765	0.442	0.001	0.001
TCGA-DC-6154	CMS2	0.806	0.625	0.762	0.712	0.001	0.001
TCGA-F5-6465	CMS4	0.777	0.651	0.774	0.493	0.001	0.001
TCGA-EI-6507	CMS1	0.586	0.835	0.7	0.643	0.001	0.001
TCGA-DC-6682	Not assigned	0.774	0.7	0.722	0.846	0.732	0.732
TCGA-EF-5830	CMS2	0.755	0.629	0.719	0.854	0.001	0.001
TCGA-EI-6512	Not assigned	0.67	0.692	0.687	0.862	0.079	0.085
TCGA-EI-7002	CMS2	0.808	0.579	0.729	0.691	0.001	0.001

TCGA-EI-6882	CMS3	0.633	0.803	0.567	0.859	0.001	0.001
TCGA-EI-6885	CMS4	0.726	0.668	0.769	0.546	0.001	0.001
TCGA-F5-6861	CMS2	0.755	0.644	0.658	0.833	0.002	0.003
TCGA-EI-6883	CMS2	0.742	0.627	0.73	0.825	0.001	0.001

Supplementary Table 7. Genes with rare germline variants identified in patient 105 using 'All vs One' approach. This table lists genes identified through the 'All vs One' approach that harbour rare germline variants in patient 105. Genes shown meet the differential expression threshold of |log2 fold change| ≥1.5 and FDR <5%. Variants were retained on filtering criteria for rare population frequency. logFC: log2 fold change in gene expression; logCPM: log2-transformed counts per million across all samples; F: quasi-likelihood F-test statistic; p-value: nominal p-value (uncorrected); FDR: p-value adjusted using Benjamini-Hochberg correction.

	**logFC**	**logCPM**	**F**	**p-value**	**FDR**
SLC6A6	3.48	4.43	119.51	1.67×10^{-16}	6.12x10-14
FBXO41	2.38	3.10	73.87	2.14×10^{-12}	3.10x10-10
GDPD5	2.41	2.80	57.13	1.56×10^{-10}	1.59×10^{-8}
ALDH3B2	4.89	-2.08	44.59	5.84×10^{-9}	3.82×10^{-7}
MDFI	4.03	0.38	42.49	1.12×10^{-8}	6.75×10^{-7}
DNAH14	-3.63	1.50	36.79	6.96×10^{-8}	3.37×10^{-6}
ADAMTSL1	-2.93	3.76	27.90	1.51×10^{-6}	4.75×10^{-5}
RNF130	-1.54	5.57	27.61	1.68×10^{-6}	5.16×10^{-5}
HTR4	-4.33	2.56	26.38	2.64×10^{-6}	7.39×10^{-5}
DNAH10	2.41	-3.09×10^{-3}	24.76	4.85×10^{-6}	1.22×10^{-4}
LRRC34	2.45	-0.58	24.74	4.88×10^{-6}	1.22×10^{-4}
CTU2	1.59	3.14	23.64	7.43×10^{-6}	1.73×10^{-4}
TAF4B	1.65	2.63	22.68	1.07×10^{-5}	2.38×10^{-4}
AKR1C1	3.54	4.06	20.95	2.12×10^{-5}	4.07×10^{-4}
PDGFB	1.80	3.31	18.81	5.00×10^{-5}	8.10×10^{-4}
IQUB	3.14	-3.05	18.26	6.27×10^{-5}	9.67×10^{-4}
KIF19	-3.66	2.34	17.24	9.58×10^{-5}	1.36×10^{-3}
PLSCR4	-1.62	5.15	15.31	2.17×10^{-4}	2.63×10^{-3}
NAP1L2	-3.47	1.69	15.07	2.40×10^{-4}	2.84×10^{-3}
SGO2	2.01	2.80	13.36	5.09×10^{-4}	5.16×10^{-3}
TULP1	3.23	-2.92	12.68	6.87×10^{-4}	6.49×10^{-3}
PTP4A3	2.21	3.99	12.22	8.48×10^{-4}	7.59×10^{-3}
A2ML1	3.25	-1.21	12.16	8.69×10^{-4}	7.73×10^{-3}
TRPV5	3.25	-4.38	10.37	1.98×10^{-3}	1.47×10^{-2}
CCDC150	1.85	0.82	10.17	2.18×10^{-3}	1.58×10^{-2}
LHX4	-1.57	2.98	9.69	2.73×10^{-3}	1.86×10^{-2}
CEMIP	3.50	2.70	9.01	3.78×10^{-3}	2.37×10^{-2}

Supplementary Table 8. Genes with rare germline variants identified in patient 401 using 'All vs One' approach. This table lists genes identified through the 'All vs One' approach that harbour rare germline variants in patient 401. Genes shown meet the differential expression threshold of |log2 fold change| ≥1.5 and FDR <5%. Variants were retained on filtering criteria for rare population frequency. logFC: log2 fold change in gene expression; logCPM: log2-transformed counts per million across all samples; F: quasi-likelihood F-test statistic; p-value: nominal p-value (uncorrected); FDR: p-value adjusted using Benjamini-Hochberg correction.

	logFC	logCPM	F	p-value	FDR
ZNF350	-2.35	3.38	31.38	$4.37x10^{-7}$	$6.45x10^{-5}$
ZNF793	-2.72	2.70	27.18	$1.97x10^{-6}$	$2.17x10^{-4}$
TNFSF9	4.87	0.07	24.68	$4.99x10^{-6}$	$4.39x10^{-4}$
MROH7	-3.22	4.34	17.70	$7.90x10^{-5}$	$3.27x10^{-3}$
OR2W3	-8.74	-0.46	16.99	$1.06x10^{-4}$	$4.03x10^{-3}$
SCN5A	-3.41	0.02	16.33	$1.41x10^{-4}$	$4.91x10^{-3}$
ERBIN	-1.75	6.61	14.47	$3.11x10^{-4}$	$8.61x10^{-3}$
ANK1	2.93	-0.24	13.25	$5.33x10^{-4}$	$1.24x10^{-2}$
SPATA46	-6.17	-1.75	13.19	$5.47x10^{-4}$	$1.26x10^{-2}$
SPON2	1.52	5.32	11.98	$9.45x10^{-4}$	$1.83x10^{-2}$
SLCO4A1	1.93	4.19	10.79	$1.63x10^{-3}$	$2.64x10^{-2}$
WDR4	1.74	2.93	10.66	$1.73x10^{-3}$	$2.76x10^{-2}$
IGSF9	-2.79	6.10	9.07	$3.66x10^{-3}$	$4.52x10^{-2}$
PKD1L1	-1.59	0.39	8.97	$3.85x10^{-3}$	$4.62x10^{-2}$
TTLL6	-2.49	4.11	8.91	$3.96x10^{-3}$	$4.69x10^{-2}$
VWA5A	-1.65	6.36	8.75	$4.29x10^{-3}$	$4.90x10^{-2}$

Supplementary Table 9. Genes with rare germline variants identified in patient TCGA-AH-6643 using 'All vs One' approach. This table lists genes identified through the 'All vs One' approach that harbour rare germline variants in patient TCGA-AH-6643. Genes shown meet the differential expression threshold of |log2 fold change| ≥1.5 and FDR <5%. Variants were retained on filtering criteria for rare population frequency. logFC: log2 fold change in gene expression; logCPM: log2-transformed counts per million across all samples; F: quasi-likelihood F-test statistic; p-value: nominal p-value (uncorrected); FDR: p-value adjusted using Benjamini-Hochberg correction.

	logFC	logCPM	F	p-value	FDR
ASB4	8.31	-0.25	437.51	5.62×10^{-31}	9.98×10^{-28}
IQGAP1	3.05	8.32	324.07	2.79×10^{-27}	2.48×10^{-24}
KIAA1549	3.29	2.70	169.47	5.74×10^{-20}	1.35×10^{-17}
COPA	1.62	7.58	143.3	2.94×10^{-18}	4.75×10^{-16}
HABP2	5.56	-1.70	124.39	6.98×10^{-17}	8.36×10^{-15}
PCNX2	2.62	3.44	112.93	5.57×10^{-16}	5.74×10^{-14}
SPRTN	2.18	3.21	86.47	1.18×10^{-13}	7.52×10^{-12}
DNAH2	3.40	0.10	84.04	2.03×10^{-13}	1.23×10^{-11}
TBCA	1.61	5.41	73.65	2.26×10^{-12}	1.02×10^{-10}
HDAC2	1.58	6.10	61.97	4.25×10^{-11}	1.43×10^{-9}
FGF18	4.51	-0.95	60.87	5.68×10^{-11}	1.85×10^{-9}
MUC4	-8.40	8.47	58.03	1.22×10^{-10}	3.68×10^{-9}
KLC3	2.68	-1.22	46.09	3.70×10^{-9}	7.93×10^{-8}
RHBDL2	-4.18	4.54	41.54	1.50×10^{-8}	2.74×10^{-7}
RILP	-4.01	4.74	36.18	8.51×10^{-8}	1.28×10^{-6}
MAMDC4	-4.00	5.47	35.49	1.07×10^{-7}	1.56×10^{-6}
IL6R	-2.57	5.95	34.99	1.27×10^{-7}	1.82×10^{-6}
POLR1B	1.89	4.24	34.49	1.50×10^{-7}	2.11×10^{-6}
TAF1B	1.69	2.34	29.95	7.24×10^{-7}	8.51×10^{-6}
GALM	-2.37	5.92	29.92	7.30×10^{-7}	8.57×10^{-6}
UGT1A6	-6.68	6.61	27.60	1.69×10^{-6}	1.79×10^{-5}
CCDC17	-4.29	1.51	27.17	1.97×10^{-6}	2.05×10^{-5}
SLC9A9	-3.24	3.14	27.12	2.01×10^{-6}	2.08×10^{-5}
IL15	-2.67	2.87	25.11	4.25×10^{-6}	3.99×10^{-5}
SEC23B	1.55	5.86	24.31	5.74×10^{-6}	5.21×10^{-5}
GCG	-12.51	5.77	23.34	8.33×10^{-6}	7.20×10^{-5}
IGSF10	2.87	2.26	22.71	1.06×10^{-5}	8.80×10^{-5}
NEXMIF	-6.00	1.43	22.31	1.24×10^{-5}	1.00×10^{-4}
WNT10B	-4.17	0.42	22.18	1.31×10^{-5}	1.05×10^{-4}
PCNA	1.71	5.96	21.91	1.45×10^{-5}	1.15×10^{-4}
FAM135B	-5.92	1.44	21.18	1.94×10^{-5}	1.48×10^{-4}

BRCA1	2.02	3.43	20.72	2.32×10^{-5}	1.73×10^{-4}
SLAMF9	4.11	-3.91	20.56	2.47×10^{-5}	1.82×10^{-4}
IL12RB2	-3.94	-0.40	19.09	4.46×10^{-5}	3.02×10^{-4}
TRPM6	-7.82	6.57	18.40	5.93×10^{-5}	3.86×10^{-4}
KCNAB3	-2.64	1.87	17.81	7.57×10^{-5}	4.77×10^{-4}
U2AF1L4	-1.83	3.62	17.15	9.95×10^{-5}	6.01×10^{-4}
FOXA2	2.07	4.10	17.10	1.02×10^{-4}	6.12×10^{-4}
MYSM1	-1.77	6.08	16.20	1.48×10^{-4}	8.43×10^{-4}
KSR1	-1.99	4.74	16.16	1.51×10^{-4}	8.56×10^{-4}
LAMC3	-3.37	2.85	12.37	7.90×10^{-4}	3.51×10^{-3}
SH3RF2	-1.52	6.20	11.49	1.18×10^{-3}	4.90×10^{-3}
RTN4RL1	-3.37	-0.45	11.33	1.27×10^{-3}	5.21×10^{-3}
RAD51AP2	2.69	-2.24	11.23	1.33×10^{-3}	5.40×10^{-3}
CCDC62	-3.07	-1.78	10.87	1.57×10^{-3}	6.18×10^{-3}
SCN7A	-5.52	4.53	10.76	1.65×10^{-3}	6.46×10^{-3}
OGN	-5.39	3.79	10.59	1.79×10^{-3}	6.92×10^{-3}
EQTN	-6.94	-2.42	10.14	2.21×10^{-3}	8.27×10^{-3}
DCLK1	-3.48	2.87	9.50	2.98×10^{-3}	1.07×10^{-2}
PTCH2	-2.64	1.58	9.40	3.14×10^{-3}	1.11×10^{-2}
CYP46A1	-2.94	-0.69	9.18	3.48×10^{-3}	1.21×10^{-2}
RMDN2	-1.62	4.02	8.81	4.16×10^{-3}	1.41×10^{-2}
SGO2	1.61	2.70	8.49	4.87×10^{-3}	1.60×10^{-2}
CYP4B1	-9.49	3.61	8.30	5.33×10^{-3}	1.73×10^{-2}
COL9A2	-2.86	4.04	7.96	6.30×10^{-3}	1.99×10^{-2}
ABCD2	-2.51	0.05	7.92	6.41×10^{-3}	2.01×10^{-2}
ZNF667	-2.78	1.22	7.48	8.00×10^{-3}	2.42×10^{-2}
ANKRD36	-1.59	2.31	7.44	8.16×10^{-3}	2.46×10^{-2}
SLC44A3	-1.52	5.70	7.43	8.19×10^{-3}	2.46×10^{-2}
KCNB1	-5.49	2.66	7.37	8.43×10^{-3}	2.52×10^{-2}
FAT2	-1.96	0.69	7.35	8.52×10^{-3}	2.55×10^{-2}
TEX14	-1.59	-0.89	7.29	8.79×10^{-3}	2.61×10^{-2}
RTP5	-7.43	-2.18	7.28	8.84×10^{-3}	2.62×10^{-2}
FLG2	-8.28	-1.33	6.66	1.21×10^{-2}	3.36×10^{-2}
SYNE1	-2.23	5.58	6.64	1.22×10^{-2}	3.39×10^{-2}
SARDH	-2.07	0.50	6.35	1.41×10^{-2}	3.82×10^{-2}
PER3	-3.32	4.47	5.99	1.70×10^{-2}	4.43×10^{-2}
PTCD	-3.90	-1.07	5.94	1.75×10^{-2}	4.53×10^{-2}
DNAH6	1.59	1.16	5.92	1.77×10^{-2}	4.56×10^{-2}
SLC47A2	-3.23	-1.48	5.77	1.91×10^{-2}	4.85×10^{-2}
ADRA1A	-9.02	-0.65	15.66	1.86×10^{-4}	1.03×10^{-3}
PNMA3	-5.91	9.70	14.98	2.50×10^{-4}	1.32×10^{-3}
SPECC1	-2.15	5.94	14.52	3.06×10^{-4}	1.57×10^{-3}
PLIN5	-3.50	1.98	14.29	3.37×10^{-4}	1.71×10^{-3}

LRRN2	-4.47	4.71	14.24	3.44×10^{-4}	1.74×10^{-3}
USP9Y	-4.13	4.03	14.21	3.49×10^{-4}	1.76×10^{-3}
CDH26	-2.47	-0.18	14.04	3.76×10^{-4}	1.87×10^{-3}
FAM13A	-1.85	5.86	13.92	3.96×10^{-4}	1.96×10^{-3}
ADGB	-4.42	-0.13	13.87	4.06×10^{-4}	2.00×10^{-3}
SCIN	-3.30	5.84	12.64	7.02×10^{-4}	3.18×10^{-3}
RIC3	-5.07	2.56	12.56	7.25×10^{-4}	3.27×10^{-3}
HJURP	2.10	3.00	12.54	7.31×10^{-4}	3.29×10^{-3}

Supplementary Table 10. Genes with rare germline variants identified in patient 303 using 'All vs One' approach. This table lists genes identified through the 'All vs One' approach that harbour rare germline variants in patient 303. Genes shown meet the differential expression threshold of |log2 fold change| ≥1.5 and FDR <5%. Variants were retained on filtering criteria for rare population frequency. logFC: log2 fold change in gene expression; logCPM: log2-transformed counts per million across all samples; F: quasi-likelihood F-test statistic; p-value: nominal p-value (uncorrected); FDR: p-value adjusted using Benjamini-Hochberg correction.

	logFC	logCPM	F	p-value	FDR
ASAP1	2.30	4.48	74.71	1.76×10^{-12}	4.06×10^{-10}
TRIM54	4.80	-1.00	70.68	4.65×10^{-12}	9.80×10^{-10}
RELL2	2.09	2.65	49.76	1.25×10^{-9}	1.41×10^{-7}
PLEKHN1	2.76	0.20	47.03	2.79×10^{-9}	2.91×10^{-7}
ABCC1	1.87	5.14	44.09	6.81×10^{-9}	6.36×10^{-7}
KCNJ15	5.04	-1.42	38.11	4.52×10^{-8}	3.30×10^{-6}
PLEKHA4	2.90	3.67	25.71	$3.39 \mathrm{X} 10^{-6}$	$1.31 \mathrm{X} 10^{-4}$
COL5A3	2.12	4.76	25.42	3.77×10^{-6}	1.43×10^{-4}
CCN4	3.97	0.90	22.77	1.04×10^{-5}	3.27×10^{-4}
SERPINF2	2.36	1.75	21.09	2.00×10^{-5}	5.54×10^{-4}
DNAH10	2.29	-0.01	20.98	2.09×10^{-5}	5.76×10^{-4}
ERAP1	-1.73	6.81	19.80	3.36×10^{-5}	8.24×10^{-4}
CA9	3.30	-0.57	18.74	5.16×10^{-5}	1.18×10^{-3}
CDH16	3.84	0.10	18.50	5.69×10^{-5}	1.28×10^{-3}
ITGBL1	3.28	-0.71	15.69	1.84×10^{-4}	3.29×10^{-3}
MTFR2	2.08	1.34	13.90	4.01×10^{-4}	5.95×10^{-3}
LEF1	2.17	2.03	13.46	4.86×10^{-4}	6.90×10^{-3}
ELOVL3	3.44	-3.05	10.57	1.80×10^{-3}	1.81×10^{-2}
POLQ	2.02	2.99	10.47	1.89×10^{-3}	1.87×10^{-2}
SELP	1.71	2.16	10.45	1.91×10^{-3}	1.88×10^{-2}
SCIN	-2.83	5.84	9.95	2.41×10^{-3}	2.21×10^{-2}
BICD1	1.62	2.15	9.88	2.50×10^{-3}	2.27×10^{-2}
CPXM1	2.54	1.63	9.67	2.75×10^{-3}	2.42×10^{-2}
SLC6A20	3.41	1.32	9.63	2.81×10^{-3}	2.46×10^{-2}
PNPLA7	-1.93	5.19	9.11	3.60×10^{-3}	2.92×10^{-2}
LGALS4	-1.89	11.43	8.40	5.08×10^{-3}	3.76×10^{-2}
MEFV	1.89	0.46	8.20	5.59×10^{-3}	4.01×10^{-2}
ARHGEF4	2.89	2.23	7.81	6.77×10^{-3}	4.58×10^{-2}
MYO1H	1.78	-2.19	7.64	7.39×10^{-3}	4.85×10^{-2}
DLC1	1.79	4.65	7.55	7.70×10^{-3}	4.99×10^{-2}

Supplementary Table 11. Genes with rare germline variants identified in patient 310 using 'All vs One' approach. This table lists genes identified through the 'All vs One' approach that harbour rare germline variants in patient 310. Genes shown meet the differential expression threshold of |log2 fold change| ≥1.5 and FDR <5%. Variants were retained on filtering criteria for rare population frequency. logFC: log2 fold change in gene expression; logCPM: log2-transformed counts per million across all samples; F: quasi-likelihood F-test statistic; p-value: nominal p-value (uncorrected); FDR: p-value adjusted using Benjamini-Hochberg correction.

	**logFC**	**logCPM**	**F**	**p-value**	**FDR**
TMEM132C	6.41	1.71	272.60	3.14×10^{-25}	1.34×10^{-21}
ASPSCR1	2.08	4.25	122.15	1.04×10^{-16}	4.42×10^{-14}
MOCS3	1.72	2.82	74.77	1.73×10^{-12}	2.68×10^{-10}
PKLR	4.86	0.50	60.84	5.73×10^{-11}	5.97×10^{-9}
COL18A1	2.91	6.53	59.89	7.38×10^{-11}	7.55×10^{-9}
FMO1	5.24	-2.21	52.91	5.05×10^{-10}	3.98×10^{-8}
PCDH17	3.60	1.54	50.61	9.75×10^{-10}	6.93×10^{-8}
L3MBTL1	1.95	3.48	48.68	1.71×10^{-9}	1.14×10^{-7}
FAM9A	4.97	-4.87	48.73	2.07×10^{-9}	1.34×10^{-7}
NOTCH4	2.36	3.85	47.66	2.31×10^{-9}	1.47×10^{-7}
OSM	5.71	-0.42	44.67	5.70×10^{-9}	3.19×10^{-7}
WFS1	2.76	5.10	42.06	1.28×10^{-8}	6.50×10^{-7}
ENG	2.01	6.18	38.61	3.83×10^{-8}	1.73×10^{-6}
LRCH1	1.77	4.58	37.27	5.94×10^{-8}	2.57×10^{-6}
PALM3	2.61	0.01	20.98	2.09×10^{-5}	3.56×10^{-4}
RP1L1	3.95	-2.65	20.32	2.72×10^{-5}	4.41×10^{-4}
HECW1	2.46	-1.27	18.95	4.74×10^{-5}	6.97×10^{-4}
NUTM1	-6.56	-0.89	16.63	1.24×10^{-4}	1.53×10^{-3}
SKA3	2.58	2.04	14.50	3.08×10^{-4}	3.20×10^{-3}
TST	-2.60	7.95	14.39	3.23×10^{-4}	3.32×10^{-3}
MUCL1	5.31	-3.67	13.78	4.35×10^{-4}	4.22×10^{-3}
CARD10	-1.54	5.95	12.57	7.21×10^{-4}	6.31×10^{-3}
SLC26A9	3.71	-3.86	12.43	7.69×10^{-4}	6.62×10^{-3}
LRIT2	-3.75	1.89	11.22	1.34×10^{-3}	1.02×10^{-2}
ABCA10	-2.20	3.03	10.73	1.67×10^{-3}	1.21×10^{-2}
MUC5B	-3.64	8.42	10.61	1.77×10^{-3}	1.26×10^{-2}
STAP2	-1.85	6.91	9.84	2.54×10^{-3}	1.67×10^{-2}
WNK4	1.91	5.94	8.74	4.30×10^{-3}	2.48×10^{-2}
TG	3.58	2.05	8.62	4.55×10^{-3}	2.59×10^{-2}
SDK2	-2.95	2.10	8.42	5.10×10^{-3}	2.79×10^{-2}
SLC4A11	1.76	0.05	8.37	5.14×10^{-3}	2.84×10^{-2}
KIAA1614	1.64	2.58	7.96	6.31×10^{-3}	3.31×10^{-2}

Supplementary Table 12. Genes with rare germline variants identified in patient TCGA-A6-5662 using 'All vs One' approach. This table lists genes identified through the 'All vs One' approach that harbour rare germline variants in patient TCGA-A6-5662. Genes shown meet the differential expression threshold of |log2 fold change| ≥1.5 and FDR <5%. Variants were retained on filtering criteria for rare population frequency. logFC: log2 fold change in gene expression; logCPM: log2-transformed counts per million across all samples; F: quasi-likelihood F-test statistic; p-value: nominal p-value (uncorrected); FDR: p-value adjusted using Benjamini-Hochberg correction.

	**logFC**	**logCPM**	**F**	**p-value**	**FDR**
COG6	2.33	4.65	135.16	1.11x10^{-17}	1.49x10^{-15}
ESR2	-4.58	0.80	35.40	1.10x10^{-7}	1.84x10^{-6}
RUVBL1	1.52	5.00	32.83	2.64x10^{-7}	3.91x10^{-6}
DEPDC5	-1.71	4.72	30.18	6.67x10^{-7}	8.67x10^{-6}
SIGLEC1	-4.53	4.91	29.61	8.16x10^{-7}	1.03x10^{-5}
RNF207	-2.96	4.91	24.82	4.73x10^{-6}	4.80x10^{-5}
MAST2	-2.11	7.34	21.84	1.49x10^{-5}	1.27x10^{4}
ADSS1	-2.33	1.74	21.55	1.67x10^{-5}	1.40x10^{-4}
TTN	-2.95	4.37	20.76	2.28x10^{-5}	1.83x10^{-4}
RASGRP2	-4.64	4.30	19.14	4.37x10^{-5}	3.18x10^{-4}
APLF	1.74	1.08	17.95	7.14x10^{-5}	4.84x10^{-4}
CPT2	-1.80	6.27	17.60	8.24x10^{-5}	5.48x10^{-4}
SCNN1D	-2.93	2.54	17.29	9.38x10^{-5}	6.12x10^{-4}
RBM6	-1.54	7.15	16.84	1.13x10^{-4}	7.18x10^{-4}
AHNAK	-2.54	10.53	15.99	1.62X10^{-4}	9.75x10^{-4}
CLIC6	-5.46	4.06	14.73	2.78x10^{-4}	1.54x10^{-3}
PYGL	-2.99	5.36	14.35	3.28x10^{-4}	1.76x10^{-3}
NFATC4	-2.95	5.29	13.10	5.71x10^{-4}	2.82x10^{-3}
STOX2	-2.42	2.67	12.71	6.80x10^{-4}	3.25x10^{-3}
ASIC1	2.44	2.12	10.34	2.01x10^{-}	8.00x10^{-3}
NTRK3	-3.66	1.63	10.11	2.24x10^{-3}	8.75x10^{-3}
RUFY4	-6.06	-0.85	9.89	2.48x10^{-3}	9.51x10^{-3}
KIFC3	1.71	3.44	9.38	3.16x10^{-3}	1.16x10^{-2}
CASP5	-4.87	3.75	9.33	3.23x10^{-3}	1.18x10^{-2}
CPA1	-12.06	2.12	8.22	5.55x10^{-3}	1.86x10^{-2}
SDK2	-2.80	2.10	7.81	6.79x10^{-3}	2.19x10^{-2}
ZNF540	-1.52	1.76	6.56	1.27x10^{-2}	3.64x10^{-2}
CNTN4	-2.15	3.01	6.34	1.42x10^{-2}	3.99x10^{-2}
CNGA3	-4.99	1.01	6.06	1.64x10^{-2}	4.48x10^{-2}
SUGCT	-2.02	2.54	6.03	1.67x10^{-2}	4.54x10^{-2}

Supplementary Table 13. Genes with rare germline variants identified in patient TCGA-A6-5667 using 'All vs One' approach. This table lists genes identified through the 'All vs One' approach that harbour rare germline variants in patient TCGA-A6-5667. Genes shown meet the differential expression threshold of |log2 fold change| ≥1.5 and FDR <5%. Variants were retained on filtering criteria for rare population frequency. logFC: log2 fold change in gene expression; logCPM: log2-transformed counts per million across all samples; F: quasi-likelihood F-test statistic; p-value: nominal p-value (uncorrected); FDR: p-value adjusted using Benjamini-Hochberg correction.

	logFC	logCPM	F	p-value	FDR
PRKDC	3.07	6.85	212.36	$2.15x10^{-22}$	$1.53x10^{-19}$
PKP4	1.54	5.76	94.07	$2.32x10^{-14}$	$2.03x10^{-12}$
STAU2	1.54	4.68	85.68	$1.41x10^{-13}$	$1.01x10^{-11}$
TRAM1	1.88	6.80	74.30	$1.93x10^{-12}$	$1.11x10^{-10}$
NUP155	1.87	4.44	68.90	$7.21x10^{-12}$	$3.56x10^{-10}$
SPAG1	2.12	5.20	49.64	$1.29x10^{-9}$	$3.48x10^{-8}$
ABCA10	-5.63	3.02	42.93	$9.73x10^{-9}$	$2.03x10^{-7}$
ENC1	2.40	5.60	39.28	$3.09x10^{-8}$	$5.58x10^{-7}$
ZNF518B	-2.54	3.95	37.25	$5.97x10^{-8}$	$9.98x10^{-7}$
PAN2	-2.02	5.95	35.95	$9.17x10^{-8}$	$1.47x10^{-6}$
PLTP	2.79	5.84	31.83	$3.74x10^{-7}$	$5.12x10^{-6}$
PITPNM3	-2.51	5.36	28.56	$1.19x10^{-6}$	$1.39x10^{-5}$
TBX18	3.09	-0.31	24.16	$6.09x10^{-6}$	$5.88x10^{-5}$
SPAG8	-2.90	1.66	21.14	$1.97x10^{-5}$	$1.62x10^{-4}$
TIAM1	-2.09	2.74	18.86	$4.91x10^{-5}$	$3.54x10^{-4}$
BRCA2	2.40	2.00	17.94	$7.15x10^{-5}$	$4.89x10^{-4}$
ADAMTS10	-3.14	3.35	17.60	$8.24x10^{-5}$	$5.52x10^{-4}$
TRPM5	-5.00	2.97	17.35	$9.15x10^{-5}$	$6.04x10^{-4}$
TTN	-2.41	4.37	15.05	$2.42x10^{-4}$	$1.37x10^{-3}$
ROBO2	2.55	0.63	14.76	$2.75x10^{-4}$	$1.53x10^{-3}$
SLC6A4	3.64	0.65	13.43	$4.92x10^{-4}$	$2.50x10^{-3}$
FRMD4B	-2.07	4.75	12.10	$8.92x10^{-4}$	$4.11x10^{-3}$
AGMAT	1.55	4.25	11.50	$1.18x10^{-3}$	$5.16x10^{-3}$
SIGLEC11	-3.81	-0.50	11.37	$1.25x10^{-3}$	$5.43x10^{-3}$
RGS6	-3.79	-0.34	11.35	$1.26x10^{-3}$	$5.48x10^{-3}$
CACNG1	3.71	-4.12	11.25	$1.32x10^{-3}$	$5.70x10^{-3}$
TMEM17	1.63	-0.15	11.22	$1.33x10^{-3}$	$5.75x10^{-3}$
RYR1	-2.25	1.47	10.86	$1.58x10^{-3}$	$6.59x10^{-3}$
PTX3	-4.67	2.30	10.46	$1.90x10^{-3}$	$7.68x10^{-3}$
KLRC3	-4.26	-0.89	10.44	$1.92x10^{-3}$	$7.74x10^{-3}$
LY9	-3.26	1.96	10.27	$2.08x10^{-3}$	$8.27x10^{-3}$

ROBO3	-2.51	3.13	9.84	$2.54x10^{-3}$	$9.77x10^{-3}$
ADAMTS15	-3.48	3.15	9.64	$2.80x10^{-3}$	$1.06x10^{-2}$
ASPM	1.89	3.50	9.58	$2.87x10^{-3}$	$1.08x10^{-2}$
SLC9C1	-2.84	-1.30	9.24	$3.38x10^{-3}$	$1.23x10^{-2}$
MUC6	-4.13	-0.21	8.93	$3.93x10^{-3}$	$1.39x10^{-2}$
CCDC178	-4.22	-2.41	8.69	$4.42x10^{-3}$	$1.53x10^{-2}$
VWA5B1	2.90	-1.06	8.67	$4.44x10^{-3}$	$1.54x10^{-2}$
TRPV3	-5.32	3.40	7.33	$8.62x10^{-3}$	$2.64x10^{-2}$
THY1	2.08	4.34	7.28	$8.85x10^{-3}$	$2.70x10^{-2}$
TRIM7	-2.15	2.08	6.94	$1.04x10^{-2}$	$3.09x10^{-2}$
MUC17	-6.12	5.28	6.79	$1.13x10^{-2}$	$3.30x10^{-2}$
PLEKHG4B	-5.74	0.24	6.47	$1.33x10^{-2}$	$3.75x10^{-2}$
RIMS1	1.90	-1.83	5.91	$1.78x10^{-2}$	$4.77x10^{-2}$
CRB1	-6.06	-3.01	5.90	$1.78x10^{-2}$	$4.78x10^{-2}$

Supplementary Table 14. Genes with rare germline variants identified in patient TCGA-CM-6164 using 'All vs One' approach. This table lists genes identified through the 'All vs One' approach that harbour rare germline variants in patient TCGA-CM-6164. Genes shown meet the differential expression threshold of |log2 fold change| ≥1.5 and FDR <5%. Variants were retained on filtering criteria for rare population frequency. logFC: log2 fold change in gene expression; logCPM: log2-transformed counts per million across all samples; F: quasi-likelihood F-test statistic; p-value: nominal p-value (uncorrected); FDR: p-value adjusted using Benjamini-Hochberg correction.

	**logFC**	**logCPM**	**F**	**p-value**	**FDR**
RAE1	2.10	4.62	174.58	$2.80x10^{-20}$	$1.57x10^{-17}$
NEBL	3.76	4.28	107.02	$1.71x10^{-15}$	$2.99x10^{-13}$
TCF20	1.53	5.62	93.93	$2.39x10^{-14}$	$3.03x10^{-12}$
MAGEA4	6.64	-4.78	77.63	$1.22x10^{-11}$	$8.27x10^{-10}$
MPP1	1.80	4.60	49.61	$1.30x10^{-9}$	$5.12x10^{-8}$
HOXA3	-4.94	4.49	44.39	$6.20x10^{-9}$	$2.00x10^{-7}$
DNHD1	-2.91	4.67	42.68	$1.05x10^{-8}$	$3.19x10^{-7}$
SLC13A3	3.51	-0.46	40.18	$2.31x10^{-8}$	$6.34x10^{-7}$
EVPL	1.57	5.37	39.67	$2.72x10^{-8}$	$7.30x10^{-7}$
FGD5	1.71	3.82	32.70	$2.76x10^{-7}$	$5.51x10^{-6}$
ADSS1	-3.03	1.74	31.96	$3.57x10^{-7}$	$6.85x10^{-6}$
PKHD1L1	-7.66	2.45	30.21	$6.60x10^{-7}$	$1.18x10^{-5}$
HOXA9	-2.64	4.84	22.84	$1.01x10^{-5}$	$1.21x10^{-4}$
TAP1	1.81	6.62	21.44	$1.74x10^{-5}$	$1.94x10^{-4}$
SLC46A3	-2.27	5.68	20.70	$2.34x10^{-5}$	$2.49x10^{-4}$
SI	-8.09	6.45	20.70	$2.34x10^{-5}$	$2.49x10^{-4}$
FAM83H	1.63	6.27	18.94	$4.74x10^{-5}$	$4.46x10^{-4}$
NAALADL2	1.60	2.94	18.92	$4.80x10^{-5}$	$4.50x10^{-4}$
ISG15	2.40	3.63	18.46	$5.77x10^{-5}$	$5.24x10^{-4}$
COL28A1	-4.18	3.29	16.42	$1.35x10^{-4}$	$1.07x10^{-3}$
ADAMTSL5	-2.56	1.83	16.10	$1.55x10^{-4}$	$1.19x10^{-3}$
APCDD1	2.13	4.65	16.06	$1.57x10^{-4}$	$1.21x10^{-3}$
SNED1	-1.63	4.81	13.68	$4.40x10^{-4}$	$2.81x10^{-3}$
CASQ1	-5.74	-0.38	12.06	$9.10x10^{-4}$	$5.07x10^{-3}$
SLC14A1	-2.51	1.34	11.21	$1.34x10^{-3}$	$6.98x10^{-3}$
PLCD1	-2.18	5.86	10.51	$1.86x10^{-3}$	$9.14x10^{-3}$
NFATC4	-2.49	5.28	10.39	$1.96x10^{-3}$	$9.54x10^{-3}$
SLC27A6	-3.39	-0.32	10.26	$2.08x10^{-3}$	$1.00x10^{-2}$
IL22RA1	1.87	5.08	10.22	$2.12x10^{-3}$	$1.01x10^{-2}$
TTN	-1.89	4.37	10.12	$2.23x10^{-3}$	$1.06x10^{-2}$
PLCH2	-3.36	2.80	9.96	$2.41x10^{-3}$	$1.12x10^{-2}$

PIK3C2G	-7.89	-1.36	9.94	$2.43x10^{-3}$	$1.13x10^{-2}$
GPR150	-3.54	-0.93	9.34	$3.23x10^{-3}$	$1.43x10^{-2}$
TNXB	-3.30	5.49	9.23	$3.40x10^{-3}$	$1.49x10^{-2}$
STAB2	-3.54	1.27	8.99	$3.81x10^{-3}$	$1.63x10^{-2}$
CDHR5	-2.16	8.76	8.85	$4.09x10^{-3}$	$1.71x10^{-2}$
ASPG	-5.24	3.00	8.37	$5.15x10^{-3}$	$2.06x10^{-2}$
ITGA10	-4.13	3.69	8.19	$5.62x10^{-3}$	$2.21x10^{-2}$
STPG3	-3.76	0.05	8.09	$5.91x10^{-3}$	$2.29x10^{-2}$
TEX38	-6.46	-3.14	7.91	$6.46x10^{-3}$	$2.46x10^{-2}$
SASH1	-1.55	4.97	7.90	$6.47x10^{-3}$	$2.46x10^{-2}$
HMGCS2	-2.91	9.31	7.87	$6.57x10^{-3}$	$2.49x10^{-2}$
HHIPL2	-3.56	-1.96	7.86	$6.61x10^{-3}$	$2.50x10^{-2}$
KRT222	-2.74	-0.66	7.82	$6.74x10^{-3}$	$2.54x10^{-2}$
CATSPERG	-2.13	1.40	7.74	$7.01x10^{-3}$	$2.63x10^{-2}$
SHISA9	2.07	1.06	7.73	$7.07x10^{-3}$	$2.64x10^{-2}$
C16orf89	-3.53	1.56	7.69	$7.20x10^{-3}$	$2.68x10^{-2}$
SPATA31E1	-6.73	-2.53	7.68	$7.22x10^{-3}$	$2.68x10^{-2}$
SKOR1	-2.46	0.01	6.21	$1.52x10^{-2}$	$4.80x10^{-2}$

Supplementary Table 15. Genes with rare germline variants identified in patient TCGA-CM-6166 using 'All vs One' approach. This table lists genes identified through the 'All vs One' approach that harbour rare germline variants in patient TCGA-CM-6166. Genes shown meet the differential expression threshold of |log2 fold change| ≥1.5 and FDR <5%. Variants were retained on filtering criteria for rare population frequency. logFC: log2 fold change in gene expression; logCPM: log2-transformed counts per million across all samples; F: quasi-likelihood F-test statistic; p-value: nominal p-value (uncorrected); FDR: p-value adjusted using Benjamini-Hochberg correction.

	logFC	logCPM	F	p-value	FDR
NONO	2.06	7.68	298.32	$2.73x10^{-26}$	$3.74x10^{-23}$
ZNRF3	3.34	3.92	195.02	$1.85x10^{-21}$	$6.91x10^{-19}$
URI1	1.64	5.61	119.94	$1.54x10^{16}$	$1.68x10^{-14}$
HAS2	3.76	2.33	83.84	$2.12x10^{-13}$	$1.13x10^{-11}$
MTBP	2.38	1.69	62.69	$3.52x10^{-11}$	$1.06x10^{-9}$
FOXRED2	2.54	2.89	61.90	$4.33x10^{-11}$	$1.28x10^{-9}$
WDR75	1.69	4.99	59.36	$8.50x10^{-11}$	$2.35x10^{-9}$
MACC1	3.24	3.70	59.33	$8.59x10^{-11}$	$2.37x10^{-9}$
NCKAP5	2.96	1.79	52.22	$6.13x10^{-1}$	$1.39x10^{-8}$
CAD	1.58	5.36	48.83	$1.64x10^{-9}$	$3.39x10^{-8}$
TRIM28	1.51	7.45	46.23	$3.55x10^{-9}$	$6.72x10^{-8}$
STAB1	-4.47	7.47	38.94	$3.44x10^{-8}$	$4.96x10^{-7}$
MAMDC4	-4.13	5.47	36.70	$7.17x10^{-8}$	$9.54x10^{-7}$
ATAD2	2.17	4.11	36.23	$8.36x10^{-8}$	$1.09x10^{-6}$
PKHD1L1	-12.05	2.44	36.23	$8.38x10^{-8}$	$1.09x10^{-6}$
SLC8B1	-2.24	5.68	32.94	$2.54x10^{-7}$	$2.92x10^{-6}$
AMT	-2.21	5.28	30.36	$6.25x10^{-7}$	$6.50x10^{-6}$
QPCT	2.79	3.42	26.94	$2.15x10^{-6}$	$1.93x10^{-5}$
AHNAK	-3.55	10.54	23.46	$2.57x10^{-6}$	$2.24x10^{-5}$
BYSL	1.93	3.29	25.14	$4.19x10^{-6}$	$3.44x10^{-5}$
SPZ1	4.60	-4.61	23.14	$8.98x10^{-6}$	$6.72x10^{-5}$
FAM214A	-1.82	5.26	22.97	$9.59x10^{-6}$	$7.09x10^{-5}$
HOGA1	3.28	-2.05	22.92	$9.79x10^{-6}$	$7.21x10^{-5}$
DNAH14	1.69	1.56	22.86	$1.00x10^{-4}$	$7.37x10^{-5}$
KLHL23	2.33	4.41	22.81	$1.02x10^{-5}$	$7.48x10^{-5}$
KIAA1109	-1.55	6.89	22.70	$1.07x10^{-5}$	$7.77x10^{-5}$
TET1	2.54	0.10	22.28	1.25x10	$8.94x10^{-5}$
ANLN	2.80	3.49	22.05	$1.37x10^{-5}$	$9.66x10^{-5}$
MIB2	-1.94	5.76	22.02	$1.39x10^{-5}$	$9.76x10^{-5}$
NUDT6	-1.56	2.61	21.54	$1.68x10^{-5}$	$1.15x10^{-4}$
DCST1	-2.92	0.29	2.86	$2.20x10^{-5}$	$1.45x10^{-4}$

EPHB6	-3.75	2.78	20.09	$2.98x10^{-5}$	$1.89x10^{-4}$
NOD2	2.37	1.16	18.84	$4.95x10^{-5}$	$2.93x10^{-4}$
ARHGEF37	-3.29	4.92	17.29	$9.36x10^{-5}$	$5.07x10^{-4}$
KLF9	-3.13	6.29	15.91	$1.68x10^{-4}$	$8.43x10^{-4}$
FSTL4	2.19	-1.08	15.35	$2.13x10^{-4}$	$1.03x10^{-3}$
DGKQ	-1.71	6.44	14.94	$2.55x10^{-4}$	$1.21x10^{-3}$
MYO1H	2.24	-2.13	14.81	$2.70x10^{-4}$	$1.27x10^{-3}$
CECR2	-3.91	0.81	14.15	$3.59x10^{-4}$	$1.61x10^{-3}$
CSMD1	-7.34	-1.51	13.93	$3.95x10^{-4}$	$1.75x10^{-3}$
HTR3E	-9.46	0.38	12.92	$6.19x10^{-4}$	$2.58x10^{-3}$
FAM166A	1.64	3.27	12.84	$6.41x10^{-4}$	$2.66x10^{-3}$
SERPINH1	1.92	6.09	12.16	$8.68x10^{-4}$	$3.46x10^{-3}$
ASXL3	-3.32	2.32	11.83	$1.01x10^{-3}$	$3.93x10^{-3}$
EXOC3L4	-2.61	3.21	11.77	$1.04x10^{-3}$	$4.02x10^{-3}$
ZNF415	-2.07	2.46	11.51	$1.17x10^{-3}$	$4.47x10^{-3}$
GFI1B	-3.12	0.08	10.83	$1.60x10^{-3}$	$5.81x10^{-3}$
ITGA10	-5.07	3.69	10.74	$1.66x10^{-3}$	$6.02x10^{-3}$
PTPRM	-1.95	5.01	10.57	$1.80x10^{-3}$	$6.44x10^{-3}$
NLRP7	-7.58	-1.36	10.44	$1.92x10^{-3}$	$6.80x10^{-3}$
FOXN1	-7.88	-0.88	9.35	$3.21x10^{-3}$	$1.05x10^{-2}$
JPH3	-4.85	-0.04	9.32	$3.25x10^{-3}$	$1.06x10^{-2}$
CIDEC	-6.48	3.49	9.25	$3.36x10^{-3}$	$1.09x10^{-2}$
DUSP26	-3.39	0.67	9.15	$3.53x10^{-3}$	$1.14x10^{-2}$
ATG9B	1.57	1.33	8.35	$5.20x10^{-3}$	$1.57x10^{-2}$
VEPH1	-3.25	0.43	8.13	$5.79x10^{-3}$	$1.72x10^{-2}$
HYDIN	-4.56	0.09	7.99	$6.21x10^{-3}$	$1.83x10^{-2}$
KDM4D	1.69	-1.35	6.65	$1.21x10^{-2}$	$3.18x10^{-2}$
CHODL	1.59	0.23	6.54	$1.29x10^{-2}$	$3.33x10^{-2}$
CMYA5	-2.59	1.97	6.36	$1.41x10^{-2}$	$3.58x10^{-2}$
SYTL2	-1.59	7.55	6.24	$1.49x10^{-2}$	$3.76x10^{-2}$
GPRASP1	-2.85	4.42	6.13	$1.58x10^{-2}$	$3.94x10^{-2}$
GPR78	1.81	1.86	5.96	$1.73x10^{-2}$	$4.25x10^{-2}$
CST2	3.89	-3.46	5.94	$1.75x10^{-2}$	$4.29x10^{-2}$
ANO2	-1.82	0.23	5.93	$1.75x10^{-2}$	$4.30x10^{-2}$

Supplementary Table 16. Genes with rare germline variants identified in patient TCGA-D5-6929 using 'All vs One' approach. This table lists genes identified through the 'All vs One' approach that harbour rare germline variants in patient TCGA-D5-6929. Genes shown meet the differential expression threshold of |log2 fold change| ≥1.5 and FDR <5%. Variants were retained on filtering criteria for rare population frequency. logFC: log2 fold change in gene expression; logCPM: log2-transformed counts per million across all samples; F: quasi-likelihood F-test statistic; p-value: nominal p-value (uncorrected); FDR: p-value adjusted using Benjamini-Hochberg correction.

	logFC	logCPM	F	p-value	FDR
NOTUM	7.40	-1.17	186.84	$5.33x10^{-21}$	$4.37x10^{-18}$
SRPK1	2.28	6.57	74.90	$1.67x10^{-12}$	$1.52x10^{-1}$
TMEM97	2.19	4.51	39.62	$2.76x10^{-8}$	$7.28x10^{-7}$
LIFR	-5.60	4.88	35.48	$1.08x10^{-7}$	$2.39x10^{-6}$
MCM3	1.83	6.00	34.90	$1.31x10^{-7}$	$2.84x10^{-6}$
VWA2	4.09	0.92	31.52	$4.17x10^{-7}$	$7.67x10^{-6}$
GREB1L	3.47	-0.44	30.22	$6.57x10^{-7}$	$1.15x10^{-5}$
ATP11A	1.60	4.40	29.27	$9.21x10^{-7}$	$1.55x10^{-5}$
DZANK1	-2.15	1.50	28.67	$1.14X10^{-6}$	$1.85X10^{-5}$
UVSSA	-2.67	4.71	28.03	$1.44x10^{-6}$	$2.23x10^{-5}$
CDT1	2.45	3.45	23.72	$7.20x10^{-6}$	$8.81x10^{-5}$
ATOH8	-3.10	3.43	19.83	$3.31x10^{-5}$	$3.22x10^{-4}$
TIAM1	-1.87	2.74	15.66	$1.86x10^{-4}$	$1.35x10^{-3}$
BEX1	-5.10	0.40	12.91	$6.21x10^{-4}$	$3.65x10^{-3}$
SLC17A1	2.56	-2.72	12.38	$7.89x10^{-4}$	$4.44x10^{-3}$
ANK2	-4.92	4.44	12.17	$8.64x10^{-4}$	$4.79x10^{-3}$
CAMK4	-2.06	2.63	12.06	$9.12x10^{-4}$	$5.00x10^{-3}$
CDHR5	-2.47	8.75	11.03	$1.46x10^{-3}$	$7.30x10^{-3}$
RGL3	-2.26	3.43	10.01	$2.34x10^{-3}$	$1.08x10^{-2}$
COL12A1	2.54	6.04	9.51	$2.97x10^{-3}$	$1.31x10^{-2}$
LETM1	-1.64	6.90	9.18	$3.48x10^{-3}$	$1.49x10^{-2}$
COL11A2	-2.02	1.01	8.94	$3.90x10^{-3}$	$1.63x10^{-2}$
CYP3A7	-4.89	-0.94	8.10	$5.88x10^{-3}$	$2.25x10^{-2}$
HKDC1	2.30	4.60	7.90	$6.47x10^{-3}$	$2.44x10^{-2}$
CPZ	2.01	1.92	7.67	$7.28x10^{-3}$	$2.68x10^{-2}$
PROM1	-2.80	6.46	7.59	$7.57x10^{-3}$	$2.76x10^{-2}$
NOX5	-3.13	-1.50	7.58	$7.59x10^{-3}$	$2.77x10^{-2}$
HPDL	1.56	3.06	6.75	$1.16x10^{-2}$	$3.83x10^{-2}$
TRDN	-7.76	-1.99	6.65	$1.21x10^{-2}$	$3.99x10^{-2}$
HEPACAM2	-2.34	6.64	6.52	$1.30x10^{-2}$	$4.20x10^{-2}$
STRC	-2.05	-1.17	6.47	$1.33x10^{-2}$	$4.28x10^{-2}$

Supplementary Table 17. Genes with rare germline variants identified in patient TCGA-DC-6683 using 'All vs One' approach. This table lists genes identified through the 'All vs One' approach that harbour rare germline variants in patient TCGA-DC-6683. Genes shown meet the differential expression threshold of |log2 fold change| ≥1.5 and FDR <5%. Variants were retained on filtering criteria for rare population frequency. logFC: log2 fold change in gene expression; logCPM: log2-transformed counts per million across all samples; F: quasi-likelihood F-test statistic; p-value: nominal p-value (uncorrected); FDR: p-value adjusted using Benjamini-Hochberg correction.

	logFC	logCPM	F	p-value	FDR
SNTB1	2.98	4.01	223.44	$5.88x10^{-23}$	$3.57x10^{-20}$
KARS1	1.78	6.66	99.12	$8.21x10^{-15}$	$7.97x10^{-13}$
HM13	2.35	6.23	92.08	$3.53x10^{-14}$	$2.94x10^{-12}$
LZTR1	-2.06	6.20	74.11	$2.02x10^{-12}$	$1.14x10^{-10}$
MXRA5	3.38	4.36	63.90	$2.57x10^{-11}$	$1.15x10^{-9}$
ABHD2	2.14	6.92	61.99	$4.23x10^{-11}$	$1.82x10^{-9}$
TMEM97	2.37	4.53	50.69	$9.53x10^{-10}$	$2.82x10^{-8}$
TTLL3	-2.31	6.04	42.57	$1.09x10^{-8}$	$2.46x10^{-7}$
STAB1	-4.61	7.47	40.76	$1.92x10^{-8}$	$4.5x10^{-7}$
CCDC157	-1.88	2.00	36.64	$7.32x10^{-8}$	$1.32x10^{-6}$
CADM3	-4.90	4.19	28.22	$1.35x10^{-6}$	$1.67x10^{-5}$
ADK	1.75	3.75	26.05	$2.98x10^{-6}$	$3.32x10^{-5}$
RASGRP2	-4.46	4.30	18.10	$6.70x10^{-5}$	$4.86x10^{-4}$
FAT2	-3.29	0.68	16.40	$1.36x10^{-4}$	$8.88x10^{-4}$
LAMC3	-3.87	2.85	15.28	$2.19x10^{-4}$	$1.32x10^{-3}$
KIAA1549L	4.31	-0.09	15.15	$2.32x10^{-4}$	$1.39x10^{-3}$
CDH26	-2.57	-0.18	14.66	$2.87x10^{-4}$	$1.67x10^{-3}$
SYT2	-2.88	1.20	13.57	$4.64x10^{-4}$	$2.49x10^{-3}$
SDK1	-2.93	3.48	13.51	$4.76x10^{-4}$	$2.55x10^{-3}$
SLC7A2	-3.53	3.60	12.32	$8.08x10^{-4}$	$3.95x10^{-3}$
MCM2	1.66	4.47	11.99	$9.39x10^{-4}$	$4.46x10^{-3}$
GREB1L	-5.12	-0.68	11.09	$1.42x10^{-3}$	$6.26x10^{-3}$
VCAN	1.96	4.72	1.96	$1.51x10^{-3}$	$6.55x10^{-3}$
SHCBP1	2.03	2.03	10.85	$1.59x10^{-3}$	$6.83x10^{-3}$
HYAL1	-3.48	3.11	10.69	$1.70x10^{-3}$	$7.24x10^{-3}$
TTN	-1.91	4.37	10.38	$1.97x10^{-3}$	$8.19x10^{-3}$
CENPF	1.84	4.74	7.71	$7.12x10^{-3}$	$2.32x10^{-2}$
CMYA5	-2.90	1.97	7.61	$7.50x10^{-3}$	$2.42x10^{-2}$
PTPN22	-1.98	3.18	7.24	$9.00x10^{-3}$	$2.81x10^{-2}$
FSTL4	-2.27	-1.16	6.03	$1.67x10^{-2}$	$4.60x10^{-2}$
AJAP1	-2.41	-0.08	6.01	$1.68x10^{-2}$	$4.62x10^{-2}$
GLP1R	-5.91	-3.25	5.93	$1.76X10^{-2}$	$4.77x10^{-2}$

Supplementary Table 18. Genes with rare germline variants identified in patient 104 using 'All vs One' approach. This table lists genes identified through the 'All vs One' approach that harbour rare germline variants in patient 104. Genes shown meet the differential expression threshold of |log2 fold change| ≥1.5 and FDR <5%. Variants were retained on filtering criteria for rare population frequency. logFC: log2 fold change in gene expression; logCPM: log2-transformed counts per million across all samples; F: quasi-likelihood F-test statistic; p-value: nominal p-value (uncorrected); FDR: p-value adjusted using Benjamini-Hochberg correction.

	logFC	logCPM	F	p-value	FDR
TULP3	2.71	4.01	93.91	2.40×10^{-14}	1.96×10^{-11}
COL5A3	1.90	4.76	20.28	2.76×10^{-5}	1.20×10^{-3}
KIF14	2.66	2.11	16.74	1.18×10^{-4}	4.00×10^{-3}
LAMA5	2.66	7.22	15.88	1.70×10^{-4}	5.43×10^{-3}
MACC1	1.88	3.70	13.39	5.01×10^{-4}	1.28×10^{-2}
UNC93A	3.33	-1.59	13.35	5.10×10^{-4}	1.30×10^{-2}
FANCD2	1.55	3.70	12.47	7.57×10^{-4}	1.74×10^{-2}
CEL	2.53	1.60	11.85	9.99×10^{-4}	2.11×10^{-2}
CSF2	3.95	-4.14	11.24	1.32×10^{-3}	2.60×10^{-2}
CLEC5A	2.70	-1.75	10.10	2.25×10^{-3}	3.76×10^{-2}
MYOM2	-3.14	1.12	9.73	2.68×10^{-3}	4.26×10^{-2}
DNAH5	2.30	0.39	9.41	3.12×10^{-3}	4.77×10^{-2}

Supplementary Table 19. Genes with rare germline variants identified in patient 107 using 'All vs One' approach. This table lists genes identified through the 'All vs One' approach that harbour rare germline variants in patient 107. Genes shown meet the differential expression threshold of |log2 fold change| ≥1.5 and FDR <5%. Variants were retained on filtering criteria for rare population frequency. logFC: log2 fold change in gene expression; logCPM: log2-transformed counts per million across all samples; F: quasi-likelihood F-test statistic; p-value: nominal p-value (uncorrected); FDR: p-value adjusted using Benjamini-Hochberg correction.

	**logFC**	**logCPM**	**F**	**p-value**	**FDR**
OTOP1	10.96	-3.59	196.15	$1.60x10^{-21}$	$2.84x10^{-18}$
DNAH2	3.44	0.07	71.20	$4.09x10^{-12}$	$1.01x10^{-9}$
MUC16	5.58	-1.96	45.75	$4.11x10^{-9}$	$4.96x10^{-7}$
ACAN	4.87	-1.16	40.78	$1.91x10^{-8}$	$2.02x10^{-6}$
COL18A1	2.21	6.51	29.04	$1.00x10^{-6}$	$6.76x10^{-5}$
BMP4	2.26	4.22	25.67	$3.45x10^{-6}$	$1.94x10^{-4}$
DNAH14	1.79	1.58	24.18	$6.03x10^{-6}$	$3.20x10^{-4}$
SLC16A4	2.68	1.19	22.90	$9.87x10^{-6}$	$4.86x10^{-4}$
TNK2	1.61	5.34	19.64	$3.58x10^{-5}$	$1.41x10^{-3}$
DMXL2	-1.55	5.85	19.24	$4.21x10^{-5}$	$1.62x10^{-3}$
THNSL2	-6.87	4.30	17.32	$9.26x10^{-5}$	$3.01x10^{-3}$
ADAMTS18	2.72	-1.80	11.95	$9.58x10^{-4}$	$1.83x10^{-2}$

Supplementary Table 20. Genes with rare germline variants identified in patient 108 using 'All vs One' approach. This table lists genes identified through the 'All vs One' approach that harbour rare germline variants in patient 108. Genes shown meet the differential expression threshold of |log2 fold change| ≥1.5 and FDR <5%. Variants were retained on filtering criteria for rare population frequency. logFC: log2 fold change in gene expression; logCPM: log2-transformed counts per million across all samples; F: quasi-likelihood F-test statistic; p-value: nominal p-value (uncorrected); FDR: p-value adjusted using Benjamini-Hochberg correction.

	logFC	logCPM	F	p-value	FDR
CELSR3	2.90	2.58	122.21	$1.03x10^{-16}$	$8.08x10^{-14}$
ADGRF5	2.33	3.97	39.07	$3.31x10^{-8}$	$2.53x10^{-6}$
NR3C2	-2.77	6.28	36.32	$8.12x10^{-8}$	$5.33x10^{-6}$
SPIB	-6.08	5.80	30.68	$5.58x10^{-7}$	$2.80x10^{-5}$
SH3PXD2B	2.01	5.17	29.93	$7.29x10^{-7}$	$3.45x10^{-5}$
MMS22L	1.75	2.85	26.86	$2.21x10^{-6}$	$8.62x10^{-5}$
CENPQ	1.93	1.61	26.10	$2.93x10^{-6}$	$1.09x10^{-4}$
SLC7A5	3.43	4.33	24.73	$4.90x10^{-6}$	$1.64x10^{-4}$
SLC4A4	-4.18	7.69	24.16	$6.08x10^{-6}$	$1.96x10^{-4}$
ADAMTS2	2.41	4.14	22.57	$1.12x10^{-5}$	$3.18x10^{-4}$
IL17RC	-1.76	5.10	20.46	$2.57x10^{-5}$	$6.03x10^{-4}$
FANCD2	1.85	3.71	18.72	$5.20x10^{-5}$	$1.06x10^{-3}$
OPTN	-1.67	6.66	17.57	$8.35x10^{-5}$	$1.52x10^{-3}$
PNPLA7	-2.84	5.18	17.05	$1.04x10^{-4}$	$1.79x10^{-3}$
SIGLEC1	-3.10	4.91	16.95	$1.08x10^{-4}$	$1.84x10^{-3}$
NRAP	-4.76	1.18	14.65	$2.89x10^{-4}$	$3.91x10^{-3}$
TCEA3	-1.71	6.96	13.94	$3.94x10^{-4}$	$4.94x10^{-3}$
IL2RG	-3.06	6.39	13.69	$4.40x10^{-4}$	$5.37x10^{-3}$
POLE2	1.85	1.86	13.54	$4.69x10^{-4}$	$5.62x10^{-3}$
LRRC66	-2.88	3.79	13.52	$4.74x10^{-4}$	$5.66x10^{-3}$
CTH	-1.85	2.85	12.69	$6.84x10^{-4}$	$7.51x10^{-3}$
GRAMD2B	-1.52	5.57	10.75	$1.66x10^{-3}$	$1.41x10^{-2}$
TTLL6	-2.50	4.11	9.48	$3.01x10^{-3}$	$2.19x10^{-2}$
TRPM4	-1.51	8.11	9.43	$3.09x10^{-3}$	$2.24x10^{-2}$
SLIT1	-2.95	-0.08	7.56	$7.69x10^{-3}$	$4.33x10^{-2}$
TMEM54	-1.66	8.11	7.31	$8.71x10^{-3}$	$4.73x10^{-2}$
EXOC3L4	-1.99	3.21	7.31	$8.72x10^{-3}$	$4.73x10^{-2}$
ABCB11	-5.32	2.75	7.17	$9.32x10^{-2}$	$4.96x10^{-2}$

Supplementary Table 21. Genes with rare germline variants identified in patient TCGA-CK-4947 using 'All vs One' approach. This table lists genes identified through the 'All vs One' approach that harbour rare germline variants in patient TCGA-CK-4947. Genes shown meet the differential expression threshold of |log2 fold change| ≥1.5 and FDR <5%. Variants were retained on filtering criteria for rare population frequency. logFC: log2 fold change in gene expression; logCPM: log2-transformed counts per million across all samples; F: quasi-likelihood F-test statistic; p-value: nominal p-value (uncorrected); FDR: p-value adjusted using Benjamini-Hochberg correction.

	logFC	logCPM	F	p-value	FDR
LAMP1	1.71	7.61	126.76	$4.62x10^{-17}$	$4.20x10^{-14}$
ROBO2	4.55	0.79	72.13	$3.26x10^{-12}$	$4.94x10^{-10}$
LIN9	2.01	2.17	40.35	$2.19x10^{-8}$	$8.11x10^{-7}$
PKDCC	-3.19	5.97	38.89	$3.50x10^{-8}$	$1.18x10^{-6}$
WDHD1	2.46	2.78	33.03	$2.47x10^{-7}$	$5.93x10^{-6}$
NEIL1	-2.46	4.48	32.47	$2.99x10^{-7}$	$6.95x10^{-6}$
LGI4	-5.68	5.31	32.02	$3.50x10^{-7}$	$7.99x10^{-6}$
ZC3H12C	-2.79	4.59	29.74	$7.78x10^{-7}$	$1.58x10^{-5}$
CACNB2	-3.14	4.09	27.83	$1.55x10^{-6}$	$2.77x10^{-5}$
GIGYF1	-1.59	6.50	25.52	$3.64x10^{-6}$	$5.53x10^{-5}$
TRPV1	-2.92	4.07	23.00	$9.47x10^{-6}$	$1.24x10^{-4}$
CDK3	-3.97	4.11	22.81	$1.02x10^{-5}$	$1.32x10^{-4}$
ADCY6	-1.51	7.65	20.52	$2.51x10^{-5}$	$2.77x10^{-4}$
RGPD3	-7.08	-2.14	16.92	$1.09x10^{-4}$	$9.27x10^{-4}$
MROH7	-2.91	4.34	16.74	1.18x10-4	$9.87x10^{-4}$
LAPTM4B	1.51	5.51	16.73	1.19x10-4	$9.90x10^{-4}$
CFTR	1.51	7.57	15.91	$1.68x10^{-4}$	$1.31x10^{-3}$
NUTM1	-4.52	-0.89	15.63	$1.89x10^{-4}$	$1.45x10^{-3}$
KLF15	-5.25	3.08	15.31	$2.17x10^{-4}$	$1.61x10^{-3}$
SLC16A11	-3.34	0.05	14.54	$3.02x10^{-4}$	$2.11x10^{-3}$
ASPM	2.20	3.52	13.95	$3.92x10^{-4}$	$2.59x10^{-3}$
KNL1	2.11	3.22	13.30	$5.21x10^{-4}$	$3.25x10^{-3}$
TTN	-2.23	4.37	13.25	$5.33x10^{-4}$	$3.31x10^{-3}$
MS4A12	-6.91	7.81	12.31	$8.11x10^{-4}$	$4.64x10^{-3}$
AZIN2	-2.82	3.85	12.22	$8.47x10^{-4}$	$4.80x10^{-3}$
GRIN1	-3.31	3.11	12.21	$8.50x10^{-4}$	$4.81x10^{-3}$
ZNF285	-2.53	0.81	11.21	$1.34x10^{-3}$	$7.01x10^{-3}$
CMYA5	-3.48	1.96	10.13	$2.22x10^{-3}$	$1.04x10^{-2}$
AKR1C3	-4.14	7.68	10.09	$2.26x10^{-3}$	$1.06x10^{-2}$
CATSPERE	-2.38	-1.07	9.50	$2.99x10^{-3}$	$1.33x10^{-2}$
KLK4	-5.42	0.78	9.23	$3.39x10^{-3}$	$1.47x10^{-2}$
SLC4A10	-4.24	2.60	7.63	$7.41x10^{-3}$	$2.73x10^{-2}$
FTCD	-4.47	-1.07	7.10	$9.65x10^{-3}$	$3.37x10^{-2}$
CCN3	-2.22	2.70	6.85	$1.10x10^{-2}$	$3.72x10^{-2}$
RCVRN	-3.66	-2.20	6.82	$1.11x10^{-2}$	$3.76x10^{-2}$
CCR5	1.71	2.08	6.81	$1.12x10^{-2}$	$3.78x10^{-2}$
EFS	-2.36	3.30	6.37	$1.40x10^{-2}$	$4.49x10^{-2}$

Supplementary Table 22. Genes with rare germline variants identified in patient TCGA-EI-6508 using 'All vs One' approach. This table lists genes identified through the 'All vs One' approach that harbour rare germline variants in patient TCGA-EI-6508. Genes shown meet the differential expression threshold of |log2 fold change| ≥1.5 and FDR <5%. Variants were retained on filtering criteria for rare population frequency. logFC: log2 fold change in gene expression; logCPM: log2-transformed counts per million across all samples; F: quasi-likelihood F-test statistic; p-value: nominal p-value (uncorrected); FDR: p-value adjusted using Benjamini-Hochberg correction.

	logFC	logCPM	F	p-value	FDR
SYCP2	2.09	1.19	42.59	$1.08x10^{-8}$	$3.30x10^{-7}$
PKDCC	-3.23	5.98	39.62	$2.77x10^{-8}$	$7.25x10^{-7}$
STK31	3.33	-0.44	38.92	$3.46x10^{-8}$	$8.76x10^{-7}$
ATP13A3	1.78	6.56	27.38	$1.83x10^{-6}$	$2.47x10^{-5}$
TGM1	-2.94	0.54	27.13	$2.01x10^{-6}$	$2.68x10^{-5}$
ADCY4	-2.51	4.05	25.97	$3.08x10^{-6}$	$3.83x10^{-5}$
L1TD1	4.50	3.67	24.96	$4.49x10^{-6}$	$5.27x10^{-5}$
PGM2L1	1.66	4.14	24.20	$5.98x10^{-6}$	$6.82x10^{-5}$
SLFN13	-3.41	3.80	21.94	$1.43x10^{-5}$	$1.43x10^{-4}$
KIF26A	2.03	3.58	19.74	$3.44x10^{-5}$	$3.04x10^{-4}$
CNTNAP2	-3.97	2.64	18.68	$5.27x10^{-5}$	$4.38x10^{-4}$
SORCS1	-5.73	2.81	16.94	$1.08x10^{-4}$	$8.06x10^{-4}$
DCLK3	2.51	-1.93	16.64	$1.23x10^{-4}$	$8.98x10^{-4}$
KCTD1	-2.21	2.79	16.38	$1.37x10^{-4}$	$9.82x10^{-4}$
EPHX2	-2.17	6.57	15.92	$1.67x10^{-4}$	$1.16x10^{-3}$
FBN2	1.86	2.11	15.23	$2.24x10^{-4}$	$1.48x10^{-3}$
MAMDC4	-2.24	5.47	14.69	$2.83x10^{-4}$	$1.80x10^{-3}$
ZFYVE28	-2.13	4.91	13.78	$4.23x10^{-4}$	$2.53x10^{-3}$
IQGAP3	2.05	4.46	13.01	$5.92x10^{-4}$	$3.35x10^{-3}$
SHANK3	-1.94	4.76	12.99	$6.00x10^{-4}$	$3.38x10^{-3}$
TRPM2	1.53	2.97	11.77	$1.04x10^{-3}$	$5.33x10^{-3}$
KIF18B	2.19	3.21	11.71	$1.07x10^{-3}$	$5.45x10^{-3}$
TTN	-1.81	4.37	9.39	$3.15x10^{-3}$	$1.32x10^{-2}$
TMEM150B	1.99	3.00	9.10	$3.61x10^{-3}$	$1.47x10^{-2}$
VEPH1	-3.34	0.43	8.66	$4.48x10^{-3}$	$1.74x10^{-2}$
COL3A1	1.54	8.94	8.57	$4.68x10^{-3}$	$1.81x10^{-2}$
GRIN1	-2.56	3.11	8.29	$5.35x10^{-3}$	$2.01x10^{-2}$
STAT4	-1.73	2.22	7.19	$9.23x10^{-3}$	$3.11x10^{-2}$
VCAN	1.62	4.70	6.83	$1.11x10^{-2}$	$3.58x10^{-2}$
CCDC60	-3.77	0.09	6.02	$1.68x10^{-2}$	$4.97x10^{-2}$

Supplementary Table 23. Genes with rare germline variants identified in patient 102 using 'All vs One' approach. This table lists genes identified through the 'All vs One' approach that harbour rare germline variants in patient 102. Genes shown meet the differential expression threshold of |log2 fold change| ≥1.5 and FDR <5%. Variants were retained on filtering criteria for rare population frequency. logFC: log2 fold change in gene expression; logCPM: log2-transformed counts per million across all samples; F: quasi-likelihood F-test statistic; p-value: nominal p-value (uncorrected); FDR: p-value adjusted using Benjamini-Hochberg correction.

	logFC	logCPM	F	p-value	FDR
ESM1	8.77	-0.92	135.42	1.06×10^{-17}	1.14×10^{-14}
MMP7	8.75	1.11	84.36	1.89×10^{-13}	6.49×10^{-11}
NOTCH3	3.77	5.10	63.63	2.76×10^{-11}	5.28×10^{-9}
VWF	3.01	6.78	56.83	1.69×10^{-10}	2.58×10^{-8}
AFAP1L1	2.82	2.32	49.45	1.36×10^{-9}	1.59×10^{-7}
CEP164	1.53	4.33	42.60	1.08×10^{-8}	9.45×10^{-7}
ABCC2	2.53	0.91	37.07	6.34×10^{-8}	4.14×10^{-6}
SLC22A12	5.57	-4.27	32.94	2.55×10^{-7}	1.38×10^{-5}
PTPRS	2.54	5.02	28.06	1.43×10^{-6}	5.88×10^{-5}
CACNA1A	-3.94	1.96	24.40	5.56×10^{-6}	1.84×10^{-4}
CSMD2	2.69	-0.44	23.69	7.58×10^{-6}	2.32×10^{-4}
MROH7	-3.60	4.34	22.24	1.28×10^{-5}	3.67×10^{-4}
LMCD1	2.16	2.76	20.87	2.19×10^{-5}	5.74×10^{-4}
INSC	1.82	1.86	18.77	5.09×10^{-5}	1.15×10^{-3}
MGAT4B	-2.22	8.50	16.24	1.46×10^{-4}	2.74×10^{-3}
GPR78	2.75	1.80	14.59	2.96×10^{-4}	4.73×10^{-3}
DQX1	-2.42	5.07	14.11	3.65×10^{-4}	5.58×10^{-3}
NINL	2.26	3.23	14.10	3.67×10^{-4}	5.60×10^{-3}
MFGE8	1.69	6.44	14.09	3.68×10^{-4}	5.60×10^{-3}
PDZD7	1.57	0.48	12.11	8.89×10^{-4}	1.11×10^{-2}
SLC28A1	3.89	-3.69	11.96	9.51×10^{-4}	1.16×10^{-2}
UBAP1L	-2.42	0.12	11.87	9.90×10^{-4}	1.20×10^{-2}
UGT1A4	-3.53	6.15	11.73	1.06×10^{-3}	1.26×10^{-2}
ADRB1	-2.50	1.02	10.17	2.18×10^{-3}	2.19×10^{-2}
BFSP1	1.64	-0.43	10.16	2.18×10^{-3}	2.19×10^{-2}
PDZD2	1.60	2.58	10.02	2.34×10^{-3}	2.31×10^{-2}
HAL	1.68	0.33	9.53	2.94×10^{-3}	2.72×10^{-2}
ITGAX	1.71	3.07	7.98	6.24×10^{-3}	4.60×10^{-2}

Supplementary Table 24. Genes with rare germline variants identified in patient 209 using 'All vs One' approach. This table lists genes identified through the 'All vs One' approach that harbour rare germline variants in patient 209. Genes shown meet the differential expression threshold of |log2 fold change| ≥1.5 and FDR <5%. Variants were retained on filtering criteria for rare population frequency. logFC: log2 fold change in gene expression; logCPM: log2-transformed counts per million across all samples; F: quasi-likelihood F-test statistic; p-value: nominal p-value (uncorrected); FDR: p-value adjusted using Benjamini-Hochberg correction.

	logFC	logCPM	F	p-value	FDR
ADAMTS2	4.31	4.33	51.83	6.85×10^{-10}	2.80×10^{-7}
BICC1	2.63	2.81	39.68	2.72×10^{-8}	5.55×10^{-6}
LRRC32	2.65	4.92	34.97	1.28×10^{-7}	1.85×10^{-5}
ACAN	6.27	-0.40	34.88	1.31×10^{-7}	1.89×10^{-5}
P4HA3	2.75	0.65	29.23	9.35×10^{-7}	9.07×10^{-5}
COL5A1	3.32	6.68	23.47	7.93×10^{-6}	4.82×10^{-4}
MYO1A	-2.52	7.77	22.60	1.11×10^{-5}	6.39×10^{-4}
GPAT3	-3.66	4.87	17.59	8.26×10^{-5}	3.01×10^{-3}
COL2A1	-5.56	0.74	17.42	8.88×10^{-5}	3.19×10^{-3}
MYO7A	1.61	2.96	14.46	3.13×10^{-4}	8.24×10^{-3}
PIEZO1	1.54	7.05	14.31	3.34×10^{-4}	8.69×10^{-3}
ITGAM	2.06	2.82	12.72	6.77×10^{-4}	1.45×10^{-2}
SFRP1	-4.59	4.31	11.94	9.63×10^{-4}	1.85×10^{-2}
ABCA9	-3.14	2.92	10.43	1.93×10^{-3}	2.97×10^{-2}
PTPRO	2.06	4.54	9.12	3.59×10^{-3}	4.54×10^{-2}

Supplementary Table 25. Genes with rare germline variants identified in patient 302 using ‘All vs One’ approach. This table lists genes identified through the ‘All vs One’ approach that harbour rare germline variants in patient 302. Genes shown meet the differential expression threshold of |log2 fold change| ≥1.5 and FDR <5%. Variants were retained on filtering criteria for rare population frequency. logFC: log2 fold change in gene expression; logCPM: log2-transformed counts per million across all samples; F: quasi-likelihood F-test statistic; p-value: nominal p-value (uncorrected); FDR: p-value adjusted using Benjamini-Hochberg correction.

	logFC	logCPM	F	p-value	FDR
HEPHL1	6.69	-0.37	123.18	8.68×10^{-17}	6.16x10-14
ROBO4	3.29	3.54	118.96	1.85×10^{-16}	1.15×10^{-13}
PALD1	3.52	3.54	98.20	9.92×10^{-15}	3.20×10^{-12}
SPARC	3.35	7.88	85.73	1.40×10^{-13}	3.03×10^{-22}
INSC	2.51	1.88	42.38	1.16×10^{-8}	7.20×10^{-7}
SORD	1.88	4.29	29.31	9.09×10^{-7}	3.51×10^{-5}
ITGA11	2.29	2.22	26.66	2.38×10^{-6}	8.18×10^{-5}
TOX	-2.15	4.30	26.65	2.39×10^{-6}	8.19×10^{-5}
TP73	1.95	-0.14	23.32	8.39×10^{-6}	2.41×10^{-4}
MUC16	4.28	2.32	22.43	1.18×10^{-5}	3.22×10^{-4}
CFAP46	3.36	-2.11	21.72	1.56×10^{-5}	4.06×10^{-4}
COL27A1	2.01	4.41	18.94	4.75×10^{-5}	1.04×10^{-3}
PLEKHO2	1.89	4.68	18.15	6.58×10^{-5}	1.37×10^{-3}
ADGRV1	-2.29	1.68	16.27	1.44×10^{-4}	2.61×10^{-3}
COL15A1	3.17	5.39	15.67	1.86×10^{-4}	3.20×10^{-3}
DOP1B	-1.55	6.24	12.67	6.91×10^{-4}	8.98×10^{-3}
FN1	3.26	8.65	12.26	8.30×10^{-4}	1.04×10^{-2}
SH3TC2	2.04	0.54	11.65	1.09×10^{-3}	1.29×10^{-2}
SULF1	2.82	4.74	10.12	2.23×10^{-3}	2.15×10^{-2}
CHI3L2	2.66	0.82	8.82	4.13×10^{-3}	3.39×10^{-2}
SACS	1.78	3.64	8.38	5.12×10^{-3}	3.97×10^{-2}

Supplementary Table 26. Genes with rare germline variants identified in patient 304 using 'All vs One' approach. This table lists genes identified through the 'All vs One' approach that harbour rare germline variants in patient 304. Genes shown meet the differential expression threshold of |log2 fold change| ≥1.5 and FDR <5%. Variants were retained on filtering criteria for rare population frequency. logFC: log2 fold change in gene expression; logCPM: log2-transformed counts per million across all samples; F: quasi-likelihood F-test statistic; p-value: nominal p-value (uncorrected); FDR: p-value adjusted using Benjamini-Hochberg correction.

	logFC	logCPM	F	p-value	FDR
THBS2	8.46	4.45	153.41	$6.11x10^{-19}$	$2.71x10^{-16}$
TFR2	3.69	-0.13	116.09	$3.11x10^{-16}$	$8.59x10^{-14}$
SYCP2	3.15	1.20	110.94	$8.11x10^{-16}$	$2.03x10^{-13}$
MET	3.13	5.74	89.03	$6.79x10^{-14}$	$9.70x10^{-12}$
LRRC32	3.13	4.90	80.86	$4.17x10^{-13}$	$4.96x10^{-11}$
KIAA1549	2.61	2.65	79.28	$6.00x10^{-13}$	$6.65x10^{-11}$
FST	4.54	2.96	57.44	$1.43x10^{-10}$	$1.02x10^{-8}$
AEBP1	3.47	7.03	55.51	$2.43x10^{-10}$	$1.67x10^{-8}$
PPL	2.86	4.85	53.01	$4.91x10^{-10}$	$3.16x10^{-8}$
ESAM	1.85	4.27	45.82	$4.02x10^{-9}$	$2.04x10^{-7}$
CEP170	2.21	3.37	42.87	$9.93x10^{-9}$	$4.68x10^{-7}$
DOK7	3.38	0.01	32.89	$2.59x10^{-7}$	$9.40x10^{-6}$
GLI2	3.53	2.32	32.69	$2.78x10^{-7}$	$9.95x10^{-6}$
MPO	4.25	-2.56	25.28	$3.99x10^{-6}$	$1.10x10^{-4}$
ALPK3	4.43	3.50	23.70	$7.25x10^{-6}$	$1.88x10^{-4}$
RP1L1	3.95	-2.65	20.41	$2.62x10^{-5}$	$5.82x10^{-4}$
TNC	5.04	6.94	18.91	$4.80x10^{-5}$	$9.73x10^{-4}$
HECTD3	-1.62	7.03	18.54	$5.60x10^{-5}$	$1.12x10^{-3}$
TRABD2A	-2.20	6.42	16.35	$1.39x10^{-4}$	$2.38x10^{-3}$
LRRC17	2.39	0.43	15.62	$1.90x10^{-4}$	$3.09x10^{-3}$
ANKLE1	1.85	-0.18	11.00	$1.48x10^{-3}$	$1.55x10^{-2}$
LAMC1	1.64	7.21	10.91	$1.54x10^{-3}$	$1.61x10^{-2}$
PCDHB14	1.67	1.59	10.79	$1.63x10^{-3}$	$1.68x10^{-2}$
ABCA10	-2.08	3.03	9.79	$2.61x10^{-3}$	$2.39x10^{-2}$
PDE6A	-3.50	3.31	9.56	$2.90x10^{-3}$	$2.59x10^{-2}$
CCDC68	-1.62	5.49	8.19	$5.62x10^{-3}$	$4.14x10^{-2}$

Supplementary Table 27. Genes with rare germline variants identified in patient 305 using 'All vs One' approach. This table lists genes identified through the 'All vs One' approach that harbour rare germline variants in patient 305. Genes shown meet the differential expression threshold of |log2 fold change| ≥1.5 and FDR <5%. Variants were retained on filtering criteria for rare population frequency. logFC: log2 fold change in gene expression; logCPM: log2-transformed counts per million across all samples; F: quasi-likelihood F-test statistic; p-value: nominal p-value (uncorrected); FDR: p-value adjusted using Benjamini-Hochberg correction.

	logFC	logCPM	F	p-value	FDR
APLN	5.99	0.39	60.05	$7.08x10^{-11}$	$1.01x10^{-8}$
VTCN1	6.50	-4.29	45.24	$4.79x10^{-9}$	$4.45x10^{-7}$
PLEKHG4	2.39	4.28	33.10	$2.40x10^{-7}$	$1.32x10^{-5}$
PLVAP	1.89	6.51	27.63	$1.67x10^{-6}$	$6.74x10^{-5}$
VWA5B2	-5.81	1.61	26.52	$2.51x10^{-6}$	$9.34x10^{-5}$
PCED1A	1.57	5.26	23.47	$7.93x10^{-6}$	$2.40x10^{-4}$
NTRK2	-7.11	4.18	23.37	$8.23x10^{-6}$	$2.47x10^{-4}$
LYPD3	2.01	0.17	22.91	$9.84x10^{-6}$	$2.86x10^{-4}$
SLC19A1	1.97	5.39	20.44	$2.59x10^{-5}$	$6.41x10^{-4}$
PLD1	-2.25	5.71	18.79	$5.05x10^{-5}$	$1.11x10^{-3}$
PYGO1	3.33	1.95	17.49	$8.62x10^{-5}$	$1.69x10^{-3}$
ACSS2	-1.70	7.97	12.34	$8.02x10^{-4}$	$1.00x10^{-2}$
FAM227A	2.10	0.24	12.13	$8.81x10^{-4}$	$1.08x10^{-2}$
SCN5A	1.72	0.06	11.22	$1.34x10^{-3}$	$1.47x10^{-2}$
TRPM4	-1.53	8.11	9.75	$2.66x10^{-3}$	$2.45x10^{-2}$
FAM110C	-2.23	4.98	9.73	$2.68x10^{-3}$	$2.46x10^{-2}$
INSL3	-2.25	-1.37	7.90	$6.48x10^{-3}$	$4.61x10^{-2}$

Supplementary Table 28. Genes with rare germline variants identified in patient 308 using 'All vs One' approach. This table lists genes identified through the 'All vs One' approach that harbour rare germline variants in patient 308. Genes shown meet the differential expression threshold of |log2 fold change| ≥1.5 and FDR <5%. Variants were retained on filtering criteria for rare population frequency. logFC: log2 fold change in gene expression; logCPM: log2-transformed counts per million across all samples; F: quasi-likelihood F-test statistic; p-value: nominal p-value (uncorrected); FDR: p-value adjusted using Benjamini-Hochberg correction.

	logFC	logCPM	F	p-value	FDR
ACAN	5.90	-1.00	66.51	$1.32x10^{-11}$	$3.42x10^{-9}$
KCNT1	4.47	-0.93	51.27	$8.06x10^{-10}$	$1.18x10^{-7}$
CSMD2	3.21	-0.42	37.71	$5.15x10^{-8}$	$4.60x10^{-6}$
DNAH10	2.12	-0.02	17.39	$9.01x10^{-5}$	$2.68x10^{-3}$
DCST2	-3.01	1.80	13.52	$4.73x10^{-4}$	$1.00x10^{-2}$
ECEL1	3.75	-1.69	13.02	$5.91x10^{-4}$	$1.19x10^{-2}$
ADGRV1	-1.82	1.68	11.18	$1.36x10^{-3}$	$2.20x10^{-2}$
LRRC70	1.59	0.45	10.91	$1.54x10^{-3}$	$2.41x10^{-2}$
CPA6	-2.69	1.83	10.68	$1.71x10^{-3}$	$2.59x10^{-2}$
BRINP3	-3.39	3.00	9.87	$2.51x10^{-3}$	$3.38x10^{-2}$
LRRC8E	1.86	1.06	9.14	$3.55x10^{-3}$	$4.31x10^{-2}$
NFE2	2.92	-1.57	8.69	$4.40x10^{-3}$	$4.96x10^{-2}$

Supplementary Table 29. Genes with rare germline variants identified in patient TCGA-AZ-4323 using 'All vs One' approach. This table lists genes identified through the 'All vs One' approach that harbour rare germline variants in patient TCGA-AZ-4323. Genes shown meet the differential expression threshold of |log2 fold change| ≥1.5 and FDR <5%. Variants were retained on filtering criteria for rare population frequency. logFC: log2 fold change in gene expression; logCPM: log2-transformed counts per million across all samples; F: quasi-likelihood F-test statistic; p-value: nominal p-value (uncorrected); FDR: p-value adjusted using Benjamini-Hochberg correction.

	logFC	logCPM	F	p-value	FDR
CHRNA4	8.07	-1.28	205.34	$5.01x10^{-22}$	$1.31x10^{-19}$
BMP7	4.63	2.25	104.45	$2.82x10^{-15}$	$2.43x10^{-13}$
ADAMTS20	6.76	-4.27	92.96	$4.63x10^{-14}$	$3.29x10^{-12}$
RNFT2	2.94	0.14	48.82	$1.64x10^{-9}$	$5.24x10^{-8}$
SEZ6L2	2.62	4.10	47.42	$2.48x10^{-9}$	$7.58x10^{-8}$
MYO3A	3.92	-0.23	38.80	$3.60x10^{-8}$	$8.70x10^{-7}$
WDR72	4.81	1.13	37.49	$5.51x10^{-8}$	$1.28x10^{-6}$
TRPM3	3.26	-1.32	34.53	$1.48x10^{-7}$	$3.03x10^{-6}$
PTPRC	2.71	5.43	27.76	$1.59x10^{-6}$	$2.47x10^{-5}$
CWH43	-4.34	5.01	23.34	$8.32x10^{-6}$	$1.02x10^{-4}$
CPNE9	3.18	-2.14	21.81	$1.51x10^{-5}$	$1.71x10^{-4}$
FAM162A	-1.78	6.97	21.20	$1.91x10^{-5}$	$2.10x10^{-4}$
CBARP	2.06	0.25	20.06	$3.02x10^{-5}$	$3.10x10^{-4}$
ZFR2	3.05	-1.86	19.82	$3.32x10^{-5}$	$3.34x10^{-4}$
MUC16	3.59	-2.34	18.15	$6.56x10^{-5}$	$5.96x10^{-4}$
NAPEPLD	-2.21	5.59	15.89	$1.69x10^{-4}$	$1.31x10^{-3}$
TAL1	1.71	1.91	15.51	$1.99x10^{-4}$	$1.50x10^{-3}$
GPRIN1	2.45	2.34	14.40	$3.21x10^{-4}$	$2.21x10^{-3}$
CELSR1	1.95	3.53	14.20	$3.51x10^{-4}$	$2.37x10^{-3}$
CNNM1	1.78	0.38	13.74	$4.30x10^{-4}$	$2.81x10^{-3}$
ST14	-1.77	9.09	13.01	$5.93x10^{-4}$	$3.64x10^{-3}$
SHROOM3	-1.60	7.51	12.84	$6.39x10^{-4}$	$3.87x10^{-3}$
PPP1R14D	-2.36	5.62	12.19	$8.57x10^{-4}$	$4.96x10^{-3}$
MYH10	1.93	4.23	11.29	$1.30x10^{-3}$	$6.86x10^{-3}$
PANK3	-1.50	7.49	11.16	$1.38x10^{-3}$	$7.20x10^{-3}$
ACACB	-1.98	6.31	11.11	$1.40x10^{-3}$	$7.31x10^{-3}$
SCIN	-2.88	5.85	10.29	$2.06x10^{-3}$	$9.95x10^{-3}$
COL22A1	3.35	-0.10	9.21	$3.43x10^{-3}$	$1.50x10^{-2}$
STX19	-2.10	3.42	8.72	$4.34x10^{-3}$	$1.81x10^{-2}$
FOLH1	2.73	-0.74	7.84	$6.67x10^{-3}$	$2.56x10^{-2}$
CDHR2	-2.84	6.70	6.62	$1.23x10^{-2}$	$4.18x10^{-2}$
COL28A1	-2.27	3.29	6.59	$1.25x10^{-2}$	$4.22x10^{-2}$

Supplementary Table 30. Genes with rare germline variants identified in patient TCGA-AZ-5403 using 'All vs One' approach. This table lists genes identified through the 'All vs One' approach that harbour rare germline variants in patient TCGA-AZ-5403. Genes shown meet the differential expression threshold of |log2 fold change| ≥1.5 and FDR <5%. Variants were retained on filtering criteria for rare population frequency. logFC: log2 fold change in gene expression; logCPM: log2-transformed counts per million across all samples; F: quasi-likelihood F-test statistic; p-value: nominal p-value (uncorrected); FDR: p-value adjusted using Benjamini-Hochberg correction.

	logFC	logCPM	F	p-value	FDR
NELFCD	2.42	5.62	229.12	$3.07x10^{-23}$	$3.63x10^{-20}$
GLS2	2.71	0.69	90.70	$4.74x10^{-14}$	$3.44x10^{-12}$
EIF3I	1.90	6.84	66.99	$1.17x10^{-11}$	$4.56x10^{-10}$
ZSWIM8	-2.67	6.54	61.67	$4.61x10^{-11}$	$1.57x10^{-9}$
ITGA11	2.90	2.34	55.86	$2.21x10^{-10}$	$6.15x10^{-9}$
NDST2	-2.00	4.94	45.47	$4.46x10^{-9}$	$8.74x10^{-8}$
DDX18	1.54	6.04	43.32	$8.63x10^{-9}$	$1.55x10^{-7}$
DAB2IP	-1.64	7.38	39.51	$2.87x10^{-8}$	$4.46x10^{-7}$
DNAH17	-2.04	2.07	34.93	$1.29x10^{-7}$	$1.69x10^{-6}$
EXD3	-2.13	4.80	33.37	$2.19x10^{-7}$	$2.69x10^{-6}$
RUBCNL	2.55	4.91	32.13	$3.36x10^{-7}$	$3.92x10^{-6}$
MUC4	-4.95	8.48	30.22	$6.58x10^{-7}$	$7.10x10^{-6}$
OGA	-1.71	7.40	30.20	$6.61x10^{-7}$	$7.12x10^{-6}$
TTN	-3.57	4.37	27.51	$1.74x10^{-6}$	$1.67x10^{-5}$
CCDC57	-1.89	5.04	27.33	$1.86x10^{-6}$	$1.77x10^{-5}$
NRAS	1.56	6.08	24.72	$4.92x10^{-6}$	$4.17x10^{-5}$
CCDC17	-3.88	1.51	23.77	$7.05x10^{-6}$	$5.72x10^{-5}$
FRMD4A	-1.79	4.38	22.59	$1.11x10^{-5}$	$8.57x10^{-5}$
EXOC3L4	-4.03	3.21	22.42	$1.19x10^{-5}$	$9.05x10^{-5}$
SEZ6	-8.02	-1.01	22.15	$1.32x10^{-5}$	$9.92x10^{-5}$
ADAMTSL1	-2.50	3.76	22.09	$1.35x10^{-5}$	$1.01x10^{-4}$
STK3	1.62	3.30	20.49	$2.54x10^{-5}$	$1.77x10^{-4}$
GABRE	-2.37	4.52	18.85	$4.93x10^{-5}$	$3.15x10^{-5}$
LMNTD2	-2.74	3.33	17.12	$1.01x10^{-4}$	$5.78x10^{-4}$
KCNH6	-5.77	0.11	16.85	$1.13x10^{-4}$	$6.36x10^{-4}$
OBSCN	-2.33	4.90	16.84	$1.13x10^{-4}$	$6.38x10^{-4}$
SLC27A6	-5.11	-0.32	16.59	$1.26x10^{-4}$	$6.99x10^{-4}$
ITGA8	-2.49	3.84	16.51	$1.30x10^{-4}$	$7.19x10^{-4}$
CCDC180	-2.92	2.52	16.07	$1.57x10^{-4}$	$8.47x10^{-4}$
ATN1	-1.88	7.55	15.48	$2.02x10^{-4}$	$1.05x10^{-3}$
DCDC2B	-2.03	0.49	15.15	$2.32x10^{-4}$	$1.19x10^{-3}$

FER1L6	-5.05	4.76	12.51	7.42×10^{-4}	3.20×10^{-3}
GNMT	-2.97	-1.62	11.69	1.08×10^{-3}	4.38×10^{-3}
GATA2	-2.12	3.18	10.75	1.66×10^{-3}	6.32×10^{-3}
FTCD	-5.68	-1.07	8.92	3.95×10^{-3}	1.31×10^{-2}
LRRC34	1.55	-0.59	8.82	4.13×10^{-3}	1.36×10^{-2}
PEX6	-2.05	5.09	7.72	7.09×10^{-3}	2.11×10^{-2}
ABI3BP	-3.07	4.91	7.62	7.47×10^{-3}	2.20×10^{-2}
PRDM16	-2.53	1.35	7.54	7.76×10^{-3}	2.27×10^{-2}
ASIC4	-6.39	-3.20	7.42	8.22×10^{-3}	2.38×10^{-2}
ABCA3	-2.49	3.32	7.31	8.70×10^{-3}	2.49×10^{-2}
WDR17	-3.04	1.23	6.23	1.50×10^{-2}	3.90×10^{-2}
KIF5C	-2.94	3.17	6.11	1.60×10^{-2}	4.09×10^{-2}
ARHGAP10	-2.46	4.23	5.65	2.04×10^{-2}	4.98×10^{-2}

Supplementary Table 31. Genes with rare germline variants identified in patient TCGA-CK-4948 using 'All vs One' approach. This table lists genes identified through the 'All vs One' approach that harbour rare germline variants in patient TCGA-CK-4948. Genes shown meet the differential expression threshold of |log2 fold change| ≥1.5 and FDR <5%. Variants were retained on filtering criteria for rare population frequency. logFC: log2 fold change in gene expression; logCPM: log2-transformed counts per million across all samples; F: quasi-likelihood F-test statistic; p-value: nominal p-value (uncorrected); FDR: p-value adjusted using Benjamini-Hochberg correction.

	logFC	logCPM	F	p-value	FDR
CADPS	3.96	2.06	143.68	2.75×10^{-18}	7.85×10^{-16}
TBCD	1.60	5.66	68.52	7.90×10^{-12}	4.83×10^{-10}
ADRM1	1.96	5.99	63.31	2.99×10^{-11}	1.55×10^{-9}
PODNL1	2.65	1.65	58.11	1.19×10^{-10}	5.22×10^{-9}
TREX2	1.80	3.28	49.37	1.39×10^{-9}	4.54×10^{-8}
GBA2	-2.01	7.67	48.81	1.64×10^{-9}	5.26×10^{-8}
ABCA8	-7.10	5.05	47.28	2.59×10^{-9}	7.81×10^{-8}
RRS1	2.72	4.13	46.33	3.44×10^{-9}	1.00×10^{-7}
MYOT	-7.73	1.41	43.40	8.41×10^{-9}	2.18×10^{-7}
VWA2	4.46	0.96	40.58	2.03×10^{-8}	4.63×10^{-7}
HAUS7	1.61	3.52	38.31	4.21×10^{-8}	8.64×10^{-7}
VARS1	1.68	5.97	35.66	1.01×10^{-7}	1.83×10^{-6}
PLXND1	1.64	5.55	26.39	2.63×10^{-6}	3.11×10^{-5}
CPEB3	-2.24	3.41	22.72	1.06×10^{-5}	1.04×10^{-4}
POLR1B	1.53	4.22	20.99	2.08×10^{-5}	1.87×10^{-4}
MMRN1	-6.01	3.93	17.44	8.80×10^{-5}	6.34×10^{-4}
ASXL3	-4.14	2.32	16.27	1.44×10^{-4}	9.56×10^{-4}
CHODL	-4.36	0.19	15.60	1.91×10^{-4}	1.22×10^{-3}
ADAM33	-6.54	6.11	15.13	2.34×10^{-4}	1.44×10^{-3}
VPS13A	-1.91	6.60	14.73	2.79×10^{-4}	1.66×10^{-3}
PITPNM3	-1.64	5.37	14.55	3.01×10^{-4}	1.78×10^{-3}
ZNF91	-1.58	5.36	14.53	3.04×10^{-4}	1.79×10^{-3}
SULT1A2	-4.37	3.98	13.97	3.87×10^{-4}	2.20×10^{-3}
ROBO2	2.47	0.63	13.46	4.86×10^{-4}	2.67×10^{-3}
RAPGEF3	-2.00	4.32	12.20	8.52×10^{-4}	4.23×10^{-3}
LCAT	-2.19	3.12	11.25	1.31×10^{-3}	6.08×10^{-3}
HTRA3	1.94	3.90	11.14	1.39×10^{-3}	6.33×10^{-3}
TTBK2	-1.68	4.01	10.92	1.53×10^{-3}	6.87×10^{-3}
FAM13A	-1.58	5.87	10.72	1.68×10^{-3}	7.40×10^{-3}
CDH23	-2.33	2.55	10.43	1.92×10^{-3}	8.23×10^{-3}
CPA3	-2.92	4.20	8.82	4.14×10^{-3}	1.55×10^{-2}
NPY	-8.00	-0.93	7.93	6.38×10^{-3}	2.20×10^{-2}
SLC26A9	2.86	-3.85	7.49	7.96×10^{-3}	2.62×10^{-2}
ZFR2	-4.03	-2.10	7.26	8.92×10^{-3}	2.88×10^{-2}
ALK	-4.50	-0.97	7.25	8.97×10^{-3}	2.90×10^{-2}
AQP8	-6.88	8.54	6.84	1.10×10^{-2}	3.41×10^{-2}

Supplementary Table 32. Genes with rare germline variants identified in patient TCGA-CK-6748 using 'All vs One' approach. This table lists genes identified through the 'All vs One' approach that harbour rare germline variants in patient TCGA-CK-6748. Genes shown meet the differential expression threshold of |log2 fold change| ≥1.5 and FDR <5%. Variants were retained on filtering criteria for rare population frequency. logFC: log2 fold change in gene expression; logCPM: log2-transformed counts per million across all samples; F: quasi-likelihood F-test statistic; p-value: nominal p-value (uncorrected); FDR: p-value adjusted using Benjamini-Hochberg correction.

	logFC	logCPM	F	p-value	FDR
STAU1	2.33	6.64	267.93	$4.99x10^{-25}$	$5.06x10^{-22}$
ITGA11	4.78	2.73	212.68	$2.08x10^{-22}$	$1.30x10^{-19}$
BCL2L1	2.37	6.22	122.81	$9.24x10^{-17}$	$1.86x10^{-14}$
PLA2G4B	-3.41	4.31	80.74	$4.29x10^{-13}$	$3.39x10^{-11}$
ATIC	1.78	5.80	67.24	$1.10x10^{-11}$	$6.06x10^{-10}$
CDYL2	2.49	2.63	62.24	$3.96x10^{-11}$	$1.86x10^{-9}$
ROBO2	4.24	0.75	58.68	$1.02x10^{-10}$	$4.34x10^{-9}$
DKC1	2.18	5.69	48.12	$2.02x10^{-9}$	$6.03x10^{-8}$
DNAH1	-3.66	5.06	42.60	$1.08x10^{-8}$	$2.60x10^{-7}$
UGGT2	1.68	3.85	41.82	$1.38x10^{-8}$	$3.23x10^{-7}$
NRBP2	-3.52	6.23	40.63	$2.01x10^{-8}$	$4.45x10^{-7}$
CMSS1	2.03	2.81	40.26	$2.26x10^{-8}$	$4.95x10^{-7}$
TOP3B	-2.04	4.57	36.98	$6.54x10^{-8}$	$1.29x10^{-6}$
IRS2	2.63	5.32	36.71	$7.15x10^{-8}$	$1.40x10^{-6}$
PAN2	-1.91	5.95	32.40	$3.07x10^{-7}$	$4.99x10^{-6}$
CDKL1	-1.91	3.06	25.96	$3.09x10^{-6}$	$3.67x10^{-5}$
PLEKHH1	-2.21	6.44	25.56	$3.58x10^{-6}$	$4.17x10^{-5}$
PTPRCAP	-3.54	4.00	21.48	$1.72x10^{-5}$	$1.59x10^{-4}$
TPH1	-7.19	4.54	21.24	$1.88x10^{-5}$	$1.71x10^{-4}$
TNS2	-2.28	6.76	19.22	$4.24x10^{-5}$	$3.41x10^{-4}$
IL16	-2.44	4.99	18.17	$6.51x10^{-5}$	$4.91x10^{-4}$
LCN12	-3.34	1.55	18.15	$6.58x10^{-5}$	$4.95x10^{-4}$
IFFO1	-2.17	4.12	17.47	$8.72x10^{-5}$	$6.26x10^{-4}$
SEC24D	1.56	5.73	17.06	$1.03x10^{-4}$	$7.23x10^{-4}$
CSMD2	2.10	-0.37	15.24	$2.24x10^{-4}$	$1.37x10^{-3}$
CEP290	-1.52	4.21	15.12	$2.36x10^{-4}$	$1.43x10^{-3}$
CYP3A7	2.76	-0.83	14.12	$3.64x10^{-4}$	$2.06x10^{-3}$
EPDR1	1.73	3.81	13.36	$5.08x10^{-4}$	$2.71x10^{-3}$
LRRC69	-2.20	-0.83	12.39	$7.84x10^{-4}$	$3.92x10^{-3}$
STAG3	-2.08	2.59	11.36	$1.25x10^{-3}$	$5.75x10^{-3}$
USH2A	-3.37	-1.73	10.81	$1.62x10^{-3}$	$7.10x10^{-3}$
AEBP1	1.75	7.07	10.80	$1.62x10^{-3}$	$7.12x10^{-3}$
SH2D7	-2.78	2.47	8.85	$4.07x10^{-3}$	$1.51x10^{-2}$
AVPR1B	-3.34	-1.58	7.57	$7.64x10^{-3}$	$2.52x10^{-2}$
DISP2	-3.63	4.34	7.47	$8.04x10^{-3}$	$2.63x10^{-2}$
ACADL	-3.45	-1.68	6.64	$1.22x10^{-2}$	$3.67x10^{-2}$

Supplementary Table 33. Genes with rare germline variants identified in patient TCGA-CM-5860 using 'All vs One' approach. This table lists genes identified through the 'All vs One' approach that harbour rare germline variants in patient TCGA-CM-5860. Genes shown meet the differential expression threshold of |log2 fold change| ≥1.5 and FDR <5%. Variants were retained on filtering criteria for rare population frequency. logFC: log2 fold change in gene expression; logCPM: log2-transformed counts per million across all samples; F: quasi-likelihood F-test statistic; p-value: nominal p-value (uncorrected); FDR: p-value adjusted using Benjamini-Hochberg correction.

	logFC	logCPM	F	p-value	FDR
COL11A1	7.29	1.02	318.18	$4.66x10^{-27}$	$1.24x10^{-23}$
GLA	2.07	3.65	109.24	$1.12x10^{-15}$	$1.62x10^{-13}$
MTMR6	2.09	4.88	91.33	$4.15x10^{-14}$	$4.03x10^{-12}$
RAD21	1.69	7.18	83.23	$2.44x10^{-13}$	$1.92x10^{-11}$
ZNF92	2.13	3.02	71.70	$3.62x10^{-12}$	$2.01x10^{-10}$
CSAD	-2.98	4.71	55.17	$2.67x10^{-10}$	$8.67x10^{-9}$
CHSY3	3.48	-0.67	52.59	$5.52x10^{-10}$	$1.65x10^{-8}$
AIFM3	-4.27	6.44	40.52	$2.08x10^{-8}$	$4.07x10^{-7}$
VPS13D	-1.61	4.49	39.08	$3.29x10^{-8}$	$6.07x10^{-7}$
TTN	-3.98	4.37	32.01	$3.50x10^{-7}$	$4.63x10^{-6}$
MASTL	1.56	3.29	29.47	$8.58x10^{-7}$	$1.02x10^{-5}$
EFCAB11	1.64	1.99	28.46	$1.23x10^{-6}$	$1.40x10^{-5}$
PTPN18	-1.73	7.00	28.41	$1.26x10^{-6}$	$1.42x10^{-5}$
DPP4	2.20	4.84	26.89	$2.19x10^{-6}$	$2.32x10^{-5}$
PKHD1L1	-6.07	2.45	23.12	$9.04x10^{-6}$	$7.92x10^{-5}$
ANKK1	-8.75	-0.95	20.76	$2.28x10^{-5}$	$1.77x10^{-4}$
NIFK	1.53	4.63	20.52	$2.51x10^{-5}$	$1.93x10^{-4}$
AHNAK	-2.94	10.54	20.02	$3.07x10^{-5}$	$2.28x10^{-4}$
PITPNM3	-2.01	5.36	20.00	$3.09x10^{-5}$	$2.30x10^{-4}$
CFAP70	-2.46	1.88	19.72	$3.47x10^{-5}$	$2.54x10^{-4}$
CACNA1F	-5.45	1.65	16.62	$1.24x10^{-4}$	$7.50x10^{-4}$
ZNF692	-2.07	4.95	16.12	$1.54x10^{-4}$	$9.00x10^{-4}$
NEIL3	2.41	0.63	15.99	$1.62x10^{-4}$	$9.42x10^{-4}$
FAT2	-3.21	0.68	15.76	$1.79x10^{-4}$	$1.02x10^{-3}$
SGSM1	-3.71	2.67	15.75	$1.80x10^{-4}$	$1.03x10^{-3}$
BCO2	-2.88	1.33	14.98	$2.51x10^{-4}$	$1.36x10^{-3}$
PLEC	-1.61	8.98	14.93	$2.55x10^{-4}$	$1.38x10^{-3}$
PCED1A	-1.66	5.25	14.67	$2.86x10^{-4}$	$1.51x10^{-3}$
PCDH17	2.10	1.46	14.58	$2.98x10^{-4}$	$1.56x10^{-3}$
UTY	-2.66	3.67	13.60	$4.58x10^{-4}$	$2.25x10^{-3}$
SNAP91	-8.98	-0.24	13.50	$4.77x10^{-4}$	$2.33x10^{-3}$

COL5A1	2.00	6.61	11.82	1.01×10^{-3}	4.37×10^{-3}
ASTN1	-4.85	-0.40	11.77	1.04×10^{-3}	4.45×10^{-3}
FIGN	-2.88	2.00	11.56	1.14×10^{-3}	4.82×10^{-3}
RAET1E	2.26	-2.22	10.65	1.74×10^{-3}	6.82×10^{-3}
SERPINA4	3.38	-3.87	10.50	1.90×10^{-3}	7.35×10^{-3}
TMEM171	-2.81	5.50	10.27	2.08×10^{-3}	7.92×10^{-3}
CD1A	3.38	-2.60	10.05	2.31×10^{-3}	8.62×10^{-3}
HMCN2	-4.84	5.47	9.97	2.39×10^{-3}	8.88×10^{-3}
OPLAH	-1.82	5.75	9.72	2.69×10^{-3}	9.78×10^{-3}
EFHB	-7.73	-1.36	9.24	3.39×10^{-3}	1.19×10^{-2}
PPP1R12C	-1.60	6.90	7.59	7.56×10^{-3}	2.32×10^{-2}
TMEM52B	2.51	-2.91	7.44	8.15×10^{-3}	2.46×10^{-2}
CDHR4	-6.82	-2.54	7.02	1.00×10^{-2}	2.92×10^{-2}
KLHL33	-3.58	-1.88	6.94	1.05×10^{-2}	3.02×10^{-2}
SVEP1	-2.21	4.75	6.34	1.42×10^{-2}	3.87×10^{-2}
PTGES3L-AARSD1	-2.34	0.70	6.01	1.69×10^{-2}	4.44×10^{-2}
SLC6A19	-9.24	5.11	6.00	1.69×10^{-2}	4.46×10^{-2}

Supplementary Table 34. Genes with rare germline variants identified in patient TCGA-CM-6162 using 'All vs One' approach. This table lists genes identified through the 'All vs One' approach that harbour rare germline variants in patient TCGA-CM-6162. Genes shown meet the differential expression threshold of |log2 fold change| ≥1.5 and FDR <5%. Variants were retained on filtering criteria for rare population frequency. logFC: log2 fold change in gene expression; logCPM: log2-transformed counts per million across all samples; F: quasi-likelihood F-test statistic; p-value: nominal p-value (uncorrected); FDR: p-value adjusted using Benjamini-Hochberg correction.

	logFC	logCPM	F	p-value	FDR
GABRP	5.84	-0.60	97.79	$1.08x10^{-14}$	$3.23x10^{-12}$
SPARC	3.35	8.03	96.60	$1.38x10^{-14}$	$3.91x10^{-12}$
TGFBI	3.19	6.26	65.96	$1.52x10^{-11}$	$2.08x10^{-9}$
TUBGCP6	-2.15	6.25	61.00	$5.50x10^{-11}$	$6.09x10^{-9}$
PRRT2	-5.49	4.56	53.86	$3.86x10^{-10}$	$3.17x10^{-8}$
EMILIN2	1.82	3.94	43.80	$7.44x10^{-9}$	$4.02x10^{-7}$
FAM162A	-2.42	6.96	35.88	$9.40x10^{-8}$	$3.15x10^{-6}$
SHROOM4	2.08	2.93	35.55	$1.13x10^{-7}$	$3.64x10^{-6}$
MROH7	-4.68	4.34	32.82	$2.65x10^{-7}$	$7.43x10^{-6}$
ADCY6	-1.95	7.65	32.52	$2.95x10^{-7}$	$8.10x10^{-6}$
SLC7A11	3.40	2.53	25.48	$3.70x10^{-6}$	$6.43x10^{-5}$
MBOAT1	-1.63	6.00	21.22	$1.90x10^{-5}$	$2.48x10^{-4}$
ZNF345	-2.47	2.65	20.91	$2.15x10^{-5}$	$2.75x10^{-4}$
ZNF536	-3.24	1.54	19.85	$3.28x10^{-5}$	$3.85x10^{-4}$
VWA5B2	-4.49	1.61	19.54	$3.72x10^{-5}$	$4.26x10^{-4}$
ATG4D	-2.08	5.68	18.04	$6.87x10^{-5}$	$7.06x10^{-4}$
HOOK2	-1.82	5.91	17.90	$7.27x10^{-5}$	$7.41x10^{-4}$
COL14A1	2.38	5.54	17.53	$8.48x10^{-5}$	$8.33x10^{-4}$
IGSF9	-4.16	6.09	17.48	$8.65x10^{-5}$	$8.44x10^{-4}$
MYO15A	-2.70	0.68	17.03	$1.05x10^{-4}$	$9.83x10^{-4}$
RIMBP2	-3.00	2.20	16.26	$1.45x10^{-4}$	$1.29x10^{-3}$
CHP2	-4.18	8.06	15.90	$1.69x10^{-4}$	$1.46x10^{-3}$
NGEF	-2.07	5.22	15.49	$2.01x10^{-4}$	$1.67x10^{-3}$
SPRED1	1.67	5.35	15.40	$2.08x10^{-4}$	$1.72x10^{-3}$
KLHL31	-3.34	0.37	14.85	$2.65x10^{-4}$	$2.08x10^{-3}$
KIF18A	2.00	1.58	14.02	$3.80x10^{-4}$	$2.80x10^{-3}$
PGPEP1	-1.58	6.41	13.90	$4.01x10^{-4}$	$2.92x10^{-3}$
ULBP3	2.38	-1.72	11.67	$1.09x10^{-3}$	$6.50x10^{-3}$
PLEKHG6	-2.29	6.43	11.20	$1.35x10^{-3}$	$7.70x10^{-3}$
TTN	-1.88	4.37	10.10	$2.25x10^{-3}$	$1.16x10^{-2}$
COL4A4	1.72	2.12	9.53	$2.95x10^{-3}$	$1.42x10^{-2}$
NEURL1	-2.12	5.56	9.02	$3.77x10^{-3}$	$1.72x10^{-2}$
LRRC4B	-1.68	1.34	7.86	$6.62x10^{-3}$	$2.65x10^{-2}$
GDNF	-3.37	2.36	7.80	$6.82x10^{-3}$	$2.72x10^{-2}$
NRXN1	-3.16	2.39	7.74	$7.01x10^{-3}$	$2.78x10^{-2}$
CGN	-1.59	7.85	7.74	$7.01x10^{-3}$	$2.78x10^{-2}$
NEXMIF	-2.66	1.44	7.45	$8.10x10^{-3}$	$3.10x10^{-2}$
SLC6A13	-5.97	-3.48	7.25	$8.94x10^{-3}$	$3.34x10^{-2}$
TYMP	1.62	5.06	6.93	$1.05x10^{-3}$	$3.80x10^{-2}$
STAB2	-2.83	1.27	6.40	$1.38x10^{-2}$	$4.67x10^{-2}$

Supplementary Table 35. Genes with rare germline variants identified in patient TCGA-EI-7004 using 'All vs One' approach. This table lists genes identified through the 'All vs One' approach that harbour rare germline variants in patient TCGA-EI-7004. Genes shown meet the differential expression threshold of |log2 fold change| ≥1.5 and FDR <5%. Variants were retained on filtering criteria for rare population frequency. logFC: log2 fold change in gene expression; logCPM: log2-transformed counts per million across all samples; F: quasi-likelihood F-test statistic; p-value: nominal p-value (uncorrected); FDR: p-value adjusted using Benjamini-Hochberg correction.

	**logFC**	**logCPM**	**F**	**p-value**	**FDR**
P4HA3	4.22	0.82	155.09	4.74×10^{-19}	3.40×10^{-16}
COL18A1	3.64	6.79	121.29	1.21×10^{-16}	5.28×10^{-14}
KIAA1549	2.42	2.63	70.73	4.60×10^{-12}	6.01×10^{-10}
ADAMTS2	2.97	4.33	43.12	9.18×10^{-9}	4.38×10^{-7}
PAN2	-1.97	5.95	34.17	1.67×10^{-7}	5.14×10^{-6}
MTHFD1L	2.79	2.93	28.60	1.17×10^{-7}	2.59×10^{-5}
SMPD3	-2.90	6.47	27.37	1.83×10^{-6}	3.70×10^{-5}
CA2	-5.10	9.77	24.90	4.59×10^{-6}	7.88×10^{-5}
RELN	2.83	1.92	24.00	6.46×10^{-6}	1.04×10^{-4}
NPHP3	-2.06	5.78	23.96	6.57×10^{-6}	1.06×10^{-4}
PCGF3	-1.51	6.56	20.15	2.92×10^{-5}	3.60×10^{-4}
CES2	-2.97	9.18	18.83	4.97×10^{-5}	5.55×10^{-4}
CGREF1	2.40	2.56	18.59	5.49×10^{-5}	6.01×10^{-4}
IL34	2.41	1.53	17.16	9.90×10^{-5}	9.76×10^{-4}
SCARA5	-3.22	5.96	15.98	1.63×10^{-4}	1.47×10^{-3}
HSD17B13	-4.02	0.16	15.51	1.99×10^{-4}	1.73×10^{-3}
TTN	-2.27	4.37	13.75	4.28×10^{-4}	3.18×10^{-3}
RRP12	1.68	4.43	13.42	4.94×10^{-4}	3.59×10^{-3}
SEC16B	-2.04	4.28	13.28	5.26×10^{-4}	3.75×10^{-3}
CRISPLD1	3.18	0.38	12.64	7.00×10^{-4}	4.73×10^{-3}
HEPHL1	-7.44	-1.70	11.90	9.80×10^{-4}	6.22×10^{-3}
PTGDR2	-4.22	4.15	11.24	1.33×10^{-3}	7.88×10^{-3}
MMP10	4.39	-0.58	11.24	1.33×10^{-3}	7.88×10^{-3}
VWA5A	-1.78	6.36	10.97	1.50×10^{-3}	8.67×10^{-3}
RIPOR3	-1.52	3.91	10.61	1.77×10^{-3}	9.89×10^{-3}
UGT1A7	-5.21	-1.47	10.39	1.97×10^{-3}	1.08×10^{-2}
STPG3	-4.27	0.05	9.62	2.82×10^{-3}	1.43×10^{-2}
GNG13	-5.57	-0.02	9.55	2.91×10^{-3}	1.47×10^{-2}
UGT1A4	-3.03	6.15	9.53	2.94×10^{-3}	1.48×10^{-2}
UGT1A6	-3.01	6.61	9.31	3.27×10^{-3}	1.61×10^{-2}
UGT1A10	-2.92	6.95	9.23	3.40×10^{-3}	1.65×10^{-2}
ADAMTS17	-1.84	2.10	8.75	4.29×10^{-3}	1.99×10^{-2}
IQCN	-2.54	2.13	8.33	5.26×10^{-3}	2.32×10^{-2}
FER1L6	2.06	4.80	8.17	5.69×10^{-3}	2.47×10^{-2}
GP2	-3.82	2.74	7.81	6.77×10^{-3}	2.83×10^{-2}
NOXO1	-1.92	3.87	6.58	1.26×10^{-2}	4.49×10^{-2}

Supplementary Table 36. Genes with rare germline variants identified in patient 103 using 'All vs One' approach. This table lists genes identified through the 'All vs One' approach that harbour rare germline variants in patient 103. Genes shown meet the differential expression threshold of |log2 fold change| ≥1.5 and FDR <5%. Variants were retained on filtering criteria for rare population frequency. logFC: log2 fold change in gene expression; logCPM: log2-transformed counts per million across all samples; F: quasi-likelihood F-test statistic; p-value: nominal p-value (uncorrected); FDR: p-value adjusted using Benjamini-Hochberg correction.

	logFC	logCPM	F	p-value	FDR
KRT80	5.94	-0.28	101.22	5.37×10^{-15}	3.00×10^{-12}
CMSS1	2.88	2.90	90.09	5.40×10^{-14}	1.98×10^{-11}
COL8A1	5.19	2.90	50.74	9.39×10^{-10}	8.83×10^{-8}
PLCB4	2.43	5.78	47.88	2.17×10^{-9}	1.85×10^{-7}
NCOA7	2.83	5.24	30.84	5.28×10^{-7}	2.01×10^{-5}
ENDOD1	-3.06	6.92	29.99	7.13×10^{-7}	2.58×10^{-5}
DNAH2	2.38	-0.03	28.15	1.38×10^{-6}	4.52×10^{-5}
OSM	4.38	-0.84	22.86	1.00×10^{-5}	2.32×10^{-4}
SIDT2	-1.61	6.27	21.53	1.68×10^{-5}	3.54×10^{-4}
CLCA1	-6.82	10.39	21.50	1.70×10^{-5}	3.57×10^{-4}
CARD11	2.56	2.94	20.30	2.75×10^{-5}	5.25×10^{-4}
ASIC3	-3.89	1.62	18.17	6.52×10^{-5}	1.04×10^{-3}
ZNF469	2.11	0.95	17.21	9.70×10^{-5}	1.43×10^{-3}
ZNF491	-2.51	0.97	16.19	1.49×10^{-4}	1.99×10^{-3}
AKAP9	-1.74	7.48	15.83	1.74×10^{-4}	2.23×10^{-3}
NPY4R	-4.96	3.22	15.66	1.87×10^{-4}	2.36×10^{-3}
BRCA2	2.34	2.11	15.66	1.87×10^{-4}	2.36×10^{-3}
RSPO4	3.36	-1.70	15.30	2.18×10^{-4}	2.66×10^{-3}
LRRN2	-4.53	4.71	14.45	3.14×10^{-4}	3.52×10^{-3}
PLEKHA4	2.22	3.64	13.94	3.94×10^{-4}	4.20×10^{-3}
SEC14L6	-5.21	-1.37	12.75	6.68×10^{-4}	6.32×10^{-3}
PBLD	-3.18	6.06	12.65	6.98×10^{-4}	6.54×10^{-3}
TTC39A	-2.15	5.83	11.47	1.19×10^{-3}	9.77×10^{-3}
LRRC75B	-1.52	3.48	10.23	2.12×10^{-3}	1.51×10^{-2}
IGSF9	-2.86	6.09	9.93	2.44×10^{-3}	1.69×10^{-2}
CD1C	-1.53	3.16	9.25	3.36×10^{-3}	2.15×10^{-2}
DNAI1	-5.33	-2.85	7.11	9.61×10^{-3}	4.60×10^{-2}

Supplementary Table 37. Genes with rare germline variants identified in patient 109 using 'All vs One' approach. This table lists genes identified through the 'All vs One' approach that harbour rare germline variants in patient 109. Genes shown meet the differential expression threshold of |log2 fold change| ≥1.5 and FDR <5%. Variants were retained on filtering criteria for rare population frequency. logFC: log2 fold change in gene expression; logCPM: log2-transformed counts per million across all samples; F: quasi-likelihood F-test statistic; p-value: nominal p-value (uncorrected); FDR: p-value adjusted using Benjamini-Hochberg correction.

	logFC	logCPM	F	p-value	FDR
SLCO1B3	7.90	-0.71	56.43	$1.89x10^{-10}$	$1.80x10^{-8}$
HCAR3	5.42	-1.71	43.45	$8.30x10^{-9}$	$5.09x10^{-7}$
PLCE1	-2.42	6.83	35.22	$1.17x10^{-7}$	$5.23x10^{-6}$
PACC1	1.91	1.61	31.64	$4.00x10^{-7}$	$1.50x10^{-5}$
GBA2	-1.53	7.67	30.51	$5.94x10^{-7}$	$2.12x10^{-5}$
NECTIN3	-2.28	5.08	30.01	$7.08x10^{-7}$	$2.47x10^{-5}$
TRIM46	2.40	0.16	27.98	$1.47x10^{-6}$	$4.59x10^{-5}$
PTGDR	-4.55	3.99	23.52	$7.76x10^{-6}$	$1.91x10^{-4}$
CALHM3	4.88	-4.38	23.29	$9.09x10^{-6}$	$2.18x10^{-4}$
VWCE	2.88	-0.51	22.23	$1.28x10^{-5}$	$2.88x10^{-4}$
ANO7	-3.75	6.18	19.91	$3.20x10^{-5}$	$6.07x10^{-4}$
MYCBPAP	-5.41	0.20	17.53	$8.49x10^{-5}$	$1.33x10^{-3}$
RIMKLA	-3.21	4.54	17.34	$9.17x10^{-5}$	$1.42x10^{-3}$
CNGB3	3.54	-3.81	16.36	$1.39x10^{-4}$	$2.00x10^{-3}$
GAD1	3.23	-2.64	15.85	$1.72x10^{-4}$	$2.36x10^{-3}$
BACE2	1.74	5.75	15.77	$1.78x10^{-4}$	$2.43x10^{-3}$
KIF26A	-2.93	3.49	14.71	$2.82x10^{-4}$	$3.52x10^{-3}$
DOCK5	-1.60	6.25	14.51	$3.06x10^{-4}$	$3.75x10^{-3}$
COL12A1	3.12	5.94	14.39	$3.24x10^{-4}$	$3.92x10^{-3}$
OTOP2	-8.26	5.29	14.28	$3.39x10^{-4}$	$4.08x10^{-3}$
IGFBP1	4.16	-3.11	14.18	$3.54x10^{-4}$	$4.20x10^{-3}$
LYAR	1.51	3.41	12.78	$6.59x10^{-4}$	$6.77x10^{-3}$
ABCA10	-2.41	3.03	12.42	$7.75x10^{-4}$	$7.69x10^{-3}$
DOP1B	-1.50	6.24	12.05	$9.15x10^{-4}$	$8.74x10^{-3}$
AGFG2	-1.82	6.11	11.33	$1.27x10^{-3}$	$1.12x10^{-2}$
PCLO	-2.52	3.90	10.42	$1.94x10^{-3}$	$1.54x10^{-2}$
ANLN	2.14	3.51	10.26	$2.09x10^{-3}$	$1.63x10^{-2}$
PROB1	-2.10	2.72	9.82	$2.56x10^{-3}$	$1.89x10^{-2}$
KDM5D	-2.03	5.55	9.79	$2.61x10^{-3}$	$1.92x10^{-2}$
CCDC178	-5.60	-2.42	9.76	$2.64x10^{-3}$	$1.93x10^{-2}$
HOXA1	-1.74	1.08	9.68	$2.74x10^{-3}$	$1.98x10^{-2}$
DLL3	2.22	-2.66	9.58	$2.88x10^{-3}$	$2.06x10^{-2}$
CDH23	-2.22	2.55	9.42	$3.10x10^{-3}$	$2.18x10^{-2}$
SIGLEC1	-2.09	4.91	9.01	$3.78x10^{-3}$	$2.51x10^{-2}$
APCDD1L	-7.85	-0.50	8.62	$4.55x10^{-3}$	$2.90x10^{-2}$
AHNAK	-1.69	10.54	8.00	$6.19x10^{-3}$	$3.63x10^{-2}$
SLC14A1	-2.03	1.34	7.96	$6.29x10^{-3}$	$3.67x10^{-2}$
XKR6	-1.51	0.61	7.46	$8.09x10^{-3}$	$4.43x10^{-2}$

Supplementary Table 38. Genes with rare germline variants identified in patient 402 using 'All vs One' approach. This table lists genes identified through the 'All vs One' approach that harbour rare germline variants in patient 402. Genes shown meet the differential expression threshold of |log2 fold change| ≥1.5 and FDR <5%. Variants were retained on filtering criteria for rare population frequency. logFC: log2 fold change in gene expression; logCPM: log2-transformed counts per million across all samples; F: quasi-likelihood F-test statistic; p-value: nominal p-value (uncorrected); FDR: p-value adjusted using Benjamini-Hochberg correction.

	logFC	logCPM	F	p-value	FDR
SBF1	-1.61	6.87	48.34	1.89×10^{-9}	7.14×10^{-8}
POLR1C	2.23	4.11	45.82	4.02×10^{-9}	1.37×10^{-7}
XKRX	3.88	0.65	40.94	1.82×10^{-8}	4.88×10^{-7}
XPOT	1.90	5.49	39.45	2.93×10^{-8}	7.35×10^{-7}
DDN	5.42	-1.81	37.55	5.42×10^{-8}	1.24×10^{-6}
ZNF749	2.46	1.72	32.63	2.83×10^{-7}	5.16×10^{-6}
MRPS12	1.94	4.74	31.36	4.40×10^{-7}	7.57×10^{-6}
AHNAK	-3.92	10.53	29.04	1.00×10^{-6}	1.55×10^{-5}
TPCN1	-1.69	6.82	27.04	2.07×10^{-6}	2.87×10^{-5}
KIF13A	-2.15	6.00	25.73	3.37×10^{-6}	4.38×10^{-5}
P4HA2	-2.44	5.38	23.29	8.48×10^{-6}	9.60×10^{-5}
SYNE2	-2.33	7.58	23.24	8.66×10^{-6}	9.79×10^{-5}
CCN5	-7.26	3.22	21.16	1.95×10^{-5}	1.95×10^{-5}
TRPV6	-6.28	-1.03	18.48	5.73×10^{-5}	4.90×10^{-4}
DNAJA1	2.42	6.91	17.34	9.18×10^{-5}	7.28×10^{-4}
SCN11A	-5.62	0.25	16.13	1.53×10^{-4}	1.13×10^{-3}
CBX2	2.69	0.27	15.51	1.99×10^{-4}	1.42×10^{-3}
WNK2	-1.60	7.40	15.22	2.25×10^{-4}	1.57×10^{-3}
ABI3BP	-4.90	4.91	14.32	3.32×10^{-4}	2.16×10^{-3}
ZNF256	-1.75	1.77	14.18	3.55×10^{-4}	2.28×10^{-3}
TRPM5	-4.09	2.96	12.20	8.53×10^{-4}	4.71×10^{-3}
ANK3	-2.41	6.03	10.68	1.71×10^{-3}	8.28×10^{-3}
NBEAL1	-2.06	5.10	10.53	1.84×10^{-3}	8.80×10^{-3}
SLC9A9	-1.89	3.15	10.39	1.96×10^{-3}	9.24×10^{-3}
PDE2A	-2.78	4.49	10.05	2.30×10^{-3}	1.05×10^{-2}
CHAC1	2.30	0.86	8.93	3.92×10^{-3}	1.60×10^{-2}
WDR64	-6.04	-2.97	8.67	4.44×10^{-3}	1.76×10^{-2}
ACADL	-4.57	-1.69	8.29	5.34×10^{-3}	2.05×10^{-2}
PRDM16	-2.60	1.34	7.24	9.02×10^{-3}	3.12×10^{-2}
FRMD3	-1.98	4.41	7.19	9.23×10^{-3}	3.18×10^{-2}
RECQL4	1.51	3.95	6.97	1.03×10^{-2}	3.47×10^{-2}
CCDC88A	-1.59	3.99	6.65	1.22×10^{-2}	3.94×10^{-2}
CCDC110	-1.79	0.39	6.63	1.22×10^{-2}	3.96×10^{-2}
GRIN3A	-2.90	0.90	6.56	1.27×10^{-2}	4.08×10^{-2}
CDHR2	-2.92	6.69	6.53	1.29×10^{-2}	4.12×10^{-2}
ZNF98	-6.06	-3.65	6.49	1.31×10^{-2}	4.18×10^{-2}
SV2B	-2.92	0.84	6.11	1.60×10^{-2}	4.88×10^{-2}

Supplementary Table 39. Genes with rare germline variants identified in patient 501 using 'All vs One' approach. This table lists genes identified through the 'All vs One' approach that harbour rare germline variants in patient 501. Genes shown meet the differential expression threshold of |log2 fold change| ≥1.5 and FDR <5%. Variants were retained on filtering criteria for rare population frequency. logFC: log2 fold change in gene expression; logCPM: log2-transformed counts per million across all samples; F: quasi-likelihood F-test statistic; p-value: nominal p-value (uncorrected); FDR: p-value adjusted using Benjamini-Hochberg correction.

	logFC	logCPM	F	p-value	FDR
SIPA1L2	-2.75	6.38	87.70	$9.06x10^{-14}$	$1.32x10^{-11}$
METTL26	2.34	4.51	72.81	$2.76x10^{-12}$	$2.74x10^{-10}$
RPUSD4	1.68	4.36	57.37	$1.46x10^{-10}$	$8.30x10^{-9}$
UBA2	1.78	5.82	56.90	$1.66x10^{-10}$	$9.25x10^{-9}$
POLA1	2.24	2.84	55.09	$2.73x10^{-10}$	$1.42x10^{-8}$
COMMD6	1.82	5.48	41.56	$1.50x10^{-8}$	$4.33x10^{-7}$
SETMAR	1.51	3.46	40.95	$1.81x10^{-8}$	$5.13x10^{-7}$
GPT	-5.59	6.51	39.94	$2.50x10^{-8}$	$6.69x10^{-7}$
TEP1	-2.26	6.68	37.44	$5.62x10^{-8}$	$1.35x10^{-6}$
TMEM208	1.74	4.53	36.70	$7.16x10^{-8}$	$1.66x10^{-6}$
B3GNTL1	1.57	2.69	35.10	$1.22x10^{-7}$	$2.59x10^{-6}$
CSTF2	1.81	4.23	34.00	$1.77x10^{-7}$	$3.56x10^{-6}$
PADI2	-4.75	8.53	27.52	$1.74x10^{-6}$	$2.52x10^{-5}$
DGKA	-2.20	6.06	26.89	$2.19x10^{-6}$	$3.08x10^{-5}$
SPTB	-2.37	2.99	26.65	$2.39x10^{-6}$	$3.33x10^{-5}$
SSTR1	-2.62	3.66	25.28	$3.99x10^{-6}$	$5.10x10^{-5}$
HAGHL	1.57	3.10	23.63	$7.43x10^{-6}$	$8.68x10^{-5}$
NOP14	1.59	5.24	23.36	$8.27x10^{-6}$	$9.51x10^{-5}$
TNXB	-6.26	5.49	21.28	$1.86x10^{-5}$	$1.90x10^{-4}$
PTPRF	-1.88	9.01	20.98	$2.09x10^{-5}$	$2.10x10^{-4}$
MYCBPAP	-7.80	0.19	20.43	$2.60x10^{-5}$	$2.50x10^{-4}$
MYH3	-2.53	2.38	19.63	$3.59x10^{-5}$	$3.28x10^{-4}$
ADGRV1	-2.57	1.67	18.40	$5.92x10^{-5}$	$4.98x10^{-4}$
DAAM2	-2.51	5.48	17.17	$9.87x10^{-5}$	$7.68x10^{-4}$
SEMA3D	-4.18	1.57	16.26	$1.45x10^{-4}$	$1.06x10^{-3}$
CAPN9	-3.97	4.97	15.32	$2.16x10^{-4}$	$1.47x10^{-3}$
MICAL3	-1.53	6.84	15.21	$2.26x10^{-4}$	$1.53x10^{-3}$
LRP1	-1.91	8.27	14.99	$2.49x10^{-4}$	$1.65x10^{-3}$
SHROOM3	-1.76	7.50	14.19	$3.52x10^{-4}$	$2.20x10^{-3}$
CD1D	-1.89	3.16	12.54	$7.33x10^{-4}$	$4.01x10^{-3}$
ROS1	-9.84	0.77	12.52	$7.39x19^{-4}$	$4.03x10^{-3}$
RANBP17	1.57	1.76	12.21	$8.51x10^{-4}$	$4.55x10^{-3}$

FBLN2	-3.25	5.30	10.43	$1.93x10^{-3}$	$8.87x10^{-3}$
SLC36A1	-2.67	6.29	9.71	$2.71x10^{-3}$	$1.16x10^{-2}$
COL19A1	-4.57	-0.64	9.40	$3.14x10^{-3}$	$1.31x10^{-2}$
ABCC4	1.59	4.99	9.20	$3.45x10^{-3}$	$1.41x10^{-2}$
WDR4	1.60	2.91	8.94	$3.91x10^{-3}$	$1.56x10^{-2}$
DNASE1L2	2.54	0.23	8.77	$4.23x10^{-3}$	$1.66x10^{-2}$
CYP4F12	-1.60	6.12	8.73	$4.31x10^{-3}$	$1.68x10^{-2}$
RSPO2	-4.58	1.81	8.5	$4.83x10^{-3}$	$1.84x10^{-2}$
SYTL3	-2.14	2.54	8.44	$4.98x10^{-3}$	$1.88x10^{-2}$
RASSF4	-1.51	5.26	8.27	$5.41x10^{-3}$	$2.01x10^{-2}$
SHC2	-2.67	3.78	8.25	$5.47x10^{-3}$	$2.03x10^{-2}$
ZNF831	-3.35	1.20	8.17	$5.67x10^{-3}$	$2.09x10^{-2}$
KRT4	3.35	1.94	7.88	$6.55x10^{-3}$	$2.34x10^{-2}$
NLGN1	-5.95	0.57	7.72	$7.08x10^{-3}$	$2.50x10^{-2}$
APC2	-1.95	1.31	7.29	$8.80x10^{-3}$	$2.96x10^{-2}$
EEF2K	-1.53	5.44	7.08	$9.78x10^{-3}$	$3.20x10^{-2}$
CMKLR1	-1.74	3.67	6.85	$1.10x10^{-2}$	$3.51x10^{-2}$
FAM221B	-3.27	-1.32	6.54	$1.28x10^{-2}$	$3.95x10^{-2}$
PKD2	-1.99	4.78	6.18	$1.55x10^{-2}$	$4.58x10^{-2}$

Supplementary Table 40. Genes with rare germline variants identified in patient TCGA-DC-4745 using 'All vs One' approach. This table lists genes identified through the 'All vs One' approach that harbour rare germline variants in patient TCGA-DC-4745. Genes shown meet the differential expression threshold of |log2 fold change| ≥1.5 and FDR <5%. Variants were retained on filtering criteria for rare population frequency. logFC: log2 fold change in gene expression; logCPM: log2-transformed counts per million across all samples; F: quasi-likelihood F-test statistic; p-value: nominal p-value (uncorrected); FDR: p-value adjusted using Benjamini-Hochberg correction.

	logFC	logCPM	F	p-value	FDR
CFAP57	3.07	0.58	177.72	$1.82x10^{-20}$	$3.59x10^{-18}$
CAPZA2	1.76	6.69	93.23	$2.77x10^{-14}$	$1.22x10^{-12}$
TAF1C	-2.50	5.94	72.22	$3.19x10^{-12}$	$9.01x10^{-11}$
NDUFA13	1.83	6.68	70.77	$4.54x10^{-12}$	$1.24x10^{-10}$
DSG4	5.31	-1.64	62.75	$3.46x10^{-11}$	$7.74x10^{-10}$
CCDC113	2.67	1.78	53.15	$4.71x10^{-10}$	$8.28x10^{-9}$
TFB2M	1.67	3.93	49.28	$1.44x10^{-9}$	$2.28x10^{-8}$
MPP1	1.78	4.59	47.76	$2.25x10^{-9}$	$3.46x10^{-8}$
RIPOR3	-3.76	3.90	44.53	$5.94x10^{-9}$	$8.27x10^{-8}$
SLC4A4	-5.59	7.68	36.49	$7.68x10^{-8}$	$8.31x10^{-7}$
YARS1	1.88	5.73	32.97	$2.51x10^{-7}$	$2.42x10^{-6}$
KCTD1	-3.54	2.78	32.61	$2.85x10^{-7}$	$2.71x10^{-6}$
PLCE1	-2.19	6.83	30.35	$6.28x10^{-7}$	$5.50x10^{-6}$
THEM6	1.64	4.79	29.53	$8.39x10^{-7}$	$7.09x10^{-6}$
UBA7	-2.54	6.21	26.96	$2.13x10^{-6}$	$1.63x10^{-5}$
TTN	-3.36	4.36	25.03	$4.37x10^{-6}$	$3.12x10^{-5}$
LRP3	1.69	4.41	21.40	$1.77x10^{-5}$	$1.09x10^{-4}$
COL11A1	2.87	-0.71	21.24	$1.89x10^{-5}$	$1.15x10^{-4}$
RRP12	2.00	4.44	20.40	$2.63x10^{-5}$	$1.55x10^{-4}$
TTLL6	-4.09	4.10	20.12	$2.95x10^{-5}$	$1.71x10^{-4}$
BRCA1	2.00	3.43	19.67	$3.53x10^{-5}$	$2.01x10^{-4}$
TCTE1	-5.48	-1.27	19.00	$4.63x10^{-5}$	$2.56x10^{-4}$
ALS2CL	-1.84	6.37	17.85	$7.42x10^{-5}$	$3.90x10^{-4}$
CHRNE	-1.94	3.40	16.10	$1.55x10^{-4}$	$7.45x10^{-4}$
CHRD	-2.33	3.05	15.62	$1.90x10^{-4}$	$8.92x10^{-4}$
CCDC180	-2.86	2.51	15.49	$2.00x10^{-4}$	$9.36x10^{-4}$
ZNF596	-1.58	1.95	14.55	$3.02x10^{-4}$	$1.33x10^{-3}$
CHST6	-4.56	0.52	14.48	$3.10x10^{-4}$	$1.37x10^{-3}$
SLC27A6	-4.52	-0.32	14.38	$3.25x10^{-4}$	$1.42x10^{-3}$
CHL1	-3.27	3.93	13.53	$4.71x10^{-4}$	$1.96x10^{-3}$
UPK3B	-4.22	0.37	12.63	$7.05x10^{-4}$	$2.78x10^{-3}$

CMYA5	-4.04	1.96	12.28	$8.23x10^{-4}$	$3.18x10^{-3}$
COL6A6	-6.57	-2.71	11.32	$1.27x10^{-3}$	$4.65x10^{-3}$
FLRT1	-3.46	1.86	11.00	$1.48x10^{-3}$	$5.28x10^{-3}$
BLM	1.71	2.44	10.67	$1.72x10^{-3}$	$6.01x10^{-3}$
ZNF423	-2.36	2.86	10.56	$1.82x10^{-3}$	$6.30x10^{-3}$
CYP21A2	-8.79	-1.00	10.47	$1.89x10^{-3}$	$6.51x10^{-3}$
STEAP4	-3.40	3.61	9.98	$2.38x10^{-3}$	$7.92x10^{-3}$
SLC7A14	-6.40	-0.04	9.93	$2.44x10^{-3}$	$8.10x10^{-3}$
GPR146	-2.16	2.54	9.42	$3.10x10^{-3}$	$9.95x10^{-3}$
SOSTDC1	-2.62	2.77	9.38	$3.16x10^{-3}$	$1.01x10^{-2}$
DOCK8	-1.69	4.56	8.97	$3.85x10^{-3}$	$1.20x10^{-2}$
CGN	-1.66	7.85	8.27	$5.42x10^{-3}$	$1.61x10^{-2}$
NACAD	-3.66	2.27	8.01	$6.15x10^{-3}$	$1.79x10^{-2}$
EFS	-2.67	3.31	7.54	$7.75x10^{-3}$	$2.16x10^{-2}$
XAF1	-2.35	4.88	6.84	$1.10x10^{-2}$	$2.90x10^{-2}$
VWA3A	-4.23	-1.39	6.80	$1.13x10^{-2}$	$2.96x10^{-2}$
HPR	-7.52	-2.05	6.25	$1.49x10^{-2}$	$3.72x10^{-2}$
ADARB1	-2.99	4.66	6.12	$1.59x10^{-2}$	$3.93x10^{-2}$
NFASC	-2.16	5.08	6.07	$1.63x10^{-2}$	$4.02x10^{-2}$
UNC5D	-2.93	-0.02	5.93	$1.76x10^{-2}$	$4.26x10^{-2}$
